# Can we ‘feel’ the temperature of knowledge? Modelling scientific popularity dynamics via thermodynamics

**DOI:** 10.1371/journal.pone.0244618

**Published:** 2021-02-11

**Authors:** Luoyi Fu, Dongrui Lu, Qi Li, Xinbing Wang, Chenghu Zhou

**Affiliations:** 1 Electronic Engineering, Shanghai Jiao Tong University, Shanghai, China; 2 Institute of Geographical Sciences and Natural Resources Research, Chinese Academy of Sciences, Beijing, China; University of Chicago, UNITED STATES

## Abstract

Just like everything in nature, scientific topics flourish and perish. While existing literature well captures article’s life-cycle via citation patterns, little is known about how scientific popularity and impact evolves for a specific topic. It would be most intuitive if we could ‘feel’ topic’s activity just as we perceive the weather by temperature. Here, we conceive knowledge temperature to quantify topic overall popularity and impact through citation network dynamics. Knowledge temperature includes 2 parts. One part depicts lasting impact by assessing knowledge accumulation with an analogy between topic evolution and isobaric expansion. The other part gauges temporal changes in knowledge structure, an embodiment of short-term popularity, through the rate of entropy change with internal energy, 2 thermodynamic variables approximated via node degree and edge number. Our analysis of representative topics with size ranging from 1000 to over 30000 articles reveals that the key to flourishing is topics’ ability in accumulating useful information for future knowledge generation. Topics particularly experience temperature surges when their knowledge structure is altered by influential articles. The spike is especially obvious when there appears a single non-trivial novel research focus or merging in topic structure. Overall, knowledge temperature manifests topics’ distinct evolutionary cycles.

## Introduction

### Text

Scientific impact assessment helps shape scientific development from aspects including investment [1, 2], promotion policy [3, 4] and individual career [5, 6]. Thanks to its significance and widespread applications, measuring scientific impact has always been one of the most discussed topics in communities of all disciplines. Citation-based analysis always occupies a predominant role for impact assessment because of the quantitative characteristics of citations and more importantly, the positive correlation between citation and scientific influence [7, 8]. For an article, citation dynamics reveals its temporal evolution of impact [9–11] and popularity [12]. For a researcher, the evolution of individual citation statistics portraits his or her activity [13], scholar impact dynamics [14–16] and research interest pattern [17]. For a scientific topic, however, individual or article citation dynamics modeling fails to characterize its life-cycle because this one-dimensional indicator is not capable of exploiting the interplay among academic entities. This raises a fundamental question: how to depict the rise and fall of a scientific topic by leveraging its citation information?

**Fig 1:**
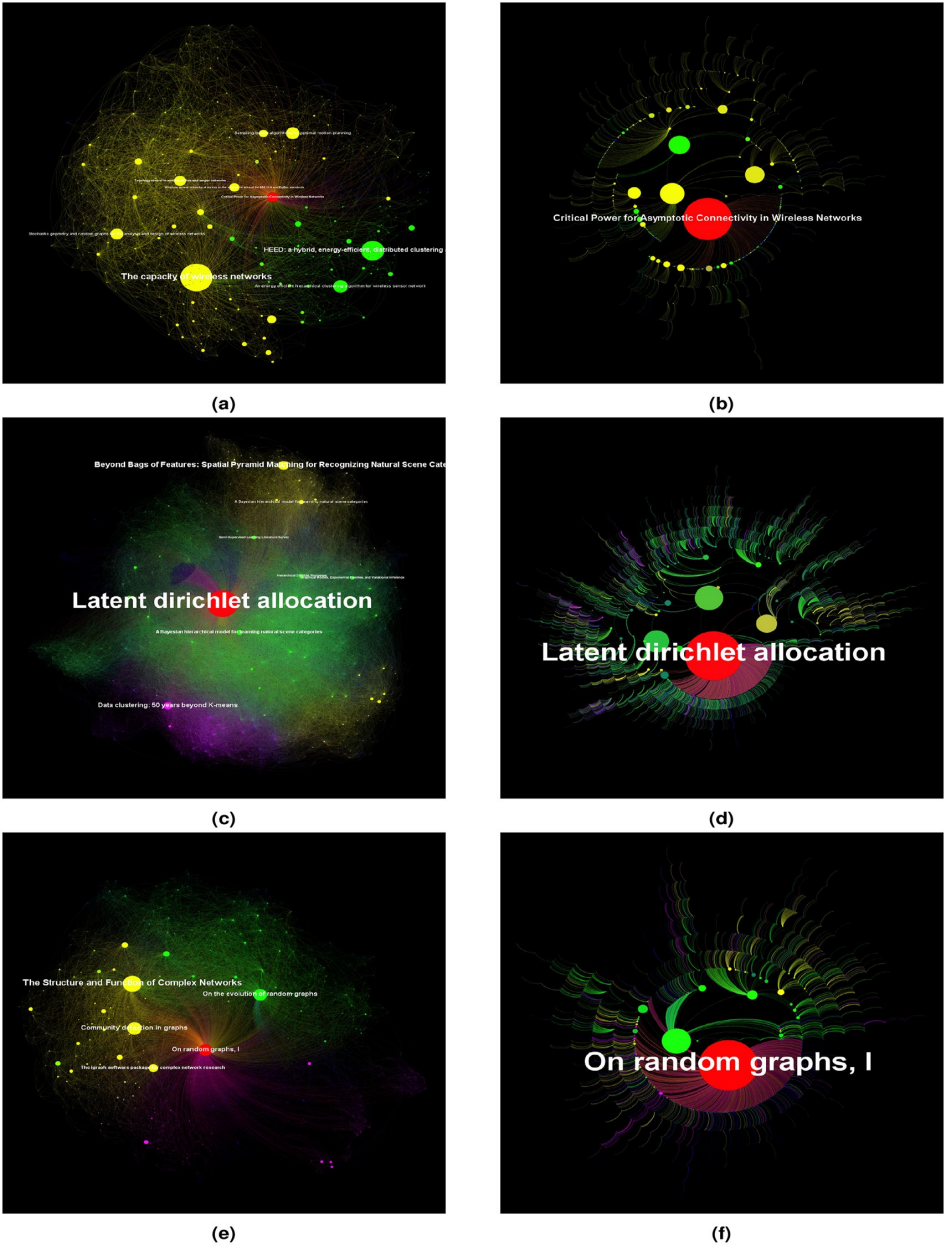
Comparison between galaxy map and topic skeleton tree. In galaxy map: Node size and label size are proportional to article’s total citation count. Only the most-cited papers are labelled with titles. The node representing the pioneering work is colored in red. Other nodes are colored by their positions under the ForceAltas layout algorithm. Nodes in the same cluster take a same colour (yellow, green, blue or pink). In topic skeleton tree: Node size (except for the node representing the pioneering work) is proportional to article’s structure entropy. The size of the node that stands for the pioneering work is twice the maximum size of the other nodes. Node colour is the same as in galaxy map. Only pioneering work is labeled by its title. (a,b) Topic led by ‘Critical Power for Asymptotic Connectivity in Wireless Networks’. (a) Galaxy map. Numerous child papers, especially ‘The capacity of wireless networks’ and ‘HEED: a hybrid, energy-efficient, distributed clustering approach for ad hoc sensor networks’, have outperformed the pioneering work. (b) Skeleton tree. After initial development, the topic has found two research focus. (c,d) Topic led by ‘Latent dirichlet allocation’. (c) Galaxy map. The pioneering work has a dominant influence. (d) Skeleton tree. Three research directions have derived directly from the pioneering work. (e,f) Topic led by ‘On random Graphs, I’. (e) Galaxy map. Two influential child papers, ‘On the evolution of random graphs’ and ‘The Structure and Function of Complex Networks’ seem to split the topic into two parts. (f) Skeleton tree. The pioneering work has inspired in particular one school of thought. There is no significant division in topic’s knowledge structure.

The first step to answer this question is to define scientific topic and then to find an appropriate way to describe it. A scientific topic is in fact a complex network comprising of articles that have similar research interests. As citation is able to display the interaction among articles, we can thus define and represent a scientific topic by its citation network. Topic citation network is a directed graph where nodes represent articles and edges symbolize citations. By retrieving and integrating academic data from renowned databases including but not limited to DBLP, arXiv, Elsevier and Springer, we identified 47310 articles that have gained over 1000 citations and have had a non-trivial influence within their research fields. These articles were published between 1800 and 2019 and their research interests cover 294 domains in 16 disciplines: History, Computer science, Environmental science, Geology, Psychology, Mathematics, Physics, Materials science, Philosophy, Biology, Medicine, Sociology, Art, Economics, Chemistry and Political science. A detailed catagorical information can be found in the folder entitled “topic all levels and classifications (galaxy map and skeleton tree)” at https://github.com/drlisette/knowledge-temperature/tree/master/data. Some of them created new topics while others made major breakthroughs in existing fields. Their immense contribution and inspiration to subsequent researches has made them each a leader in their field of research. To this end, we refer to these papers as pioneering works and define a scientific topic led by each to be a citation network that consists of the pioneering work, child papers, which are all the articles that directly cite the pioneering work, and all the citations among them. We visualize our scientific topics with a graph that we call galaxy map. Galaxy map not only highlights the most influential child papers along with the pioneering work, but also does a preliminary clustering within the topic (Fig 1(a), 1(c) and 1(e)). We find that while some pioneering works still have an overwhelming impact in the scientific topics they founded, quite a few have several child papers who have established an authority comparable or even greater than themselves. Furthermore, in some of our examples, these prominent child papers seem to have transformed the original topic into multiple new topics (Fig 1(e)). Much as galaxy map gives a nice overview of scientific topic’s current status, the temporal evolution of scientific topic needs to be further depicted. With this regard, we go beyond the galaxy map representation and dig deeper into the topic citation network for a more intuitive perception of topic’s flourishing dynamics.

Since we interpret scientific topics through their citation pattern, topic evolution is reflected by the development of topic citation network. Complicated academic citation networks are springing up all across the science community as a result of the explosive research activity growth, both in and across disciplines, and the prevalence of larger teams [18, 19]. The representation and characterization of complex network has attracted a huge amount of efforts, among which an appeal to statistical thermodynamics stands out as a principled school of thought [20]. Some studies at the beginning of this century reveal the intimate connections between thermodynamic quantities and complex network dynamics [21]. Recently, more literature has succeeded in characterizing natural networks [22], neuron networks [23] and biological networks [24] through thermodynamic approaches. In particular, thermodynamic temperature is able to capture critical events in evolving networks [25]. These prior works inspired us in that heat corresponds with popularity and moreover, temperature quantifies partly our body feelings of weather. It would be most direct and intuitive if we could ‘feel’ topic vigor in the same way as we perceive the weather. Motivated by this thought, we try to depict the flourishing and perishing of scientific topics by measuring their knowledge temperature, a quantity designed to portrait topic impact and popularity evolution by leveraging the rich structural information hidden in citation networks.

**Fig 2:**
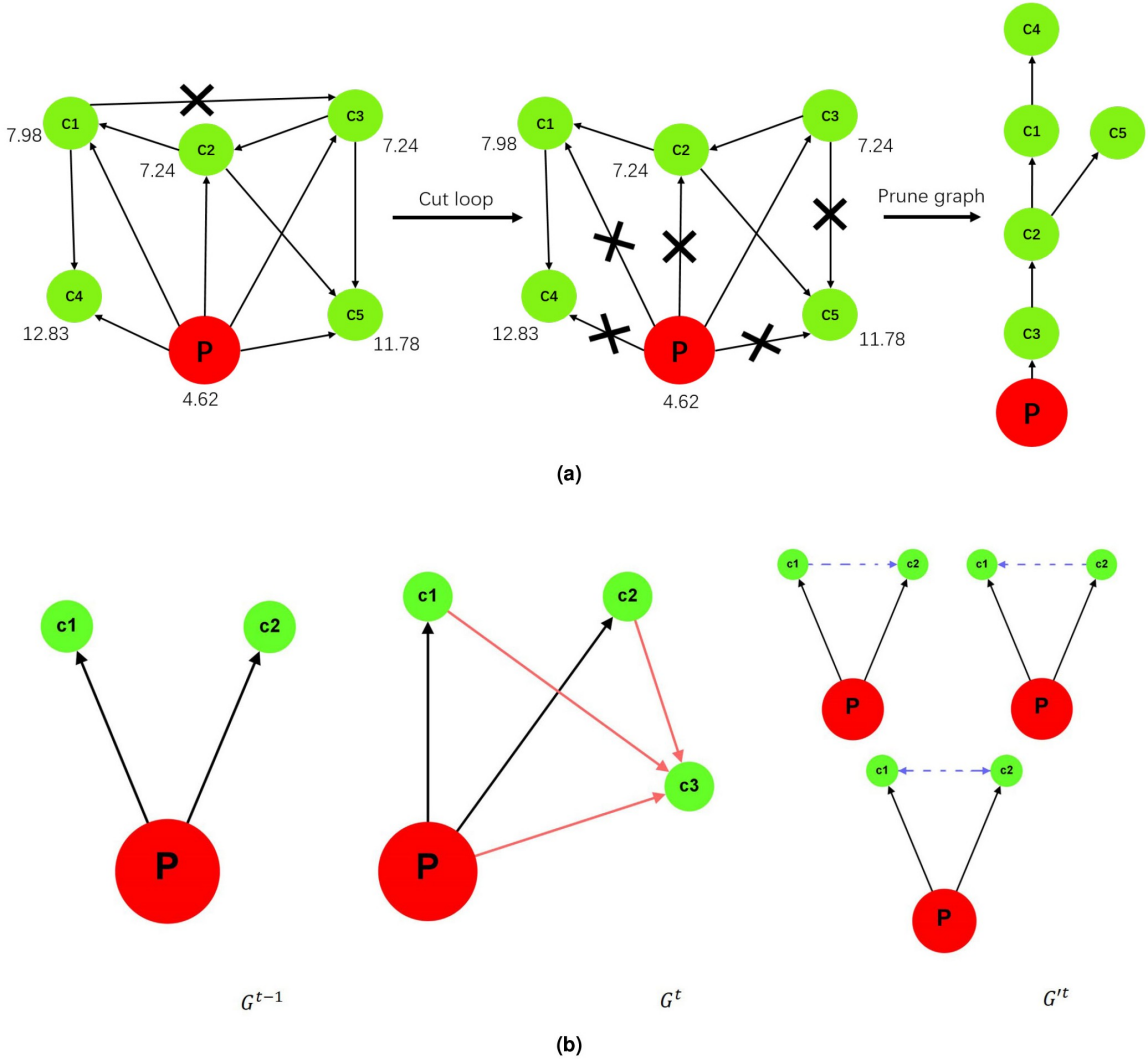
Skeleton tree extraction and graph shrinking demo. The red node “P” is the pioneering work and the green nodes are child papers. A directed edge from A to B represents “B cites A”. (a) Skeleton tree extraction. Leftmost: topic citation network. Rightmost: topic skeleton tree. The importance of a citation is determined by the difference of *ReductionIdx* between a paper and its reference. The bigger the difference, the more trivial a citation is. Each node’s *ReductionIdx* is indicated by the value beside. From left to middle: loop cutting. *c*1, *c*2 and *c*3 cite one another. Remove one of the three citations to eliminate the loop structure. The citation between *c*1, *c*2 and the one between *c*1 and *c*3 are of equal importance and are both less crucial than the one between *c*1 and *c*2. In this case, remove one of the two citations randomly. Here we cut the citation between *c*1 and *c*3. From middle to right: graph pruning. Remove redundant citations and only keep the most meaningful citation for every child paper. Similarly, in case of equivalence, remove a citation randomly. (b) Graph shrinking example for $T_{structure}^{t}$ computation. *c*3 arrives between timestamp *t* − 1 and *t* and cites all papers in the topic. Its citation pattern suggests that *c*1 and *c*2, disconnected in *G*^*t*−1^, have relatively close connections in their research content. Remove *c*3 and add virtual citation(s) between *c*1 and *c*2 according to the general rule where the younger virtually cites the older. If *c*1 and *c*2 were published in the same year, they virtually cite each other in *G*^*t*^’s counterpart after shrinking, *G*′^*t*^.

Knowledge temperature essentially depends on 2 factors: the accumulation of topic knowledge and the advancement of topic knowledge structure. As knowledge is a sublimation of information and duplicated information is no longer valuable to knowledge generation, measuring knowledge quantity boils down to evaluating the volume of non-overlapped, or useful information. The latter, however, can be estimated by examining paper similarity, which essentially involves determining citation significance. As for knowledge structure, it is also closely related to the question whether a citation is important for an article. Therefore, in order to address the key issue in knowledge temperature conception: citation importance judgement, we extracted skeleton tree for each topic (Fig 1(b), 1(d) and 1(f)). While galaxy map visualizes the entire topic citation network, skeleton tree captures the most essential idea inheritance within the topic by preserving the most valuable citation for every child paper. In particular, we are able to answer 2 fundamental questions by tracing down a path in skeleton tree: from what thought an idea is greatly inspired and what new idea it has directly inspired. From another perspective, skeleton tree demonstrates certain clustering effect in its leaves as it puts intimately related articles together. We employed graph embedding techniques to extract topic skeleton tree. By representing all of the articles in a high dimensional space, we calculated the difference between each pair of article, *DiffIdx*, and further computed *ReductionIdx* for every paper based on structural information. *ReductionIdx* measures the similarity between an article and the whole topic and we use it to evaluate the importance of every citation. Detailed description of *ReductionIdx* can be found in S1 File Section S1.1. Skeleton tree extraction follows two principles: first and foremost, keep at most one citation for every article except the pioneering work and second, ensure topic’s global connectivity. The extraction process consists of 2 steps: firstly, remove loops in the topic citation network and secondly, prune the network by leaving out less important citations of every child paper. In an ideal topic skeleton tree, every article, except the pioneering work, has exactly one citation and we are able to reach every article from the pioneering work (Fig 2(a)). In addition, we further calculated structure entropy [26] for every article and for the entire topic based on topic skeleton tree. For an article, its structure entropy indicates its authority within the topic. For a topic, its structure entropy measures the information hidden in its citation pattern. Because the extraction process involves a thorough investigation into citation network structure, topic skeleton tree serves as an indispensable tool for our knowledge temperature design and for the heat distribution visualization within the topic.

We evaluated topic knowledge temperature from 2 aspects: the increase in topic knowledge and recent structural change in topic knowledge. For each perspective, we made an analogy between topic citation network *G*^*t*^ = (*V*^*t*^, *E*^*t*^) and a thermodynamic system and used the system’s thermodynamic temperature as a popularity score. Due to their different focuses and objectives, there is no connection between the two analogies. They are independent from each other. At timestamp *t*, we add up the popularity scores obtained by *G*^*t*^ in distinct aspects and define topic knowledge temperature *T*^*t*^ as:

$$\begin{array}{c} T^{t}=T_{growth}^{t}+T_{structure}^{t} \end{array}$$ (1)

where $T_{growth}^{t}$ measures knowledge increment and $T_{structure}^{t}$ estimates the magnitude of changes in knowledge structure between 2 consecutive timestamps.

For $T_{growth}^{t}$, we made an analogy between scientific topic and ideal gas. We assumed that the restraints a topic faces during its development remain at a constant level. The corresponding thermodynamic hypothesis is that the pressure remains invariant. While temperature is a derived function of entropy and internal energy, it can also be expressed in terms of other state variables for ideal gas. We opted to derive temperature by volume *V*_*t*_ and mole number *n*_*t*_ as these two variables are more intuitive in our design. We set *V*_*t*_, *n*_*t*_ to be the amount of total information and the amount of duplicated information a topic has by the end of timestamp *t*. Specifically, *V*_*t*_ = |*V*^*t*^| and *n*_*t*_ = |*V*^*t*^| − *UsefulInfo*^*t*^. *UsefulInfo*^*t*^, *G*^*t*^’s useful information, is derived edge by edge based on *G*^*t*^’s skeleton tree, *Tree*^*t*^. In addition, we defined initial entropy *S*_0_ to be the structure entropy of *G*^0^. We initialized $T_{growth}^{t}$ by combining 2 ideal gas’s internal energy expressions. $T_{growth}^{0}$ is defined in terms of initial entropy *S*_0_, initial volume *V*_0_ and initial mole number *n*_0_. We updated $T_{growth}^{t}$ via ideal gas state equation, *PV* = *nRT*. With pressure *P* being invariant and *R* being constant, the variation of $T_{growth}^{t}$ is governed by the dynamics of mole number *n*_*t*_ and topic volume *V*_*t*_. The internal energy *U*_*t*_ can further be defined using the expression $U_{t}=cn_{t}T_{growth}^{t}$. By making the hypothesis *n*_*t*−1_ = *n*_*t*_ and then envisaging a reversible path connecting the two states (*P*,*V*_*t*−1_,*n*_*t*−1_, $T_{growth}^{t-1}$) and (*P*,*V*_*t*_,*n*_*t*_, $T_{growth}^{t}$), we were able to iteratively compute the entropy *S*_*t*_ with an integration over temperature and volume: $dS=nc_{v}\frac{dT}{T}+nR\frac{dV}{V}$. Closed-form update expression of *S*_*t*_ can further be derived under the assumption that the molar specific heat capacity *c*_*v*_ is a constant. Although *S*_0_ and *S*_*t*_ look different, they are closely related. While *S*_0_ quantifies the information hidden in topic citation pattern from a microscopic perspective, by probing into the surrounding structure of every article, *S*_*t*_ depicts this part of information from a macroscopic point of view with two state variables, temperature and volume. Detailed $T_{growth}^{t}$ modelling information can be found in S1 File Section S1.3.1. From a macroscopic view of information and knowledge, $T_{growth}^{t}$ increases when topics succeed in accumulating distinct, or useful information, the knowledge source for the future. Intuitively, promising topics are able to attract a steady or even growing inflow of new information. On the contrary, staggering topics consume more useful information than they receive and their potential eventually drops. A rising $T_{growth}^{t}$ indicates an increasingly solid and rich knowledge base and thus reflects a topic’s growing impact. Furthermore, an accelerating increase in $T_{growth}^{t}$ suggests a topic’s greater capability in useful information collection and thus its faster gain in fame.

Inspired by the temperature design in prior work [25, 27], we computed $T_{structure}^{t}$ by making an analogy between *G*^*t*^’s evolution between two adjacent timestamps and an isochoric state of change of a general thermodynamic system. We defined the system’s volume to be *G*^*t*^’s node number. The analogy is legitimate as long as the node number is fixed, which unfortunately does not hold for *G*^*t*^. In order to solve this issue, we designed a graph shrinking algorithm that transforms *G*^*t*^ into *G*′^*t*^, where the nodes in *G*^*t*^ that arrive between timestamp *t* − 1 and *t* are converted to virtual edges among nodes in *G*^*t* − 1^ (Fig 2(b)). We derived $T_{structure}^{t}$ from the changes in internal energy and in entropy of the thermodynamic system:

$$\begin{array}{c} T_{structure}^{t}=|\frac{dU_{t}}{dS_{t}}|=|\frac{U_{t}^{\prime }-U_{t-1}}{S_{t}^{\prime }-S_{t-1}}| \end{array}$$ (2)

We defined entropy *S*_*t*−1_, $S_{t}^{\prime }$ to be the von Neumann entropy [28] of *G*^*t*−1^ and *G*′^*t*^, the weighted reduced graph of *G*^*t*^ obtained from the graph shrinking algorithm. *S*_*t*−1_, $S_{t}^{\prime }$ are approximated by node degree. We set internal energy *U*_*t*−1_, $U_{t}^{\prime }$ to be the ratio between edge number and node number of *G*^*t*−1^ and *G*′^*t*^ respectively. Different from $T_{growth}^{t}$ which focuses more on continual knowledge increment, $T_{structure}^{t}$ is designed to capture recent critical events and hence assesses topic’s short-term popularity. Detailed $T_{structure}^{t}$ modelling information can be found in S1 File Section S1.3.2.

Among all the topics, we identified 16 representative topics to conduct our knowledge temperature experiment. The pioneering works of these topics were published between 1959 and 2014 and their research interests fall in domains including machine learning, wireless network, graph theory, biology and physics. These topics have sizes ranging from over 1000 articles and approximately 5000 citations to more than 31000 articles and nearly 200 thousand citations. A detailed description of the selected topics can be found in the folder entitled “knowledge temperature data” at https://github.com/drlisette/knowledge-temperature/tree/master/data. By observing topic skeleton tree and topic knowledge temperature together, we found that *T*^*t*^’s evolution is consistent with topic skeleton tree’s development and that *T*^*t*^’s dynamics well depicts topic flourishing, with $T_{growth}^{t}$ quantifying knowledge accumulation and $T_{structure}^{t}$ reflecting knowledge structure shift. In particular, we noted that influential child papers usually play an important role in boosting both *T*^*t*^’s components. They are crucial to topic’s thriving in that they help topics accumulate useful information and generate knowledge in multiple aspects. However, there is a big variety in the duration between their publication time and the moment when their contribution becomes perceptible [29].

**Fig 3:**
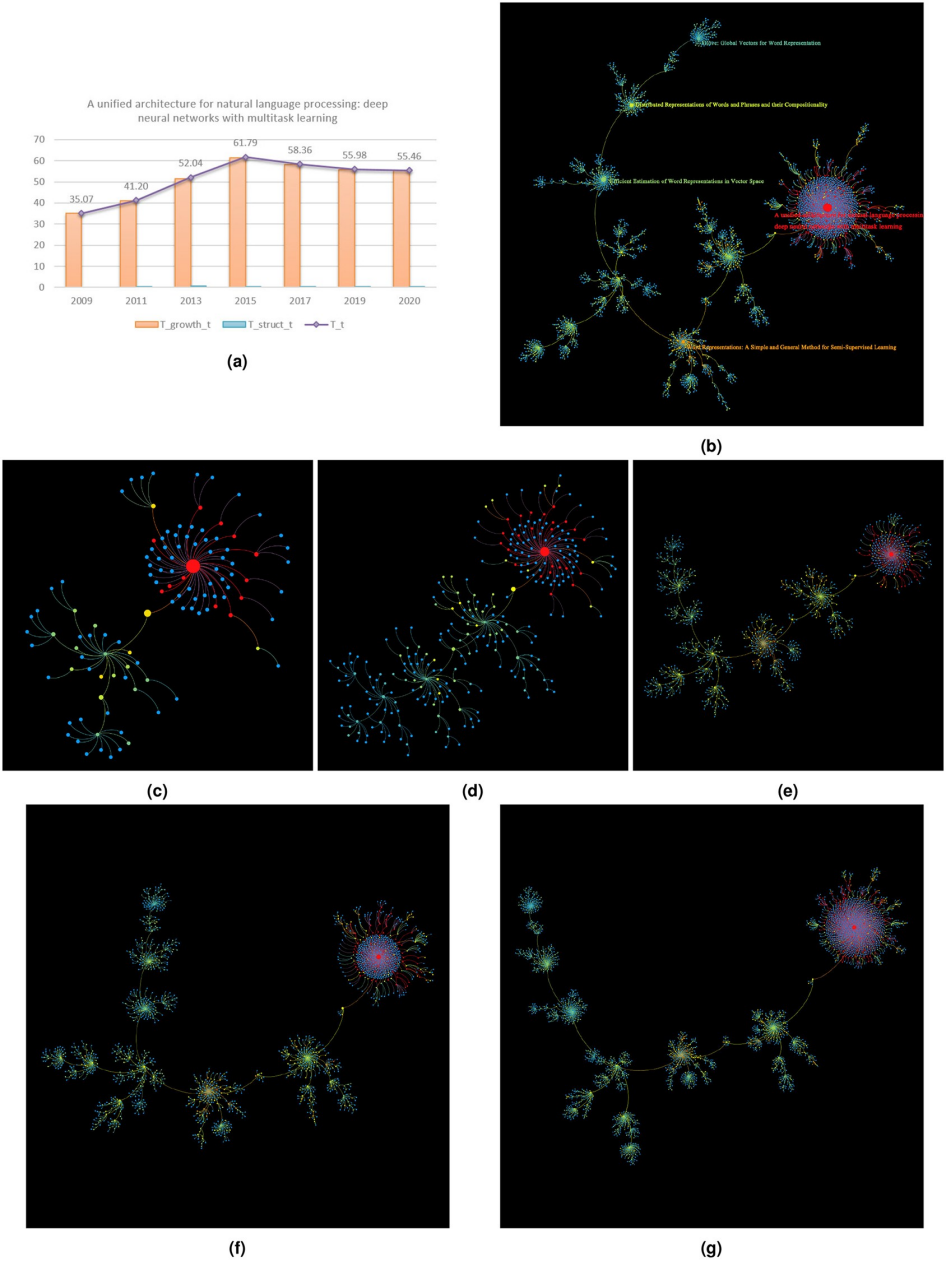
Knowledge temperature (especially *T*^*t*^ and $T_{growth}^{t}$) and skeleton tree evolution of topic led by ‘A unified architecture for natural language processing: Deep neural networks with multitask learning’. Node color corresponds to paper knowledge temperature, with red being the hottest, yellow the average level and blue the coldest. Node size (except for the node representing the pioneering work) is proportional to (re-scaled) structure entropy. The size of the node that stands for the pioneering work is twice the maximum size of the other nodes. (a) Knowledge temperature evolution. $T_{growth}^{t}$ dominates *T*^*t*^. (b) Current topic skeleton tree. The pioneering work and 4 most top-cited papers within the topic are labelled by title. (c,d,e) Topic skeleton tree by the end of 2011, 2013 and 2015. The thriving period is characterized by a fast-growing skeleton tree where small new clusters emerge and existing branches become increasingly robust. (f,g) Topic skeleton tree by the end of 2017 and 2019. The stagnation period is reflected by a decelerating growth and an almost fixed tree shape.

$T_{growth}^{t}$ varies smoothly and determines the overall trend of *T*^*t*^ (Fig 3(a)). A big rise in $T_{growth}^{t}$ corresponds most often with a significant increase in topic size. Typically, during such periods, some child papers start to gain popularity and collect a non-trivial number of citations within the topic. They help the pioneering work maintain the topic visibility [9, 30]. Their attractiveness to new ideas, added to that of the pioneering work, helps topic accumulate useful information and eventually enrich topic knowledge pool (Fig 3(b)). A direct consequence of this phenomenon, visible from skeleton tree, is a fortification of existing knowledge structure, sometimes accompanied by a mild extension (Fig 3(c)–3(e)). Nonetheless, an ever-growing topic scale is not a guarantee for thriving periods. For instance, $T_{growth}^{t}$ of topic led by ‘Critical Power for Asymptotic Connectivity in Wireless Networks’ has been on the decrease since 2011 despite a continuous growth in article number. This corresponds to the fact that almost all of the influential child papers within the topic were published no later than 2005. The lack of new, promising ideas and remarkable extensions to existing researches afterwards makes the topic lose community’s attention and results in the topic’s demise. As for topic led by ‘A unified architecture for natural language processing: deep neural networks with multitask learning’, its decline in $T_{growth}^{t}$ since 2015 is somewhat atypical. The decrease is owing to the emergence of popular child papers published between 2013 and 2014 that largely excel their parent. Child papers ‘Efficient Estimation of Word Representations in Vector Space’, ‘Distributed Representations of Words and Phrases and their Compositionality’ and ‘Glove: Global Vectors for Word Representation’ have each attracted around 600 citations within the topic, while their total citations have all surpassed 8000, much greater than their antecedent whose citation count still remains below 3000. They have had such big achievements that they have become the authorities in the domain. Consequently, they have won over the attention of subsequent studies, which in turn affects the knowledge accumulation of the topic created by their parent paper. We observe that articles published after 2016 in the topic have not had a comparable development. This confirms partly the shadowing effect caused by the prominent child papers mentioned above.

**Fig 4:**
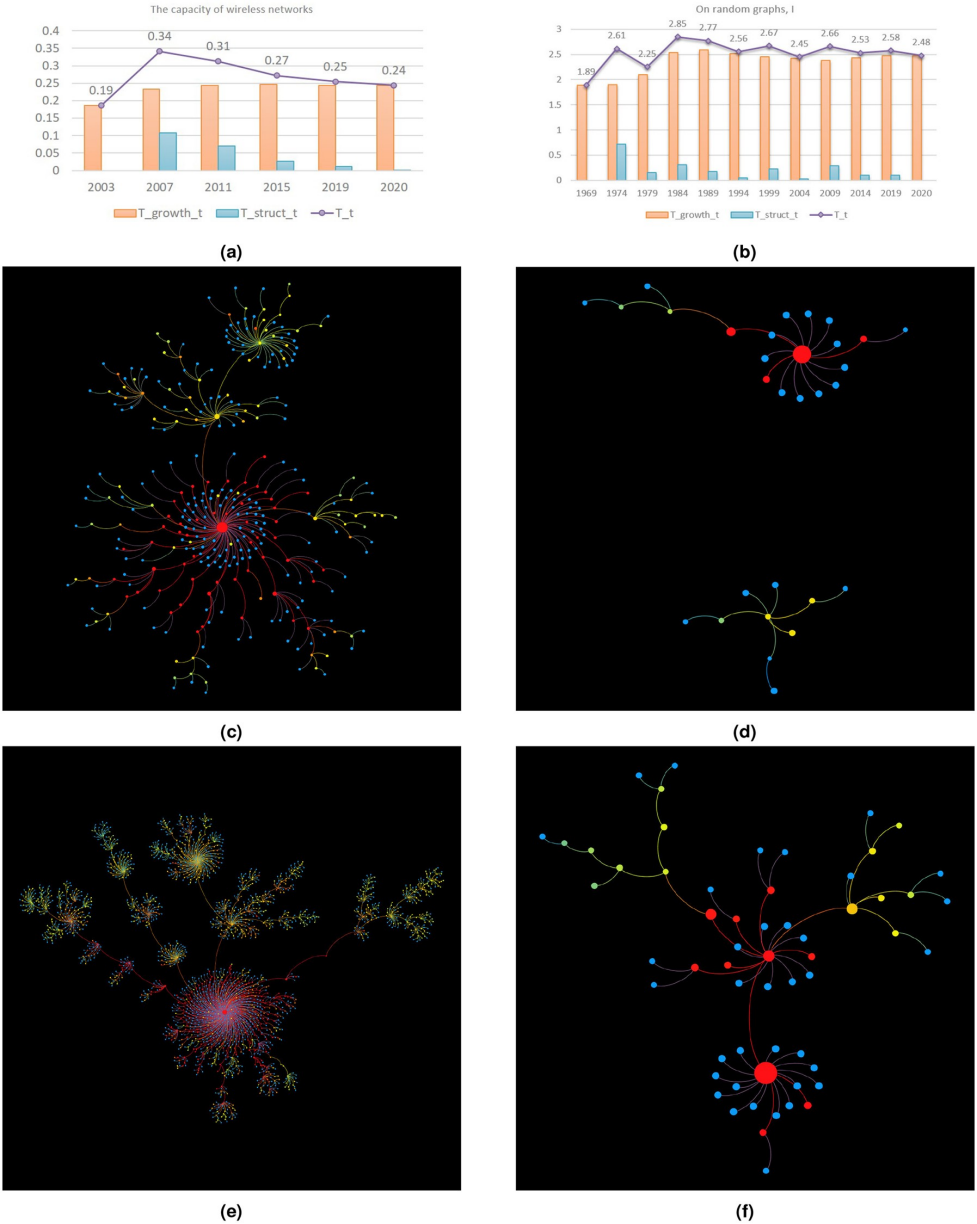
Knowledge temperature (especially *T*^*t*^ and $T_{structure}^{t}$) and skeleton tree evolution of topics led by ‘The capacity of wireless networks’ (CWN) and ‘On random graph, I’ (RG). Node color corresponds to paper knowledge temperature, with red being the hottest and blue the coldest. Node size (except for the node representing the pioneering work) is proportional to (re-scaled) structure entropy. The size of the node that stands for the pioneering work is twice the maximum size of the other nodes. (a,b) Knowledge temperature evolution. $T_{structure}^{t}$ accounts for *T*^*t*^’s fluctuations. (c,e) Skeleton tree of the topic led by CWN by the end of 2003 and 2007. Advancements are visible in all directions. In particular, the gravity shift in the tree implies the emergence of new research focus, which yields a soar in $T_{structure}^{t}$. (d,f) Skeleton tree of the topic led by RG by the end of 1979 and 1984. Article ‘On the evolution of random graphs’ published in 1984 fuses the previously separated parts due to an atypical citation from an older article ‘On the existence of a factor of degree one of a connected random graph’. The merge in topic knowledge structure pushed up $T_{structure}^{t}$ during that period.

$T_{structure}^{t}$, unlike $T_{growth}^{t}$, can vary greatly over time. It usually accounts for important fluctuations of *T*^*t*^ (Fig 4(a) and 4(b)). A high $T_{structure}^{t}$ usually marks one of the following 2 events: the formation of sub-topics and the fusion of sub-topics. The first event is a consequence of the arrival of rising stars in the topic. These articles, later proven influential to the topic evolution, either introduce a single novel research focus or multiple research directions. Sometimes, newly developed research directions prove to be a big success and start to defy topic authorities by attracting most new articles’ attention. In this case, we can observe a gravity shift in topic skeleton tree, with new branches and clusters developing much faster than the previously dominating ones (Fig 4(a), 4(c) and 4(e)). The second event takes place when there is subsequent literature uniting prior works’ research. More specifically, the sub-topic merge occurs when there appears some unusual citations where an old article cites a young one and that the young article is crucial to topic development (Fig 4(b), 4(d) and 4(f)). Both the emergence of a single non-trivial research focus and the sub-topic merge can cause an obvious spike in $T_{structure}^{t}$. For instance, topic led by ‘Neural Networks for Pattern Recognition’ had a sudden $T_{structure}^{t}$ increment when child paper ‘A Tutorial on Support Vector Machines for Pattern Recognition’ established a third sub-topic direction. In topic led by ‘On random graphs, I’, prominent child paper ‘On the evolution of random graphs’ fuses prior works’ ideas and changed topic landscape. However, the heat brought by such critical events are ephemeral. In the long run, their impact on topic’s life-cycle is eventually reflected by the knowledge accumulation process, which is quantified by $T_{growth}^{t}$.

In the occurrence of sub-topic merge, topic knowledge temperature increases. Yet the situation is not that simple when a topic splits into multiple directions. The dynamics of topic knowledge temperature depends on the property of prominent child papers who lead novel research branches. If they are developmental papers, which means they mainly amplify the impact of prior work, then topic knowledge temperature will goes up. If they are disruptive papers, which means their novelty overshadows the achievements of prior work, then topic knowledge temperature will stagger or even go down. Whether a child paper is developemental or disruptive can be judged by the ratio between its in-topic citation count and its total citation count. For example, as is already mentioned above in $T_{growth}^{t}$’s discussion, the most popular child papers of the topic led by ‘A unified architecture for natural language processing: deep neural networks with multitask learning’ are all distruptive given that their in-topic citations only account for a small fraction of their total citation count. As a result, we observe a decrease in topic knowledge temperature despite the topic’s continuous expansion (Fig 3(a)). As for topic led by ‘The capacity of wireless networks’, however, its prominent child papers are either developmental or balanced between novelty and idea inheritance. For instance, more than 50% of total citations of child papers ‘Mobility increases the capacity of ad hoc wireless networks’ (published in 2002) and ‘A network information theory for wireless communication: scaling laws and optimal operation’ (published in 2004) belong to the topic. More than 40% of ‘Capacity of Ad Hoc wireless networks”s citations take place in the topic. Therefore, their arrival enriches topic knowledge, extends knowledge structure and contributes to the topic’s flourishing during 2003 and 2007 (Fig 4(a)). Prominent child papers of the topic led by ‘The capacity of wireless networks’ can be found at S1 File Section S2.2.2.

**Fig 5:**
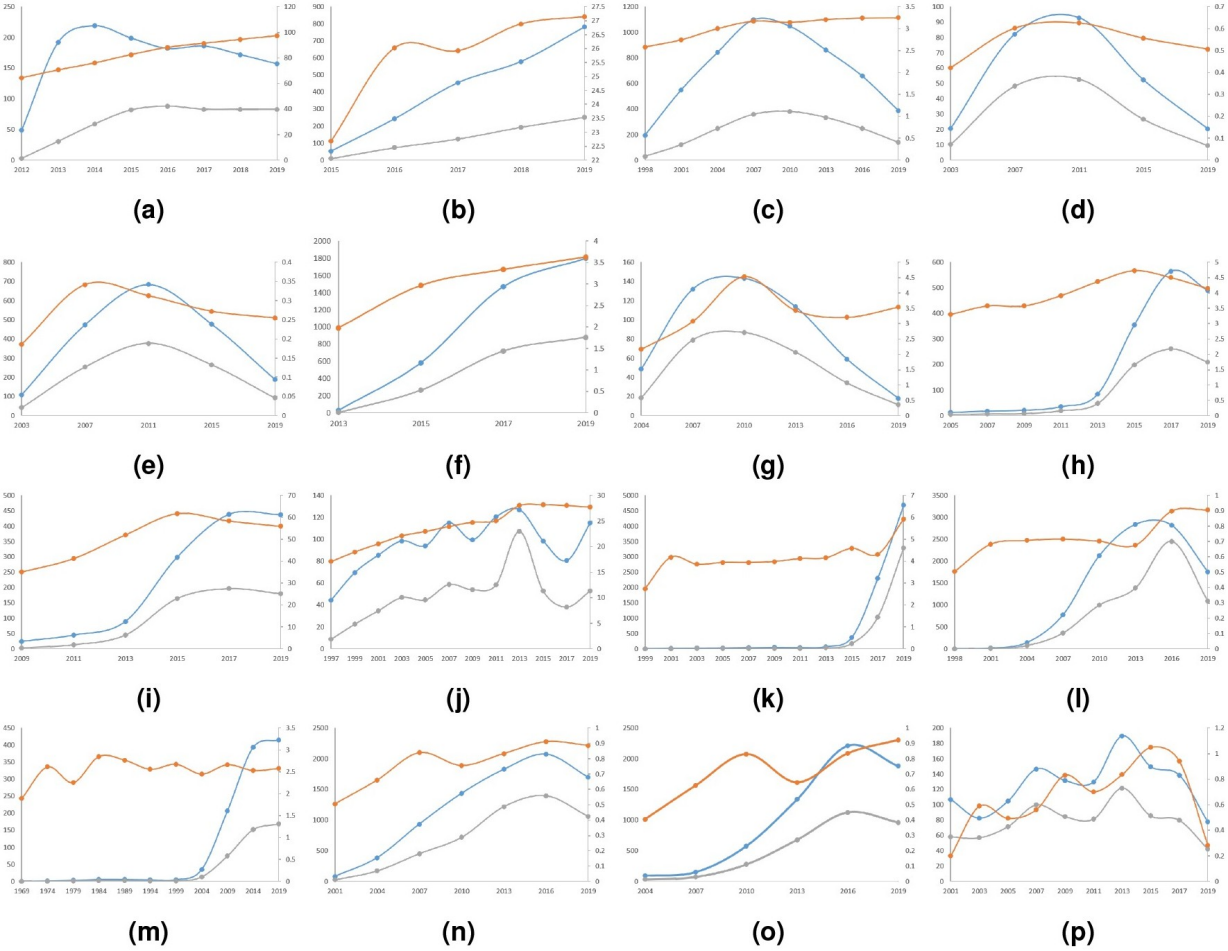
Knowledge temperature, average annual publication number between timestamps and average annual useful information increment between timestamps for 16 topics. Blue line: average annual publication number, $\text{value}=\frac{V^{t}-V^{t-1}}{{year}^{t}-year^{t-1}}$. Orange line: knowlegde temperature *T*^*t*^. Grey line: average annual useful information increment, $\text{value}=\frac{{UsefulInfo}^{t}-{UsefulInfo}^{t-1}}{{year}^{t}-year^{t-1}}$. Left axis is for average annual publication number and average annual useful information increment. Right axis is for knowledge temperature. (a) Topic led by ‘Regulatory T Cells: Mechanisms of Differentiation and Function’. (b) Topic led by ‘Empirical Evaluation of Gated Recurrent Neural Networks on Sequence Modeling’. (c) Topic led by ‘Neural networks for pattern recognition’. (d) Topic led by ‘Critical Power for Asymptotic Connectivity in Wireless Networks’. (e) Topic led by ‘The capacity of wireless networks’. (f) Topic led by ‘Efficient Estimation of Word Representations in Vector Space’. (g) Topic led by ‘Coverage problems in wireless ad-hoc sensor networks’. (h) Topic led by ‘A neural probabilistic language model’. (i) Topic led by ‘A unified architecture for natural language processing: deep neural networks with multitask learning’. (j) Topic led by ‘Bose-Einstein condensation in a gas of sodium atoms’. (k) Topic led by ‘Long short-term memory’. (l) Topic led by ‘Particle swarm optimization’. (m) Topic led by ‘On random graphs, I’. (n) Topic led by ‘Collective dynamics of ‘small-world’ networks’. (o) Topic led by ‘Latent dirichlet allocation’. (p) Topic led by ‘A FUNDAMENTAL RELATION BETWEEN SUPERMASSIVE BLACK HOLES AND THEIR HOST GALAXIES’.

Furthermore, we compared topic knowledge temperature dynamics to the variations of topic’s average annual scientific publication number and topic’s average annual useful information increment between timestamps (Fig 5). Average annual useful information increment is largely determined, though not always, by the size of average annual publication. During periods when a topic sees a decline in its publication number, we observe either a slow-down or a drop in topic knowledge temperature. During periods when a topic accelerates its expansion, topic knowledge temperature most often climbs up. The degree to which topic knowledge temperature increases is consistent with topic’s average annual useful information increment in general. Especially in the two topics respectively led by ‘Bose-Einstein condensation in a gas of sodium atoms’ and ‘Particle swarm optimization’, the spike in average annual useful information corresponds with an upsurge in topic knowledge temperature (Fig 5(j) and 5(l)). While previous analysis on topic knowledge temperature unveals the significance of useful information growth to topic’s flourishing through popular child papers, this observation illustrates from a holistic view that useful information accumulation is primordial to topic’s prosperity.

We observe a rich variation in *T*^*t*^’s dynamics as each topic exhibits a unique development pattern. We identify 4 distinct topic life-cycles: rising topic, rise-then-fall topic, awakened topic and rise-and-fall-cycle topic (Fig 5). Rising topics demonstrate overall a steady and lasting *T*^*t*^ increase. They welcome rather intermittently their child papers that enjoy popularity within the topic. This ensures to some extent a stable knowledge increment. Rise-then-fall topics reach their peak at some point and then go downhill owing to the lack of new development of existing ideas, the absence of new study focus or the shadowing of their outstanding child papers. In addition, their expansion pace slows down during the cooling down phase. Awakened topics can have a mild development for a duration as long as 20 years before experiencing an influence surge. Their sudden flourishing is largely due to scientific communities’ recent frenzy in certain domains, such as artificial intelligence. Rise-and-fall-cycle topics manifest a more complicated *T*^*t*^ pattern. However, their rises and falls also match the global background, such as the introduction of the Internet, the booming of artificial intelligence and the prevalence of online social networks. Detailed result discussion for each topic can be found in S1 File Section S2.1-S2.4.

How is heat distributed within a topic? To answer this question, we interpreted *T*^*t*^ as average temperature and computed paper knowledge temperature for every article. Paper knowledge temperature gauges a work’s relative popularity and impact within the topic at a certain moment. We assumed that paper knowledge temperature is influenced by both the popularity of an article’s own idea, which is partly acquired from its references, and the overall popularity of the works that draw inspirations from it. We expect an article to be “hot” if it possesses trendy ideas itself and/or it inspires some popular articles. At each timestamp *t*, we assumed the hottest and coldest works and then employed the heat equation to propagate the heat across *G*^*t*^. For a node *u*, its temperature change $\frac{dT_{u}}{di}$ is (we omit the superscript *t* of paper knowledge temperature in the equation):

$$\begin{array}{c} \frac{dT_{u}}{di}=\sum_{v=1}^{|V^{t}|}\tilde{A_{vu}^{t}}\left(T_{v}-T_{u}\right) \end{array}$$ (3)

where $\tilde{A_{vu}^{t}}$ is the thermal conductivity between node *v* and node *u*. $\tilde{A_{vu}^{t}}$ depends on *DiffIdx*_*vu*_. The bigger *DiffIdx*_*vu*_ is, the bigger the difference between node *v* and node *u* and the faster their heat exchange. We set the pioneering article to be the hottest node (knowledge temperature = 1) and all the underdeveloped papers to be the coldest nodes (knowledge temperature = 0). We modelled heat propagation via idea inheritance and youngster’s contribution to knowledge renaissance respectively by forward and backward iterations of the heat equation. The number of iteration *i* depends on the average hops between 2 randomly selected nodes. Finally we performed a scaling by *T*^*t*^. *u*’s paper knowledge temperature at timestamp *t*, $T_{u}^{t}$ is therefore:

$$\begin{array}{c} T_{u}^{t}=T_{u,std}^{t}\cdot \frac{T^{t}}{\bar{T_{std}^{t}}} \end{array}$$ (4)

where $T_{u,std}^{t}$ is *u*’s temperature and $\bar{T_{std}^{t}}$ the average temperature derived from the heat equation.

We visualized paper knowledge temperature together with skeleton tree. If we let alone the coldest papers, we observe a ubiquitous phenomenon: the closer an article is to the pioneering work, the hotter it tends to be. Paper knowledge temperature decreases along paths in skeleton tree (Fig 4(c)–4(f)). This suggests that idea inheritance plays a main role in determining article’s popularity. Although pioneering work is the only known hottest node, we identify other heat sources, the majority of which are the centers of non-trivial clusters. Most heat sources happen to be among the most-cited child papers within a topic. They possess primarily intrinsic value. Their own research content contributes a lot to topic’s survival and flourishing. Another type of heat source are articles situated between clusters. Such papers may not have made astonishing discoveries nor have attracted many followers, but it is their studies that have inspired some influential subsequent work. Their value lies essentially in the enlightenment.

**Fig 6:**
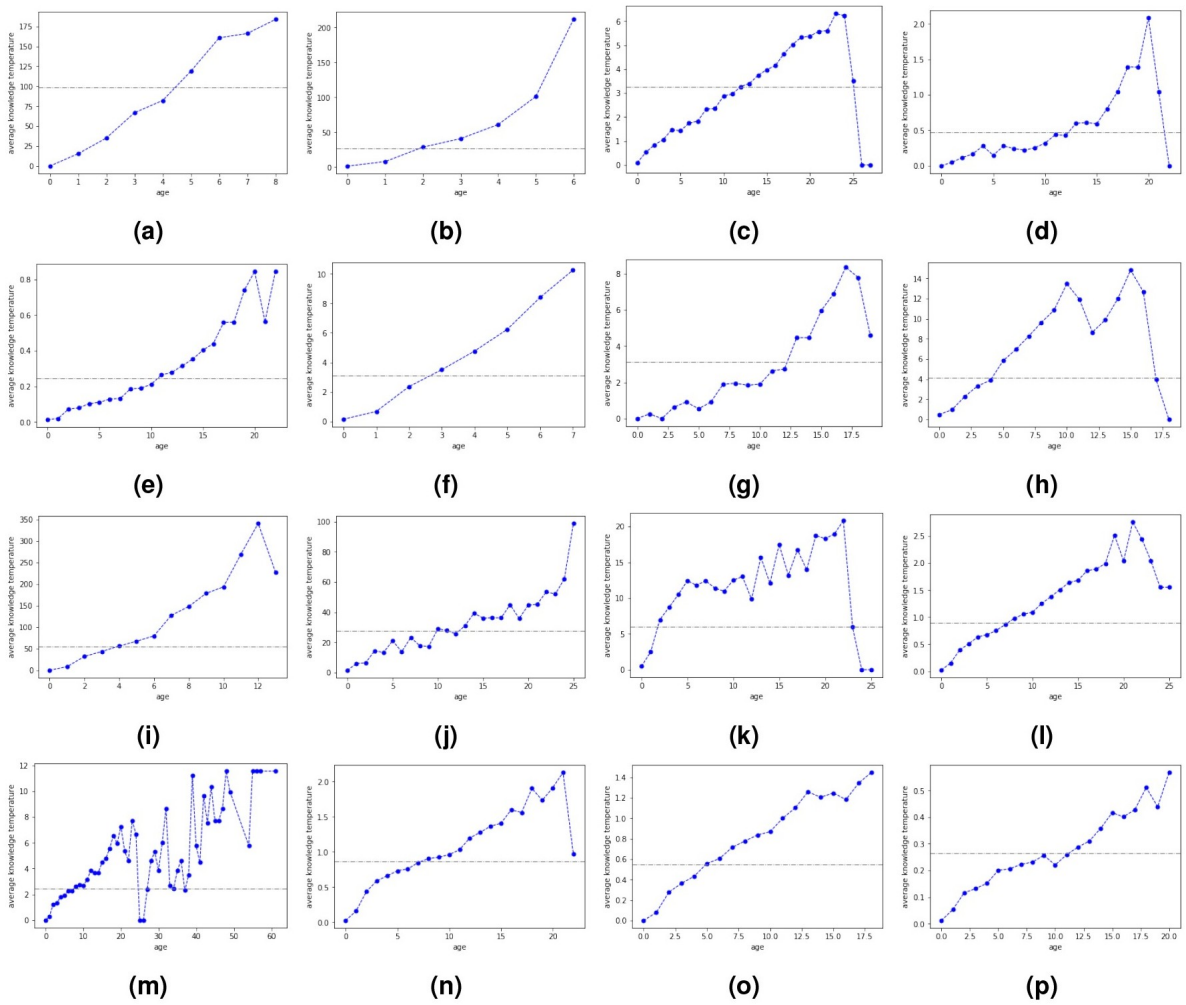
Relation between article age and paper knowledge temperature for 16 topics. Article age = 2020—year of publication. Grey dotted horizontal line marks the topic knowledge temperature (average level) in 2020. (a) Topic led by ‘Regulatory T Cells: Mechanisms of Differentiation and Function’. (b) Topic led by ‘Empirical Evaluation of Gated Recurrent Neural Networks on Sequence Modeling’. (c) Topic led by ‘Neural networks for pattern recognition’. (d) Topic led by ‘Critical Power for Asymptotic Connectivity in Wireless Networks’. (e) Topic led by ‘The capacity of wireless networks’. (f) Topic led by ‘Efficient Estimation of Word Representations in Vector Space’. (g) Topic led by ‘Coverage problems in wireless ad-hoc sensor networks’. (h) Topic led by ‘A neural probabilistic language model’. (i) Topic led by ‘A unified architecture for natural language processing: deep neural networks with multitask learning’. (j) Topic led by ‘Bose-Einstein condensation in a gas of sodium atoms’. (k) Topic led by ‘Long short-term memory’. (l) Topic led by ‘Particle swarm optimization’. (m) Topic led by ‘On random graphs, I’. (n) Topic led by ‘Collective dynamics of ‘small-world’ networks’. (o) Topic led by ‘Latent dirichlet allocation’. (p) Topic led by ‘A FUNDAMENTAL RELATION BETWEEN SUPERMASSIVE BLACK HOLES AND THEIR HOST GALAXIES’.

In an effort to better understand general heat distribution within a topic, our preliminary observation prompted us to study the relation between paper knowledge temperature and article age, as papers located in skeleton tree cores are parents or ancestors to papers on the periphery. We find that regardless of research themes, older articles indeed tend to have higher paper knowledge temperatures (Fig 6). Older papers take advantage of a longer time span and tend to better diffuse their ideas thanks to their numerous followers, a tendency in line with our intuition. Since we assume pioneering works possess the “hottest” knowledge, the gradual temperature decline well illustrates that idea inheritance and innovation are taking place simultaneously in every scientific topic. However, we observe a drop in average paper knowledge temperature among the oldest papers in half of the topics. 2 phenomena can explain the anomaly. Some topics contain a tiny fraction of atypical citations where younger articles are cited by older papers or papers published at approximately the same time. When the younger articles happen to be pioneering works, the oldest papers are no longer the topic founders. They usually have inspired few or even no child papers in the topics. Consequently, they are among the coldest articles. In rare cases, these papers inspired a certain quantity of works. But they remain “cold” owing to their relatively different research focus with that of the pioneering works even though they are connected to the latter. Their citations are more like peer bonds rather than a symbol of inspiration and idea inheritance. Such is the case for the pioneering work ‘Particle swarm optimization’ and its peer and popular child paper ‘A new optimizer using particle swarm theory’.

Even if we let alone the cold old articles, the heat distribution is not that simple and monotonous. We observe in most topics that parent papers are not always hotter than its descendants. According to our design, paper knowledge temperature is affected by 2 factors: the heat-level of its own research content and the promotion gained from its descendants. Therefore, a colder parent or ancestor is either due to its less prevalent ideas or a poor general performance of its children. This phenomenon implies that an important status within the topic does not necessarily bring much fame.

We further compared paper knowledge temperature with in-topic citation count, a traditional article-level impact metrics, to get a better understanding of their similarities and differences (S49 Fig in S1 File). We find a weak positive correlation between the two quantities among the best-cited papers in topics. In particular, we highlighted the most-cited child papers together with pioneering works on current skeleton trees. Most of them have a knowledge temperature above average as they are represented as yellow, orange or red nodes (current skeleton trees in S3, S9, S15, S35 Figs in S1 File for example). However, there are exception. For instance, in topic led by ‘Particle swarm optimization’, popular child paper ‘A new optimizer using particle swarm theory’ (NOPST) is among the coldest despite the fact that it is the most influential child paper in terms of citation count (S35 Fig in S1 File). NOPST was published in the same year as the pioneering work and it only cited the pioneering work. Its low temperature is due to its relatively different research focus with that of the pioneering work and an overall low heat level of its children. The latter is somehow also a consequence of the former, as the pioneering work has most prevalent idea. The focus difference is also reflected by their separation in the skeleton tree.

We also tracked the evolution of paper knowledge temperature of relatively popular child papers within a topic and we find a similar phenomenon already observed at topic-level. While an article’s own knowledge largely determines its heat level, child papers sometimes play a perceptible role in boosting or maintaining its popularity and impact. For example, in the topic led by paper ‘Bose-Einstein condensation in a gas of sodium atoms’, article ‘Bose-Einstein condensation of exciton polaritons’ has so far kept being hotter despite a global cooling since 2013 thanks to an above-average active development (S1 File Section S2.2.7). Our finding is consistent with the research which demonstrates that papers need new citations to keep their visibility [30]. Besides, in some topics, especially the one led by ‘Collective dynamics of ‘small-world’ networks’, we frequently find that popular child papers were published in renowned journals such as Nature and Science (S1 File Section S2.4.2). Our observation accords with research which suggests a positive association between journal prestige and article high impact [31].

Nonetheless, we find that several scientific topics are intimately connected. Some pioneering works occupy a primordial position in other topics’ skeleton trees. Furthermore, these closely related topics manifest similar knowledge temperature dynamics. However, such similarity does not correspond very well with idea inheritance and development in some cases. For instance, paper ‘The capacity of wireless networks’ (CMN) is the most successful child paper of the pioneering work ‘Critical Power for Asymptotic Connectivity in Wireless Networks’. It plays a crucial role in topic’s prosperity (S12 Fig in S1 File) by jointly inspiring one third of the topic members, most of which were published during the flourishing period. Besides, CMN surpassed and took over its predecessor to be the new authority in their domain in just a few years. Yet, according to their topic knowledge temperatures, it is the topic led by CMN that went downhill first. To this end, we wanted to design a mechanism that can better capture the interactions among closely-connected topics. We define topic group to be a set of closely-related topics where idea inheritance can be observed among the pioneering works. Following our skeleton tree notion, we were inspired by the nutrition transfer among real trees in a forest [32]. We hence treated scientific topics as trees and conceived a forest helping mechanism where thriving topics in a topic group transfuse a small fraction of vigor to their dying siblings. The amount of shared energy depends on both the ages and the size of the topic group. In knowledge temperature experiments for a topic group, topics first evolve individually and get their own knowledge temperature based on knowledge increase and structure growth. By the end of each timestamp, forest helping is performed, when applicable, to adjust topics’ knowledge temperature. When we compare topic knowledge temperatures before and after forest helping, we find that our helping mechanism regulates mildly the temperatures as if it took into account the “background popularity”, average popularity of a bigger research topic to which the group belongs. Overall, forest helping slightly reduces the fluctuation of *T*^*t*^ (S51 Fig in S1 File). Detailed forest helping experiment results can be found in S1 File Section S2.5.

Our work is different from existing literature on topic popularity in several aspects. To begin with, we defined topic based on an influential article, while other efforts in topic trend observation adopt keyword-based topic definition. Consequently, we used different types of data. We conducted our analysis over topic citation network, whereas relevant works required rich text information, at least full abstracts to embark on their analysis. Next, we modelled scientific popularity by borrowing ideas from thermodynamics. On the contrary, other literature adopted simple indicators such as publication-based trend indices [33], usage data [34] and keyword occurrence statistics [35] to depict the ‘heat’ of topics. Drastically different as it seems, both our method and relevent works’ analysis make use of publication number to derive topic’s popularity. Last but not least, knowledge temperature we proposed is able to track the rise and fall of scientific topics and to describe their lifecycles in a long term, while existing researches focus on the comparison between different topics at a given time and hence on the detection of hot trends.

## Conclusion

In summary, we report a thermodynamic approach to evaluate the popularity of scientific topics. We design knowledge temperature, an intuitive and quantitative metrics to characterize topic popularity dynamics by fully leveraging the scale and structure evolution of topic citation network through skeleton tree. Our analysis unveals that a continuous stream of useful information is the key to topics’ prosperity in the long run, to which the arrival of eminent child papers contributes a lot. In the short term, critical events such as the merge and emergence of new sub-topic also boost topic’s vigor. Based on knowledge temperature dynamics, we further unearth four distinct life-cycles of scientific topics. In addition, we dive into each topic and examine its ineer heat diffusion. We discover that older articles and articles that earn relatively high in-topic citation count generally have bigger chances to diffuse their ideas and thus tend to be hotter. However, exceptions are not uncommon, suggesting thus a weak positive correlation between heat-level and article’s age and in-topic citation number. Finally, we design a forest helping mechanism to better depict the idea inheritance and development in a topic group, a set of intimately-associated topics. Although knowledge temperature cannot directly be used as a scientific impact metrics, our study suggests a new possibility to quantify research impact in a most intuitive way.

## Supporting information

S1 File Detailed modelling description and experiment results. (PDF)
