# Supplementary material for: Can we ‘feel’ the temperature of knowledge? Modelling scientific popularity dynamics via thermodynamics
Source: PLoS One. 2021 Feb 11;16(2):e0244618. doi: 10.1371/journal.pone.0244618 (PMC7877646; doi:10.1371/journal.pone.0244618)
Supplement: S1 File — (PDF) [file pone.0244618.s001.pdf]

Can we 'feel' the temperature of knowledge? Modelling scientific popularity dynamics via thermodynamics

Luoyi Fu[1,◕], Dongrui Lu[1,◕], Qi Li[1], Xinbing Wang[1*], Chenghu Zhou[2*],

1 Electronic Engineering, Shanghai Jiao Tong University, Shanghai, China
2 Institute of Geographical Sciences and Natural Resources Research, Chinese Academy of Sciences, China

◕These authors contributed equally to this work.
* xwang8@sjtu.edu.cn, zhouch@lreis.ac.cn

Contents

S1 Model **2**
S1.1 Topic Skeleton Tree 2
S1.2 Structure Entropy 4
S1.3 Topic Knowledge Temperature 4
S1.3.1 T^t_{growth} 4
S1.3.2 $T^t_{structure}$ 6
S1.4 Paper Knowledge Temperature 10
S1.5 Forest Helping 10

S2 Experiments **11**
S2.1 Rising Topics 12
S2.1.1 Regulatory T Cells: Mechanisms of Differentiation and Function 12
S2.1.2 Empirical Evaluation of Gated Recurrent Neural Networks on Sequence Modeling 14
S2.1.3 Neural networks for pattern recognition 17
S2.2 Rise-then-fall Topics 23
S2.2.1 Critical Power for Asymptotic Connectivity in Wireless Networks 23
S2.2.2 The capacity of wireless networks 26
S2.2.3 Efficient Estimation of Word Representations in Vector Space . . 30
S2.2.4 Coverage problems in wireless ad-hoc sensor networks 32
S2.2.5 A neural probabilistic language model 37
S2.2.6 A unified architecture for natural language processing: deep neural networks with multitask learning 41
S2.2.7 Bose-Einstein condensation in a gas of sodium atoms 47
S2.3 Awakened topics 51
S2.3.1 Long short-term memory 51
S2.3.2 Particle swarm optimization 57
S2.4 Rise-fall-cycle topics 61
S2.4.1 On random graphs, I 61
S2.4.2 Collective dynamics of 'small-world' networks 66
S2.4.3 Latent dirichlet allocation 70
S2.4.4 A FUNDAMENTAL RELATION BETWEEN SUPERMASSIVE BLACK HOLES AND THEIR HOST GALAXIES 72
S2.5 Topic Group 80
S2.5.1 wireless network group 80

S2.5.2 RNN gated unit group 81
S2.5.3 word embedding group 81

S1 Model

The whole model is based on the assumption that topic's scientific popularity is influenced by knowledge quantity and knowledge strcture dynamics. If a topic succeeds in aggregating knowledge and manifests non-trivial changes in knowledge structure, then it should be a thriving research direction. To this end, we design 2 independent components for topic knowledge temperature, with one evaluating topic's vigor from knowledge quantity accumulation and the other from knowledge strcuture dynamics. Separately, for each component design, we treat topic citation network $G^t = (V^t, E^t)$ as a thermodynamic system and make an analogy between topic's evolution and some specfic state changes of the system. G^t is a directed graph whose nodes consist of a pioneering paper and all the articles that directly cites it and whose edges are the citations among them. Its adjacency matrix A^t is defined as:

$$A^t_{uv} = \begin{cases} 1 & u \text{ cites } v \\ 0 & \text{otherwise} \end{cases}$$

As topic knowledge temperature relies on some quantities defined in skeleton tree extraction and knowledge entropy computation, we would like to organise our model description in the following order: we present first the construction of skeleton tree, then we define structure entropy for every paper based on topic skeleton tree. Next, we unfold our topic knowledge temperature design and at last we elaborate on paper knowledge temperature. For the remainder of the section, we use 'root','node' and 'edge' when we present our design mathematically. We use 'pioneering work', 'paper' and 'citation' when we interpret our model from a practical point of view. In reality, 'root' symbolizes 'pioneering work', 'node' corresponds to 'paper' and 'edge' refers to 'citation'.

For the sake of a better notation distinction, we assign superscript t to variables that are related to topic citation network G^t and use subscript t to denote variables that are linked to thermodynamic systems.

S1.1 Topic Skeleton Tree

Skeleton tree illustrates the core knowledge structure of a topic. Its evolution reveals the key idea inheritance that takes place in the topic. The extraction of skeleton tree is essentially a process to reduce G^t to a tree structure, which we denote as $Tree^t = (V^t_T, E^t_T)$. There are altogether 3 steps in $Tree^t$'s construction:

1. We perform node embedding and compute distance matrix $EmbedDist$ that shows the node pair-wise distance in embedding space.

2. We derive matrix $DiffIdx$ based on $EmbedDist$ to measure the difference between every node pair. Vector $ReductionIdx$, a node score which serves to judge edge importance, is computed afterwards. We rely on $ReductionIdx$ to prune G^t in the following step.

3. We reduce G^t to $Tree^t$ by removing less important edges while ensuring the overall connectivity. The significance of an edge is determined by the similarity of its 2 extremities, which is assessed through their reduction indices. The process involves loop cutting and tree pruning. Every node except the root has at most one edge in $Tree^t$.

For notation simplicity, we omit superscript t for variables that are introduced in the rest of this subsection.

We start by slightly modifying adjacency matrix A by adding a self-loop to the root. This is for the convenience of spectral decomposition. Then, we compute out-degree matrix D and normalized Laplacian matrix $\widetilde{L} = D^{-\frac{1}{2}}(D-A)D^{-\frac{1}{2}}$. D is a diagonal matrix. Each entry on the diagonal equals a node's out-degree, or practically speaking a paper's in-topic citation count. We next perform a full spectral decomposition of $\widetilde{L}$. The eigenvectors are our node embeddings and $EmbedDist$ is a distance matrix with entry $EmbedDist_{u,v} = \parallel eigenvector_u - eigenvector_v \parallel_2$.

Now we proceed to compute difference matrix $DiffIdx$. For node pair (u, v), we define their difference index $DiffIdx_{u,v}$ as:

$$DiffIdx_{u,v} = \sum_{v_{parent}} d_{u,v_{parent}}$$

v_{parent}s are the predecessors of v and $d_{u,v_{parent}}$ is the shortest weighted path between u and v_{parent}:

$$d_{u,v_{parent}} = \begin{cases} \sum_{(i,j)\in path} EmbedDist_{i,j} & \text{if there exists a path between } u \text{ and } v_{parent} \\ MaxDist \times avgStep & \text{otherwise} \end{cases}$$

$MaxDist$ is the biggest distance between two connected nodes, $MaxDist = \max_{(a,b)\in E^t}(EmbedDist_{a,b}A_{a,b})$ and $avgStep$ is the average hop number of all shortest paths between any two reachable nodes. $DiffIdx$ gauges the difference between u and v by involving works that inspire v. If u and v_{parent} is reachable from each other, it suggests that there is some degree of similarity in their ideas or research topics and thus we represent their distance by shortest path's weight. Else, we model their correlation by a long imaginary path of $avgStep$ hops and step length of $MaxDist$. Therefore, the greater $DiffIdx_{u,v}$ is, the more different u and v are.

For a node u, its reduction index $ReductionIdx_u$ is defined as the sum of its difference indices:

$$ReductionIdx_u = \sum_{v\in V^t \setminus u} DiffIdx_{u,v}$$

Vector $ReductionIdx$ helps to determine edge importance. A citation between two articles with similar reduction indices is considered more valuable than one between two papers with different reduction indices.

We are now ready to extract topic skeleton tree. The first step is to find and cut loops in G^t. We cut a loop by removing the least important edge (its extremities have the most different reduction indices). In case there are multiple choices, randomly pick one and perform the removal. Nonetheless, we try to ensure that the edge we cut is not the last incoming edge left for some node so as to preserve overall connectivity as much as possible. After loop cutting, we obtain an intermediate loop-free graph. The second step is to remove redundant edges in this graph. Recall that we only keep one incoming edge for every node except the root in $Tree^t$. Fig. 2(a) illustrates the whole process with a toy example.

Ideally, $Tree^t$ has the following properties:

1. The pioneering work is the only root;

2. If we traverse $Tree^t$ starting from its root (in ascending order of publication year), we will reach every node in G^t.

S1.2 Structure Entropy

We adopt structure entropy[32] to determine the node size in the skeleton tree visualisation. Structure entropy measures the uncertainty of the tree structure if node u is absent. Consequently, it makes sense to evaluate the importance of a paper to knowledge passing within the topic by structure entropy. For a node u other than the root, its structure entropy S_u^t is defined as:

$$S_u^t = -\frac{g_{T,u}^t}{2|E_T^t|} \log \frac{V_{T,u}^t}{V_{T,u_{parent}}^t}$$

$g_{T,u}^t$ is the cut size of the sub-tree $Tree_u^t$ whose root is u. It is the sum of the degree of nodes in $Tree_u^t$ in $Tree^t$. E_T^t is the edge set of skeleton tree. $V_{T,u}^t$ is the number of nodes $Tree_u^t$ contains (the sum of out-degrees of $Tree_u^t$) and $V_{T,u_{parent}}^t$ the number of nodes $Tree_{u_{parent}}^t$ has.

The term before log measures the importance of $Tree_u^t$ to the whole skeleton tree and the log part describes the uncertainty of $Tree_u^t$ with respect to its parent sub-tree.

Structure entropy of the entire topic, S^t, is defined as the sum of node structure entropy:

$$S^t = \sum_{u \in Tree^t, u \neq root} S_u^t = -\sum_{u \in Tree^t, u \neq root} \frac{g_{T,u}^t}{2|E_T^t|} \log \frac{V_{T,u}^t}{V_{T,u_{parent}}^t}$$

S^t measures the quantity of information embedded in G^t's structure in a microscopic manner by examining the structure entropy of every node.

S1.3 Topic Knowledge Temperature

Topic knowledge temperature T^t is defined as:

$$T^t = T_{growth}^t + T_{structure}^t$$

where T_{growth}^t measures knowledge increment and $T_{structure}^t$ estimates the degree of latest structural changes in topic's knowledge framework.

S1.3.1 T_{growth}^t

T_{growth}^t aims to gauge G^t's knowledge increase by leveraging G^t's structure. To achieve this, we make an analogy between G^t and ideal gas. Furthermore, we make the following assumptions:

1. Topics do not experience much change in restriction pressure during its development. In other words, we assume topics are able to grow in a stable environment;
2. Topic size is constantly on the increase. We do not consider extreme cases such as publication withdrawal;
3. The knowledge a topic possesses is always valuable;
4. The more knowledge a topic has, the more likely it keeps its popularity.

We model the pressure of ideal gas to be the external restraints imposed on topic. Hence, due to assumption 1, the state changes of ideal gas take place at a constant pressure level.

Thermodynamic temperature is a derived function defined by energy and entropy. Yet in the case of ideal gas, there are other ways to compute temperature. Here, we find it more intuitive to define temperature from state variables including volume and mole number. We initialise T_{growth}^{t} by combining the 2 expressions of ideal gas's internal energy U:

$$U = cnT$$
$$U = ke^{\frac{S}{cn}} V^{-\frac{R}{c}} n^{\frac{R+c}{c}}$$

where S is entropy, n is substance amount (number of moles), V is volume, R is ideal gas constant, c is heat capacity and k adjusting coefficient.

As a result, T_{growth}^{0} writes:

$$T_{growth}^{0} = ke^{\frac{S_0}{cn_0}} \left(\frac{n_0}{V_0}\right)^{\frac{R}{c}}$$

where S_0 is the initial structure entropy of the topic , n_0 initial topic mole number, V_0 initial topic volume, k coefficient to be determined and R and c two constants.

Next, we update T_{growth}^{t} according to the ideal gas state equation $PV = nRT$(note that P is fixed):

$$T_{growth}^{t} = T_{growth}^{t-1} \frac{n_{t-1}}{n_t} \frac{V_t}{V_{t-1}}$$

The association between thermodynamic variables and G^t's variables is as follows:

1. Volume V_t: topic node number, $V_t = |V^t|$;
2. Mole number n_t: topic overlapped information, $n_t = |V^t| - UsefulInfo^t$;
3. Initial entropy S_0: topic structure entropy S^0, see previous subsection for details;

From the definition above, the internal energy U_t of ideal gas can be derived using mole number n_t and topic knowledge temperature T_t and the expression $U_t = cn_tT_t$. The entropy S_t of ideal gas during evolution can be derived iteratively using the expression $dS = nc_v\frac{T}{dT} + nR\frac{dV}{V}$ together with thermodynamic variables including volume V_t, topic knowledge temperature T_t and the entropy at the last time stamp S_{t-1}. If we make the hypothesis that c_v is constant during state change from (P,V_{t-1},n_{t-1}, T_{growth}^{t-1}) to (P,V_t,n_t, T_{growth}^{t}) and that $n_{t-1} \approx n_t$, we have $S_t = n_tc_v(\ln T_{growth}^{t} - \ln T_{growth}^{t-1}) + n_tR(\ln V_t - \ln V_{t-1}) + S_{t-1}$. Different from S_0, which microscopically quantifies topic's information hidden in topic's structure, S_t depicts this part of information from a macroscopic point of view with 2 state variables, temperature and volume.

$UsefulInfo^t$ is based on $DiffIdx$ in skeleton tree extraction:

$$UsefulInfo^t = \sum_{(u,v)\in Tree^t} \frac{DiffIdx_{u,v}}{\max_{(a,b)\in Tree^t} DiffIdx_{a,b}}$$

Nevertheless, we would like to finish this part with a qualitative analysis of T_{growth}^{t}'s dynamics from a macroscopic view of information and knowledge. Knowledge originates from information, but information and knowledge have different characteristics. Information is only valuable for one time. Duplicate information does not create any additional value, thus cannot be used to create knowledge. Knowledge is like an understanding and a refinement of information. It is always valuable. Normally speaking we cannot have too much knowledge.

Bearing the interplay of knowledge and information in mind, we are now ready to interpret the symbolic meaning of volume V_t and mole number n_t. V_t represents the

total amount of information possessed by a topic at timestamp t. $UsefulInfo$ signifies the amount of useful information and thus n_t symbolises the total amount of overlapped, or used information. We assume that each paper carries one unit of information. Yet we derive useful information edge by edge. This is because in a skeleton tree, all articles except the pioneering paper only have one citation, and if article u and its 'parent' ('child') article have drastically different $DiffIdx$s, they are likely to have distinct research contents. In this case, therefore, even if one of them has completely overlapped content with some other article(s), we can still roughly determine one unit of new information.

From the update rule of T^t_{growth}, we distinguish 3 cases:

1. T^t_{growth} will not change if V_t and n_t have identical increase rate during the last period.

2. T^t_{growth} will decrease if n_t increases faster than and V_t over the last period.

3. T^t_{growth} will increase if V_t increases faster than and n_t over the last period.

T^t_{growth} goes up when the quantity of total information grows faster than the amount of duplicate information. Note that $V_t - n_t = UsefulInfo^t$, T^t_{growth} rises when there is an accelerated increase in useful information. The more abundant useful information is, the bigger possibility for a topic to create new knowledge in the future and the greater potential a topic has. Otherwise, the topic "consumes" information faster than its information capital accumulation. If the tendency continues, it will have less information reserve for knowledge generation in the future. Its growth potential declines and eventually it 'dies'. Therefore, T^t_{growth} reflects both how smoothly the knowledge accumulation goes and how promising the topic is at timestamp t. As knowledge enrichment eventually brings about scientific impact, T^t_{growth} illustrates the long-term cumulative impact of a topic.

S1.3.2 $T^t_{structure}$

For $T^t_{structure}$ design, we make an analogy between G^t and a thermodynamic system that has freedom to vary its volume, temperature and pressure. For this system, the variation in internal energy dU is given by $dU = TdS - PdV + mdn$, where T is the temperature, P the pressure, dV the volume change, m the particle mass and dn the change in the number of particles[26]. The temperature T for an evolving network with fixed node number can be derived as $T = \frac{dU}{dS}$ [25]. It has been proved that with appropriate thermodynamic representations and some approximations, this relation is able to detect the critical events in a dynamic network[25,26].

Inspired by the above literature, we define $T^t_{structure}$ as:

$$T^t_{structure} = \left|\frac{dU_t}{dS_t}\right| = \left|\frac{U'_t - U_{t-1}}{S'_t - S_{t-1}}\right|$$

where S_{t-1}, S'_t are the von Neumann entropy of G^{t-1} and G'^t and U_{t-1}, U'_t the internal energy. G'^t is a weighted reduced graph of G^t induced by all the nodes in G^{t-1}. Apart from all the edges of G^{t-1}, G'^t also contains virtual links deduced from the nodes coming between timestamp $t-1$ and timestamp t.Intuitively, $T^t_{structure}$ can be interpreted as the magnitude of structural changes in G^{t-1}.

In the remainder of this part, we'd like first to elaborate on G'^t's construction and then to associate thermodynamic variables with topic's variables. Nonetheless, note that the above equation only applys for thermodynamic systems with fixed volume, we will precise which designs we have made in order to meet this premise.

The transformation from G^t to G'^t boils down to 2 tasks: remove new nodes and add virtual edges when possible. The weight of real edge is 1. For every new node x, we distinguish 2 cases:

- If x has only 1 parent node p_x, then remove x. If x has child node(s) c_x, connect it (them) to x's unique parent node and set the edge weight $A_{p_x c_x} = \frac{1}{2} A_{x c_x}$. Intuitively, since x only cites 1 paper, its arrival cannot give us extra information about whether any of the two articles in G^{t-1} that don't have a citation between them shares some of their research content.
- If x has multiple parent nodes, find all its "youngest" ancestor nodes in G^{t-1}. If a parent node p_x is in G^{t-1}, then p_x is already a "youngest" ancestor node. Else, iteratively find p_x's predecessors until they are in G^{t-1}. Note x's youngest ancestor nodes in G^{t-1} $(a_1, a_2, ..., a_m)$. Next, for each ancestor pair (a_i,a_j) between which there is no edge in G^{t-1}, add a directed virtual link according to their publishing year y_i, y_j (note A the real-time adjacency matrix, m the total number of x's youngest ancestor nodes):
  - If $y_i < y_j$, add a directed weighted edge from a_i to a_j of weight $\frac{2 \cdot \sum_{p_x} A_{p_x x}}{m(m-1)}$. The new edge means "$a_j$ virtually cites a_i".
  - If $y_i > y_j$, add a directed weighted edge from a_j to a_i of weight $\frac{2 \cdot \sum_{p_x} A_{p_x x}}{m(m-1)}$. The new edge means "$a_j$ virtually cites a_i".
  - If $y_i = y_j$, add a bidirectional weighted edge between a_i and a_j of weight $\frac{\sum_{p_x} A_{p_x x}}{m(m-1)}$. The new edge means "$a_j$, a_i virtually cites each other".

Fig. 2(b) illustrates a simple graph shrinking case.

In case of a duplicate virtual link, we discard it. In order words, we always keep the first virtual link added between a node pair. Remove x after adding all possible virtual links. Intuitively, since x cites several papers, we can guess that these papers are somehow loosely connected to one another even if there is no direct citations among them. That is why we add virtual citations of weight less than 1.

We set thermodynmaic volume V_t to be the node number of G^t:

$$V_t = |V^t| = n^t$$

As G^{t-1} and G'^t have identical node set, the assumption of $T^t_{structure}$ is satisfied.

For internal energy U_t's design, we adopt a statistical mechanical approach[25]:

$$U_t = \sum_{i=1}^{|V^t|} = p_{i,t} U_{i,t}$$

The probability that G^t occupies i's microstate is proportional to the eigenvalues $\tilde{\lambda}^t_j$ of normalized Laplacien matrix $\widetilde{L}^t$[25]:

$$p_{i,t} = \frac{\tilde{\lambda}^t_i}{\sum_{j=1}^{|V^t|} \tilde{\lambda}^t_j}$$

The element wise expression of $\widetilde{L}^t$ is:

$$\tilde{L}^t_{uv} = \begin{cases} 1 \quad \text{if } u = v \text{ and } \sum_u A^t_{uv} \neq 0 \\ -\frac{1}{\sqrt{d^t_u \cdot d^t_v}} \quad \text{if } u \neq v \text{ and } (u,v) \in E^t \\ 0 \quad \text{otherwise} \end{cases}$$

where d_u^t and d_v^t are the out-degrees of node u and v in G^t. We set miscrostate's internal energy, $U_{i,t}$ to be the out-degree of node i in G^t. By using the fact that the trace of an matrix is the sum of of its eigenvalues, we simplify U_t (recall that D^t denotes G^t's out-degree):

$$\begin{aligned} U_t &= \sum_{i=1}^{|V^t|} p_{i,t} U_{i,t} \\ &= \sum_{i=1}^{|V^t|} \frac{\widetilde{\lambda}_i^t}{|V^t|} d_i^t \\ &= \frac{1}{|V^t|} Tr(D^t \widetilde{L}^t) \\ &= \frac{1}{|V^t|} Tr(D^{t\frac{1}{2}} \widetilde{L}^t D^{t\frac{1}{2}}) \\ &= \frac{1}{|V^t|} Tr(L^t) \\ &= \frac{|E^t|}{|V^t|} \end{aligned}$$

For weighted graph ${G'}^t$, its internal energy is:

$$\begin{aligned} U_t' &= \frac{\sum_{(u,v)\in E'^t} {A'}_{uv}^t}{|V'^t|} \\ &= \frac{\sum_{(u,v)\in E'^t} {A'}_{uv}^t}{|V^{t-1}|} \end{aligned}$$

We approximate S_t and S_t' by node degree in G^t and ${G'}^t$ respectively. The von Neumann entropy for a directed graph is the sum of the von Neumann entropy of its strongly connected (SC) components[27]:

$$S = \sum_{SC} S_{SC}$$

Now assume the strong connectivity and we extend the entropy computation for unweighted directed graph[25,27] to that for a weighted directed graph $G = (V, E)$. For the clarity of First define some notations:

Bidirectional edge set E_{bd}:

$$E_{bd} = \{(u,v)|(u,v) \in E \,\text{and}\, (v,u) \in E\}$$

Adjacency matrix A:

$$A_{uv} = \begin{cases} w_{uv} & \text{if}(u,v) \in E \\ 0 & \text{otherwise} \end{cases}$$

In-degree and out-degree of node u:

$$d_u^{in} = \sum_{v\in V} A_{vu} \qquad d_u^{out} = \sum_{v \in V} A_{uv}$$

Transition matrix P:

$$P_{uv} = \begin{cases} \frac{A_{uv}}{d_u^{out}} & \text{if}(u,v) \in E \\ 0 & \text{otherwise} \end{cases}$$

Normalized Laplacian matrix $\tilde{L}$:

$$\tilde{L} = \begin{cases} 1 & u = v, d_v^{out} \neq 0 \\ -\frac{A_{uv}}{\sqrt{d_u^{out}}\sqrt{d_v^{out}}} & u \neq v, (u,v) \in E \\ 0 & \text{otherwise} \end{cases}$$

We note $\tilde{\lambda_s}$ normalized Laplacian eigenvalue and ϕ unique left eigenvector of transition matrix P.

The von Neumann entropy of G is the Shannon entropy associated with the normalized Laplacian eigenvalues.By adopting the quadratic approximation to the Shannon entropy (i.e. $-x \ln x \approx x(1-x)$), we have[27]

$$\begin{aligned} S &= -\Sigma_{s=1}^{|V|} \frac{\tilde{\lambda_s}}{|V|} \ln \frac{\tilde{\lambda_s}}{|V|} \\ &= \Sigma_{s=1}^{|V|} \frac{\tilde{\lambda_s}}{|V|}(1 - \frac{\tilde{\lambda_s}}{|V|}) \\ &= \frac{tr(\tilde{L})}{|V|} - \frac{tr(\tilde{L}^2)}{|V|^2} \\ &= 1 - \frac{tr(\tilde{L}^2)}{|V|^2} \end{aligned}$$

Now we expand the equation $tr(\tilde{L}^2) = |V| + \frac{1}{2}(tr(P^2) + tr(P\Phi^{-1}P^T\Phi))$ [27]for G:

$$tr(P^2) = \Sigma_{u\in V}\Sigma_{v\in V} P_{uv}P_{vu} = \Sigma_{(u,v)\in E_{bd}} \frac{A_{uv}A_{vu}}{d_u^{out}d_v^{out}}$$

$$\begin{aligned} tr(P\Phi^{-1}P^T\Phi) &= \Sigma_{u\in V}\Sigma_{v\in V} {P_{uv}}^2 \frac{\phi(u)}{\phi(v)} \\ &= \Sigma_{(u,v)\in E} \frac{\phi(u)}{\phi(v)} \cdot \frac{{A_{uv}}^2}{d_u^{out}} \end{aligned}$$

Combine the simplifications together and we have an approximation of G's entropy:

$$S = 1 - \frac{1}{|V|} - \frac{1}{2|V|^2}\left(\Sigma_{(u,v)\in E_{bd}} \frac{A_{uv}A_{vu}}{d_u^{out}d_v^{out}} + \Sigma_{(u,v)\in E} \frac{\phi(u)}{\phi(v)} \cdot \frac{{A_{uv}}^2}{d_u^{out}}\right)$$

By incorporating the approximation $\frac{\phi(u)}{\phi(v)} \approx \frac{d_u^{in}}{d_v^{in}}$[27], G's entropy can be written in terms of node degree:

$$S = 1 - \frac{1}{|V|} - \frac{1}{2|V|^2}\left(\Sigma_{(u,v)\in E_{bd}} \frac{A_{uv}A_{vu}}{d_u^{out}d_v^{out}} + \Sigma_{(u,v)\in E} \frac{d_u^{in}}{d_v^{in}} \cdot \frac{{A_{uv}}^2}{d_u^{out}}\right)$$

Finally, we obtain S_t and S_t':

$$S_t = \sum_{SC} S_{SC,t} = \sum_{SC} 1 - \frac{1}{|V_{SC}|} - \frac{1}{2|V_{SC}|^2}\left(\Sigma_{(u,v)\in E_{SC,bd}} \frac{1}{d_u^{out}d_v^{out}} + \Sigma_{(u,v)\in E} \frac{1}{d_u^{out}} \cdot \frac{d_u^{in}}{d_v^{in}}\right)$$

$$\begin{aligned} S'^t &= \sum_{SC} {S'}_{SC,t} \\ &= \sum_{SC} 1 - \frac{1}{|{V'}_{SC}|} - \frac{1}{2|{V'}_{SC}|^2}\left(\Sigma_{(u,v)\in {E'}_{SC,bd}} \frac{{A'}_{SC uv}{A'}_{SC vu}}{d_u^{out}d_v^{out}} + \Sigma_{(u,v)\in E} \frac{d_u^{in}}{d_v^{in}} \cdot \frac{{{A'}_{SC uv}}^2}{d_u^{out}}\right) \end{aligned}$$

S1.4 Paper Knowledge Temperature

Topic knowledge temperature depicts topic's overall popularity and impact. Meanwhile, we are also interested in how popularity is distributed within a topic. To this end, we design knowledge temperature for every paper in a topic, which we refer to as paper knowledge temperature. We employ the heat equation to compute paper knowledge temperature. For a node u, its temperature change $\frac{dT_u^t}{dt}$ is:

$$\frac{dT_u}{dt} = \sum_{i=1}^{|V^t|} \widetilde{A_{iu}^t}(T_i - T_u)$$

where $\widetilde{A_{iu}^t}$ is defined as:

$$\widetilde{A_{iu}^t} = A_{iu}^t \cdot \left(0.5 + \frac{DiffIdx_{i,u} - \min_{(a,b)\in E^t} DiffIdx_{a,b}}{\max_{(a,b)\in E^t} DiffIdx_{a,b} - \min_{(a,b)\in E^t} DiffIdx_{a,b}}\right)$$

$DiffIdx$ is defined previously in subsection topic skeleton tree. $\widetilde{A_{iu}}$ is the thermal conductivity between node i and node u.

Before the heat diffusion, we need to fix the temperature of certain nodes and to precise the number of iteration of the heat equation. We assume that the pioneering work is the hottest and all the inactive papers are the coldest. An article u is considered inactive if either of the following criteria is met:

1. u does not have any citation until timestamp t
2. If u joins in the topic before timestamp $t-1$ and u does not have any new citations between timestamp $t-1$ and timestamp t.

We first diffuse heat backward by transposing the adjacency matrix $\widetilde{A}$ for 1 iteration, then forward for $\lfloor avgStep \rfloor$ iterations. $avgStep$, defined during skeleton tree extraction, can be interpreted as the average hops between 2 random nodes in G^t. Backward propagation models the popularity gain in idea thanks to the newcomers and forward propagation models the heat diffusion due to the inheritance of topic knowledge.

Every node has a temperature between 0 and 1 after the heat diffusion. The last step is to scale this temperature by topic knowledge temperature. Denote $T_{u,std}^t$ to be node u's temperature and $\overline{T_{std}^t}$ the average node temperature right after the heat diffusion before the scaling. Thus, u's paper knowledge temperature, T_u^t is:

$$T_u^t = T_{u,std}^t \cdot \frac{T^t}{\overline{T_{std}^t}}$$

S1.5 Forest Helping

Forest Helping is designed for topic group. A topic group is a set of topics that have similar research interests. For example, as the 2 pioneering works 'Long short-term memory' and 'Empirical Evaluation of Gated Recurrent Neural Networks on Sequence Modeling' are both dedicated to gated unit design in recurrent neural network, we consider the topics led by them to be a topic group. Through forest helping, thriving topics within the group "transfuse" a small part of their energy to the other stagnant sister topics. Here, we make an analogy between each topic and ideal gas and we assume that forest helping will not change the total internal energy of a topic group:

$$\sum_{j=1}^{K} cn_j^t T_j^t = \sum_{j=1}^{K} cn_j^t T_{j,forest}^t$$

where K is the number of topics in a group, n_j^t is the mole number of topic j at timestamp t, T_j^t is topic j's knowledge temperature before forest helping at timestamp t and $T_{j,forest}^t$ is the topic knowledge temperature of topic j after the helping. Note that here, we adopt the same association between thermodynamic variables and topic citation network's variables as in T_{growth}^t's conception.

Within a topic group, a topic is either considered as 'thriving' or 'dying'. A topic is deemed as 'thriving' if it becomes hotter during the last observation period. Otherwise, it is classified as 'dying'. From timestamp $t-1$ to t, we first compute topic knowledge temperature and paper knowledge temperature separately for each topic in a topic group. Then, we perform forest helping within a topic group. If all topics in the group have a rising knowledge temperature, no helping takes place. Else, all of the topics with a rising knowledge temperature help the rest.

We model the probability that "Within a topic group, a thriving topic is willing to help others" follows a beta distribution $B(1, \sum_{j=1}^{K} a_j)$, a_j being topic age. Beta distribution varies from 0 to 1, which corresponds with option "not help" and option "help with all I have". We assume a prosperous topic will give an amount of energy equal to the expectation of the distribution. Hence, at time t, the energy that a topic gives away is proportional to its own knowledge temperature and is inversely proportional to the sum of ages of the entire group:

$$\Delta E = cn^t \frac{1}{1 + \sum_{j=1}^{K} a_j^t} T^t$$

The energy received by each topic in need of help is proportional to its number of moles. Therefore, they have an identical increase in their knowledge temperatures:

$$\delta T = \frac{\Delta E}{\sum_j n_j^t}$$

As topics mature, their initially close connection in thoughts will wear off by time. Consequently, the amount of energy transmitted through forest helping will decrease.

S2 Experiments

We first present our results and analysis for individual topic, next discuss the forest helping results for topic group. Note that most of the data for 2020 only cover the first 2 months, therefore the latest temperature is not definite. The data in the tables are rounded to 3 decimal places. We set two constants in T_{growth}^t's calculation as $R = 8, c = 1$. For topics with more than 5000 articles, the coefficient $k = 10$ in T_{growth}^0's computation. Else, $k = 100$.

In this section, we refer to "popular child papers" as the papers with high in-topic citations (excluding the pioneering work) unless explicitly specified. In expressions such as 'direct children of paper A', the 'children' refers to the articles that directly cite paper A. In expressions such as 'article A is the parent paper of article B', we mean the fact that 'article B directly cites article A'. When presenting the results of individual topic's knowledge temperature dynamics, especially how the dynamics corresponds with the actual topic development, we leverage both the evolution of topic skeleton tree and some well-known, widely recognized background knowledge.

Based on the evolution of knowledge temperature, we classify topics into 4 categories: rising topic, rise-and-fall topic, awakened topic and rise-fall-cycle topic. Among 16 topics, 9 follow a rise-then-fall pattern, with their knowledge temperature reaching record high shortly after birth. 3 topics have been almost always on the rise until today. 2 topics have waited a long time before being recognised and having a surge

in knowledge temperature. We refer to them as awakened topics. The rest exhibit a periodic knowledge temperature variation characterised by multiple up-down cycles.

S2.1 Rising Topics

S2.1.1 Regulatory T Cells: Mechanisms of Differentiation and Function

The topic has been thriving ever since its birth in 2012 (Fig. S1). It has a very stable annual growth of T^t and T^t_{growth}, which corresponds with its seemingly uniform publishing rhythm: an annual publication count always over 10% of the total size between 2013 and 2019. In addition, popular child papers came at a steady speed during 2012 and 2015. They have contributed to a steady inflow of useful information and helped maintain a stable knowledge accumulation (Fig. 5(a)).

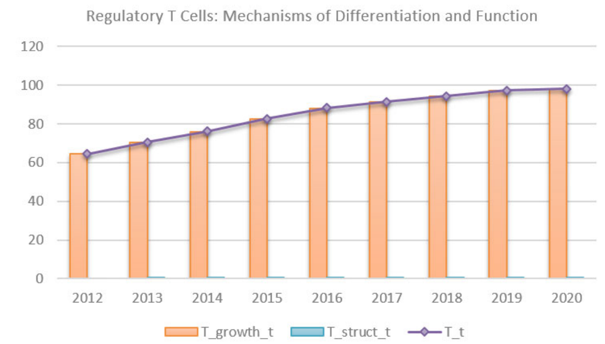

year	$\|V^t\|$	$\|E^t\|$	n_t	V_t	$UsefulInfo^t$	T^t_{growth}	T^t_{struct}	T^t
2012	49	58	46.15	49	2.85	64.466		64.466
2013	241	346	207.527	241	33.473	70.51	0.058	70.567
2014	460	773	367.669	460	92.331	75.964	0.09	76.053
2015	659	1321	484.941	659	174.059	82.509	0.046	82.556
2016	841	1949	579.149	841	261.851	88.168	0.043	88.211
2017	1027	2633	682.316	1027	344.684	91.388	0.022	91.411
2018	1199	3334	771.581	1199	427.419	94.35	0.024	94.375
2019	1356	4053	845.96	1356	510.04	97.323	0.016	97.339
2020	1381	4190	854.519	1381	526.481	98.125	0.004	98.129

Fig S1. Regulatory T cells: topic statistics and knowledge temperature evolution

$T^t_{structure}$ remains tiny, suggesting that this topic has a gradual knowledge structure progression and has not experienced a sudden short-term impact gain. Indeed, although we observe constant visible development in skeleton tree, we don't see any disruptive changes in the overall structure (Fig. S2). Under the leadership of several popular child papers, the topic have succeeded in developing some sub-directions, as is reflected by the fact that multiple non-trivial branches have been gradually growing out of the central cluster led by the pioneering work. Yet so far the pioneering paper remains the absolute topic center. Moreover, tiny twigs are forming around the center at a seemingly uniform speed, which may be a good sign for more novel research focus. The vigor of skeleton tree shows again the topic's slowly yet firmly rising popularity and impact.

Now we closely examine its latest skeleton tree (Fig. S3 bottom left). Almost all the hottest articles surround the pioneering paper and paper knowledge temperature decreases globally as the articles are located farther away from the pioneering paper. Note that the blue nodes that surround the pioneering work are articles with little development within the topic. If we let alone these coldest papers, the heat distribution fits the general rules ”the older the hotter” (Fig. 6(a)) and ”the more influential the

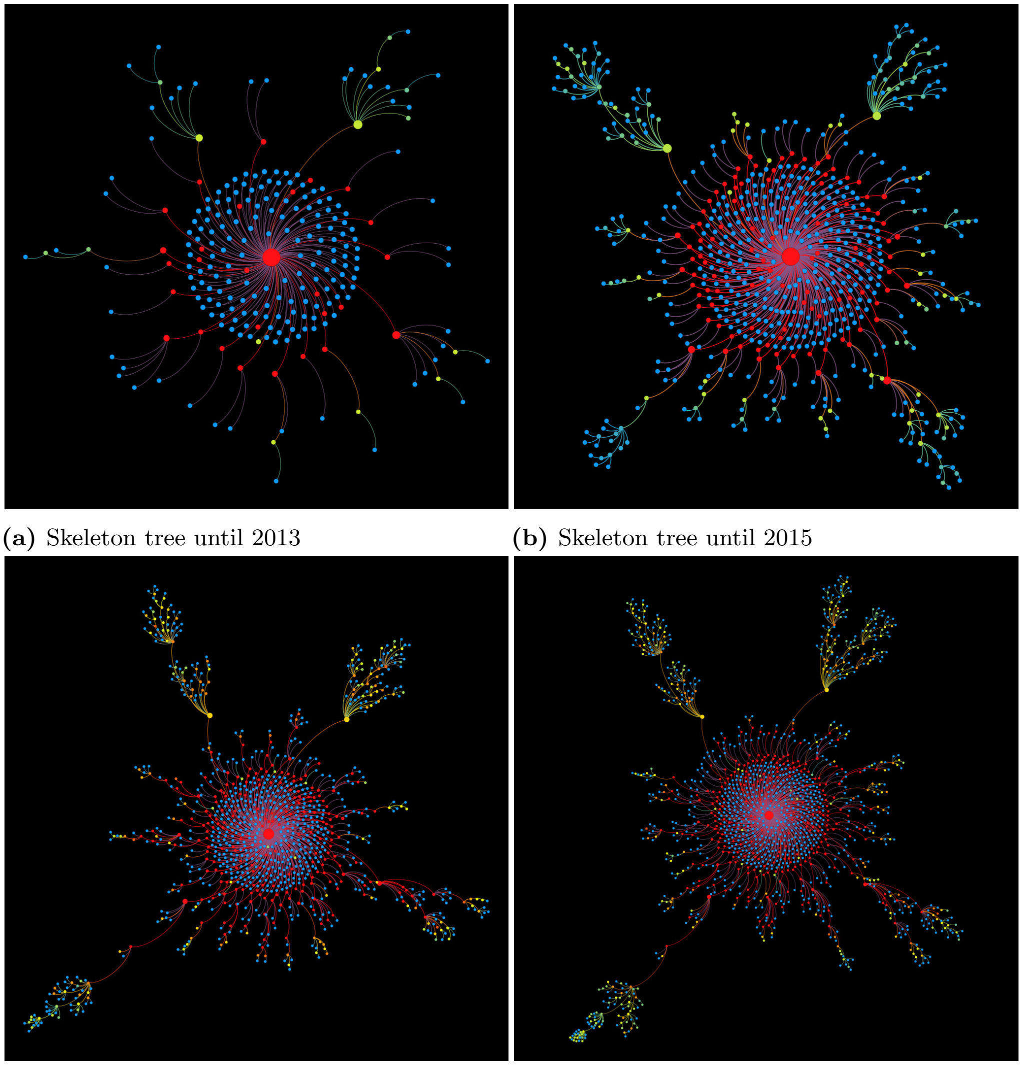

(a) Skeleton tree until 2013

(b) Skeleton tree until 2015

(c) Skeleton tree until 2017

(d) Skeleton tree until 2019

Fig S2. Regular T Cells: Skeleton tree evolution

hotter" (Fig. S63(a))). Nonetheless, there are exceptions. Age and citations are not guarantee for heat-level. For example, popular child paper 'Transcription factor Foxp3 and its protein partners form a complex regulatory network' is colder than some of its child papers in the research branch it leads (Fig. S3 bottom right). The intrinsic difference of their research ideas, which is partly reflected by the average heat-level of their citations, causes the temperature difference. Besides, we also identify some young and hot articles. For example, 2 papers published in 2017, 'TNFR2: A Novel Target for Cancer Immunotherapy' (TNFR2) and 'Crosstalk between Regulatory T Cells and Tumor-Associated Dendritic Cells Negates Anti-tumor Immunity in Pancreatic Cancer' and 1 paper published in Nature Immunology in 2018, 'c-Maf controls immune responses by regulating disease-specific gene networks and repressing IL-2 in CD4 + T cells' all have a knowledge temperature above average. All of them have already inspired several works. Their popularity not only manifests the boosting effect of new articles on original work, but also shows the lasting activity of this topic. Overall, these atypical examples suggest that the positive correlation between paper knowledge temperature and age or pure impact in terms of citation statistics is weak.

In particular, we find the knowledge temperature evolution of paper 'Basic principles of tumor-associated regulatory T cell biology' (BPTRT), published in 2013 in journal *Trends in Immunology* very interesting. This article is the parent paper of 'TNFR2: A Novel Target for Cancer Immunotherapy' in 2020's skeleton tree. Its temperature dropped from 213.26 to around 170 between 2013 to 2016 despite the fact that it had new followers and that the whole topic went hotter during this period. By the end of the following year, its temperature skyrocketed to around 330. The sudden gain is the result of an accumulated influence during period 2013-2016 and the global heat diffusion owing to the topic's gradual development. Its temperature has mildly climbed up since 2016, which is in accordance with topic knowledge temperature dynamics. The arrival of its promising child, TNFR2. TNFR2 has helped keep BPTRT's heat-level with its own development. This example well illustrates child article's role in maintaining parent paper's popularity and impact.

We observe in addition certain clustering effect in the skeleton tree. For example, almost all direct children of paper 'Pregnancy imprints regulatory memory that sustains anergy to fetal antigen' have similar research themes as itself (Table S1). This confirms the effectiveness of our skeleton tree extraction algorithm.

When comparing T^t's evolution with the keyword frequency of the pioneering work queried from Baidu Xueshu, we find topic knowledge temperature bears similarity with the keyword occurence trend until 2016. 'regular t-cell' is "hottest" during 2012 and 2014 and 'immune homeostasis' is "hottest" during 2013 and 2015 (Fig. S4). Indeed, we observe an accelerated increase in useful information accumulation in the early years, from 2012 to 2015 (Fig. 5(a)). Over the same period, T^t also witnesses a faster growth. (Fig. S1).

S2.1.2 Empirical Evaluation of Gated Recurrent Neural Networks on Sequence Modeling

As is shown by the basic statistics and T^t, the topic is keeping popularity and steadily gaining impact (Fig.S5). Its popular child papers came at a steady speed during 2015 and 2017. Apart from enriching topic knowledge pool with their own ideas, they also attracted new researches' attention and thus have helped maintain a stable knowledge accumulation (Fig. 5(b)). The topic has been accelerating its expansion since 2017. It witnessed the biggest annual publication count in 2019. Yet as most child papers published no earlier than 2018 have had little development, the publication surge did not result in a significant uprise in T^t.

$T^t_{structure}$ remains tiny compared to T^t_{growth}, suggesting that the topic has a gradual

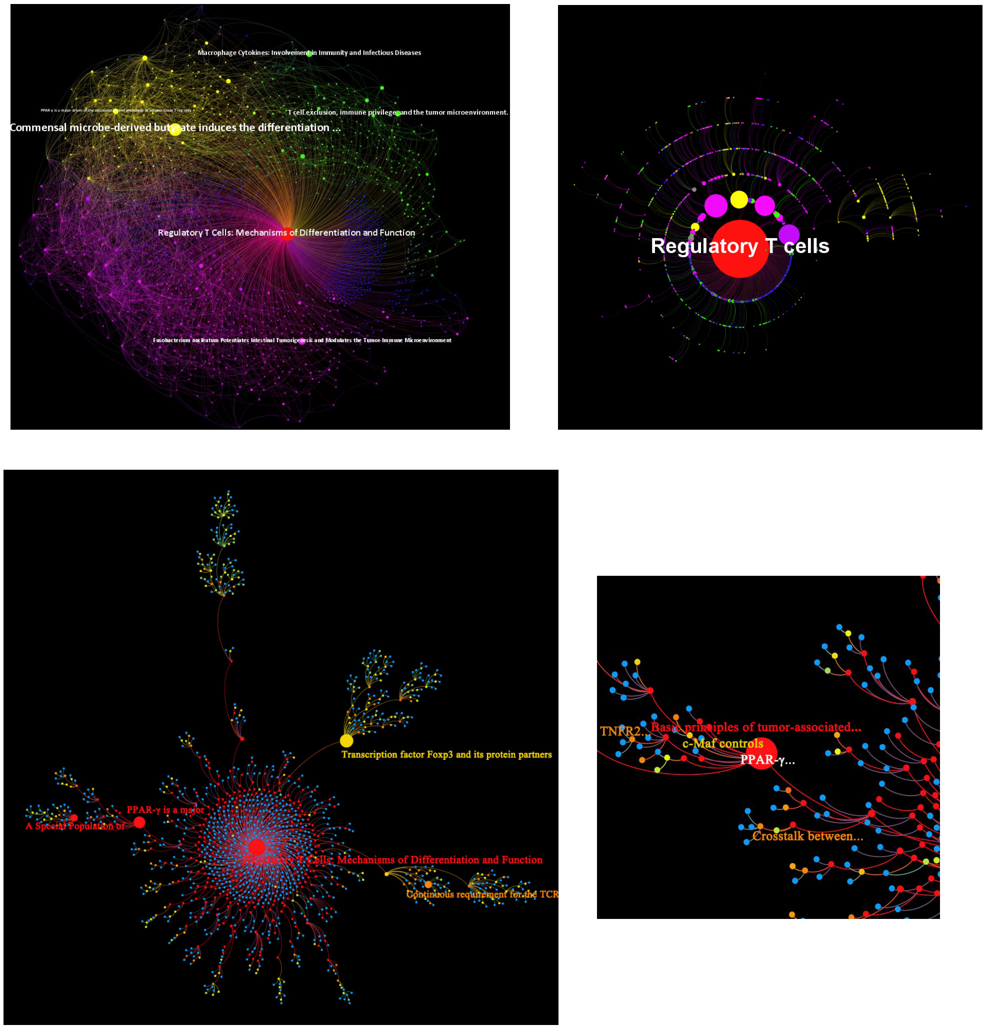

Fig S3. Regular T Cells: Galaxy map, current skeleton tree and its regional zoom. Top left: current galaxy map. Papers with the most total citations are labelled by their titles. Top right: current skeleton tree in circular layout, node color is the same as in galaxy map. Bottom left: current skeleton tree in ForceAtlas layout, node color is determined by paper knowledge temperature, with blue the coldest and red the hottest. Papers with more than 55 in-topic citations are labelled by title. Except the pioneering work, labelled nodes' size is amplified by 3 times. Bottom right: regional zoom of current skeleton tree in ForceAtlas layout.

title	year
Pregnancy imprints regulatory memory that sustains anergy to fetal antigen predictions using deep neural networks	2012
Mechanisms of T cell tolerance towards the allogeneic fetus	2013
Pregnancy Complications and Unlocking the Enigma of Fetal Tolerance Regulatory T Cells: New Keys for Further	2014
Regulatory T Cells: New Keys for Further Unlocking the Enigma of Fetal Tolerance and Pregnancy Complications	2014
The immunology of pregnancy: regulatory T cells control maternal immune tolerance toward the fetus	2014
Regulatory T Cells: Types, Generation and Function	2014
Daughter's Tolerance of Mom Matters in Mate Choice	2015
Regulatory T cells in embryo implantation and the immune response to pregnancy	2018
Alloreactive fetal T cells promote uterine contractility in preterm labor via IFN-γ and TNF $-\ \alpha$	2018

Table S1. Regular T Cells: Clustering effect example. First line is the parent paper and the rest children.

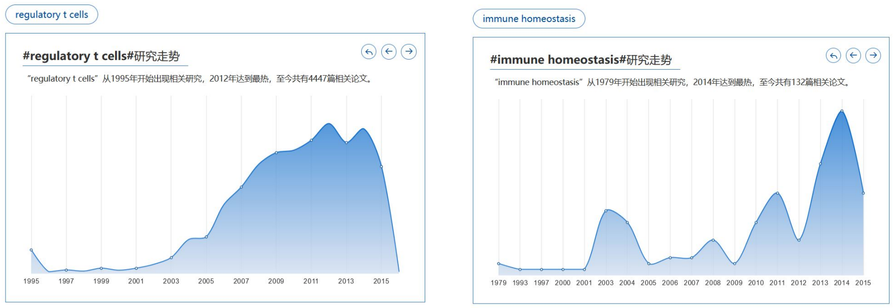

Fig S4. Regular T Cells: Popularity trend of pioneering work's keywords provided by baidu research engine.

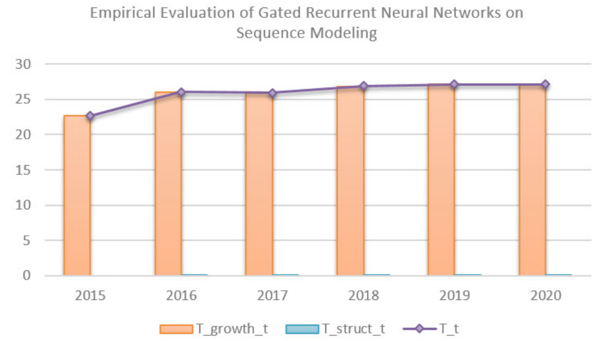

year	$\lvert V^t \rvert$	$\lvert E^t \rvert$	n_t	V_t	$UsefulInfo^t$	T^t_{growth}	T^t_{struct}	T^t
2015	53	76	43.823	53	9.178	22.686		22.686
2016	295	514	212.885	295	82.115	25.994	0.03	26.024
2017	749	1377	543.23	749	205.77	25.864	0.058	25.921
2018	1328	2619	929.445	1328	398.555	26.802	0.085	26.887
2019	2109	4287	1459.035	2109	649.965	27.115	0.034	27.149
2020	2282	4675	1576.618	2282	705.382	27.151	0.011	27.162

Fig S5. GRU: topic statistics and knowledge temperature evolution

knowledge structure progression and has not experienced a sudden short-term impact gain. Indeed, although its skeleton tree has constant visible development (Fig. S6), so far no child paper is able to defy the absolute authority of the pioneering paper, the center of the biggest cluster. Several popular child papers have each led a research sub-field in the topic, as is depicted by the small bundles extending from the central cluster. In particular, popular child paper 'LSTM: A Search Space Odyssey' in 2017 has inspired 2 schools of thoughts. The maturation of these newly emerged research directions accounts for a higher $T^t_{structure}$ in the first years of the topic. Overall, we observe a universal non-trivial growth in the skeleton tree. The vigor of skeleton tree shows again the slowly yet firmly increasing popularity and impact of this topic.

Now we closely examine its latest skeleton tree (Fig. S7). The decrease in paper knowledge temperature from the pioneering work, to the topic's periphery is obvious, which accords with the general rule "the older the hotter" (Fig. 6(b)). Note that in the current skeleton tree ((Fig. S7) bottom), the blue nodes that surround the pioneering work and popular child papers are articles with little development within the topic. In particular, the heat distribution is rather concentrated in old papers. This phenomenon is in line with our above observation that young child papers have little authority in the topic. The limited heat diffusion is also why most popular child papers have a paper knowledge temperature no greater than average. This topic is quite young. It needs more time to fully explore the potential of new ideas and to trigger a thorough heat diffusion in its range.

In particular, we find the knowledge temperature evolution of the second most-cited paper 'An Empirical Exploration of Recurrent Network Architectures', published in 2015 in journal *International Conference on Machine Learning* very interesting (Fig. S7 bottom). This article became much hotter from 2015 to 2016 thanks to its numerous child papers. However, its temperature reduces by half from 182.578 to 89.19 the next year upon the arrival of the third most-cited paper 'LSTM: A Search Space Odyssey', the leader of the right major branch in the skeleton tree (Fig. S6 (b,c)). Since then, its temperature has been slightly decreasing to around 80 in 2020. The sudden drop is a vivid illustration of the rivalry within the topic.

We observe in addition certain clustering effect in the skeleton tree. For example, almost all direct children of paper 'Machine Health Monitoring Using Local Feature-Based Gated Recurrent Unit Networks' study the industrial applications of gated recurrent unit network (Table S2). This illustrates the effectiveness of our skeleton tree extraction algorithm.

S2.1.3 Neural networks for pattern recognition

The topic gained popularity and impact steadily in its first 10 years, as is shown by its increasing size, T^t and a steady speed-up in useful information accumulation (Fig. S8, 5(c)). During this period, influential child papers within the topic, namely 'Pattern Recognition and Neural Networks' (PRNN) published in 1996 and 'A Tutorial on Support Vector Machines for Pattern Recognition' (SVMPR) published in 1998, shaped the skeleton tree altogether with the pioneering work. Their enrichment to topic knowledge structure accounts for a slightly higher $T^t_{structure}$ back then, which is manifested by the formation of 2 clusters in the skeleton tree (Fig. S9). Yet the pioneering work is still the absolute authority in the topic. In particular, the cluster in the top is led by PRNN and the top-left small cluster surrounds SVMPR (Fig. S10). Meanwhile, their arrival pushed up the T^t_{growth} as they also enlarged knowledge base together with common descendants with the pioneering work. Afterwards, despite a constant increase in total size, topic's T^t increment has slowed down. The popular child papers coming after 2000, namely 'Boosting the differences: A fast Bayesian classifier neural network' published in 2000 ,'A tutorial on support vector regression' published in

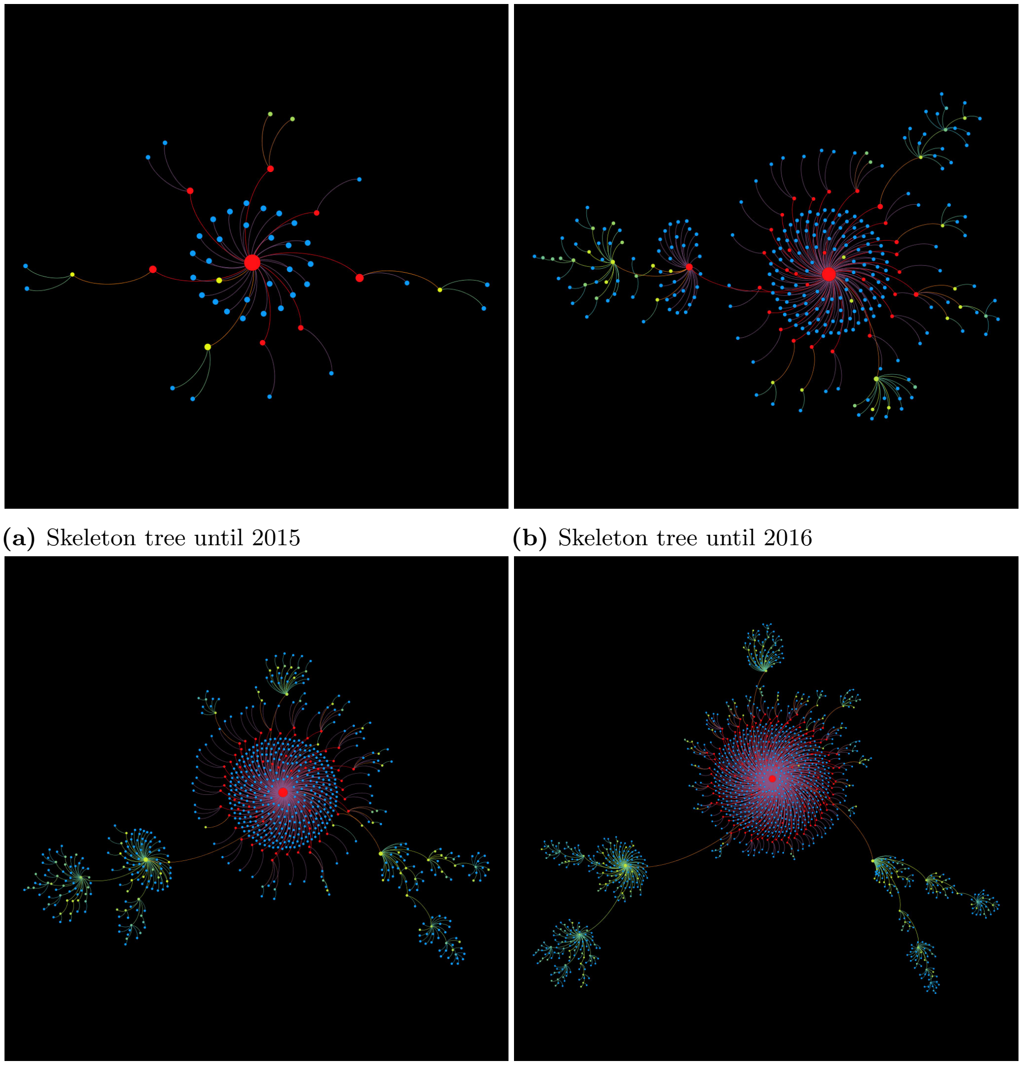

(a) Skeleton tree until 2015

(b) Skeleton tree until 2016

(c) Skeleton tree until 2017

(d) Skeleton tree until 2018

Fig S6. GRU: Skeleton tree evolution

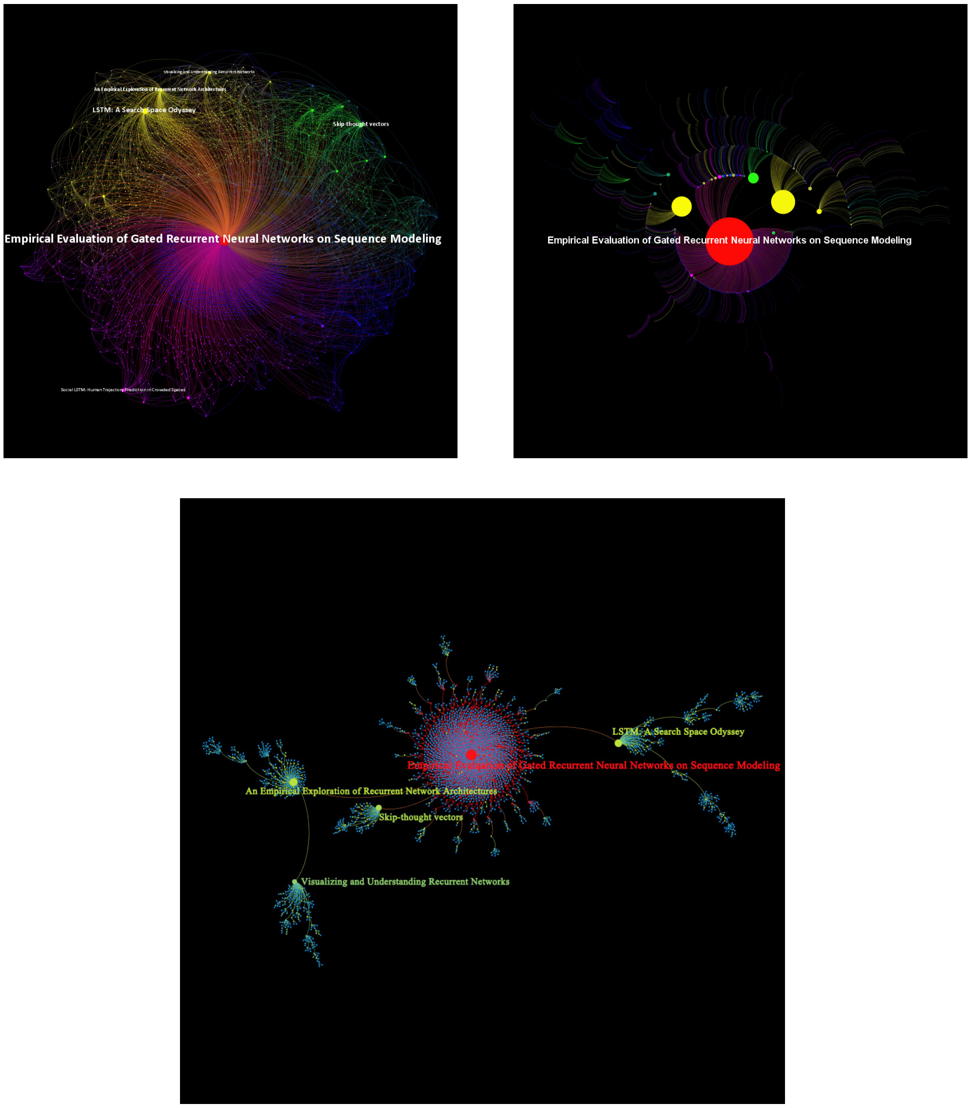

Fig S7. GRU: Galaxy map and current skeleton tree. Top left: current galaxy map. Papers with the most total citations are labelled by their titles. Top right: current skeleton tree in circular layout, node color is the same as in galaxy map. Bottom: current skeleton tree in ForceAtlas layout, node color is determined by paper knowledge temperature, with blue the coldest and red the hottest. Papers with more than 60 in-topic citations are labelled by title. Except the pioneering work, labelled nodes' size is amplified by 3 times.

title	year
Machine Health Monitoring Using Local Feature-Based Gated Recurrent Unit Networks	2018
Integrating Convolutional Neural Network and Gated Recurrent Unit for Hyperspectral Image Spectral-Spatial Classification	2018
Comparison of Deep learning models on time series forecasting : a case study of Dissolved Oxygen Prediction	2019
Anomaly Detection of Wind Turbine Generator Based on Temporal Information	2019
Energy price prediction based on independent component analysis and gated recurrent unit neural network	2019
Condition monitoring of wind turbines based on spatio-temporal fusion of SCADA data by convolutional neural networks and gated recurrent units	2019
Intelligent Fault Diagnosis of Rolling Bearing Using Adaptive Deep Gated Recurrent Unit	2019
Abnormality Diagnosis Model for Nuclear Power Plants Using Two-Stage Gated Recurrent Units	2020

Table S2. GRU: Clustering effect example. First line is the parent paper and the rest children.

2004 and 'Data Mining: Concepts and Techniques' published in 2011 have mostly extended the sub-field led by SVMPR. Judging from skeleton tree, they have not contributed as much as their parent, SVMPR (Fig. S9). As a result, the topic has been accumulating its knowledge and popularity much slower than before. Nonetheless, globally speaking, this is a rising topic.

Now we closely examine the interior of this topic. 20 years of development allows a full exploration of the mainstream ideas and a thorough heat diffusion within the topic (Fig. S9). Today, the most popular child papers all have a paper knowledge temperature above average (Fig. S10) and they serve as heat sources together with the pioneering work. As the articles are located farther away from them, paper knowledge temperature decreases globally. Paper knowledge temperature also drops evenly with article age (Fig. 6(c)). The drastic heat-level drop in biggest ages is due to the fact that the topic contains several articles published earlier than the pioneering work and these articles have few followers. Besides, the blue nodes that surround the pioneering work and the most popular child papers are papers with few or no in-topic citations (Fig. S10 bottom left). However, even if we let alone these oldest articles and the aforementioned papers with little subsequent development, the general rule "the older the hotter" is not robust. For example, article 'Data Mining: Practical Machine Learning Tools and Techniques' (DM) published in 1999 is slightly hotter than its child papers 'Discriminative vs. Generative Classifiers: An In-Depth Experimental Comparison using Cost Curves' (DGC) published in 2005 and 'Feature selection and classification in multiple class datasets' (FSC) published in 2011. DM is coloured orange while DGC and FSC are coloured orange-red. This is due to the intrinsic difference of their content, which is reflected by their distinct citations. This example also suggests that the general rule "the more influential the hotter" is weak (Fig. S63 (c)).

We observe in addition certain clustering effect in the skeleton tree. For example, all child papers of 'Selection of input parameters to model direct solar irradiance by using artificial neural networks' study the topic's application in energy radiation (Table S3). This confirms the effectiveness of our skeleton tree extraction algorithm.

When comparing T^t's evolution with the keyword frequency of the pioneering work queried from Baidu Xueshu, we find topic knowledge temperature bears similarity with the keyword occurence trend until 2015. 'neural network' is "hottest" during 2008 and

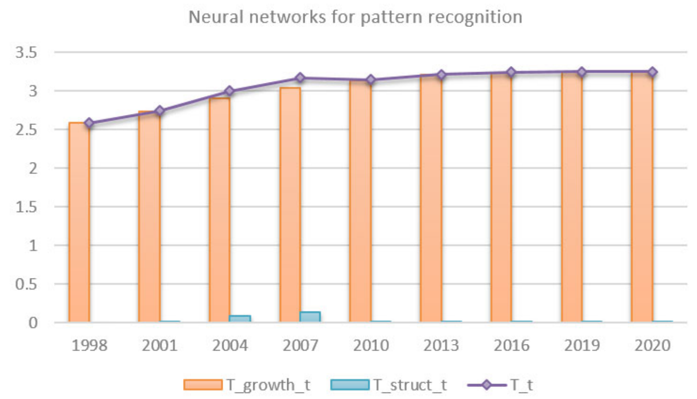

year	$\lvert V^t \rvert$	$\lvert E^t \rvert$	n_t	V_t	$UsefulInfo^t$	T^t_{growth}	T^t_{struct}	T^t
1998	586	848	494.418	586	91.582	2.583		2.583
2001	2235	3764	1779.662	2235	455.338	2.737	0.008	2.745
2004	4761	9302	3564.22	4761	1196.78	2.911	0.089	3
2007	8058	17236	5785.872	8058	2272.128	3.035	0.137	3.172
2010	11202	25723	7789.449	11202	3412.551	3.134	0.012	3.146
2013	13788	33517	9374.213	13788	4413.787	3.205	0.005	3.21
2016	15763	39120	10605.175	15763	5157.825	3.239	0.004	3.243
2019	16927	42423	11352.527	16927	5574.473	3.249	0.001	3.25
2020	17046	42748	11431.177	17046	5614.823	3.25	0	3.25

Fig S8. Pattern recognition: topic statistics and knowledge temperature evolution

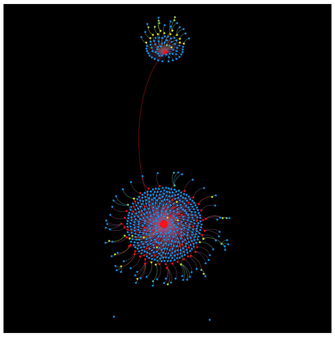

(a) Skeleton tree until 1998

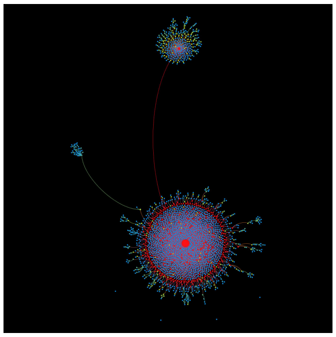

(b) Skeleton tree until 2001

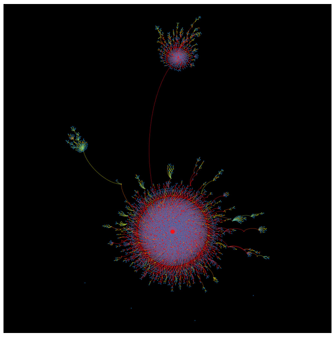

(c) Skeleton tree until 2004

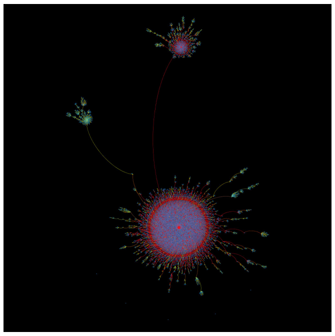

(d) Skeleton tree until 2007

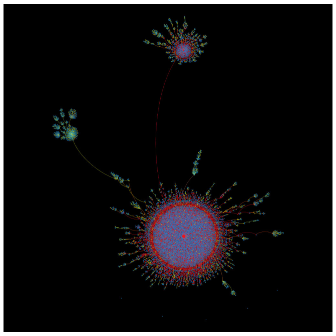

(e) Skeleton tree until 2010

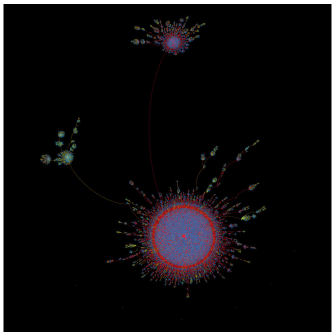

(f) Skeleton tree until 2016

Fig S9. Pattern recognition: Skeleton tree evolution

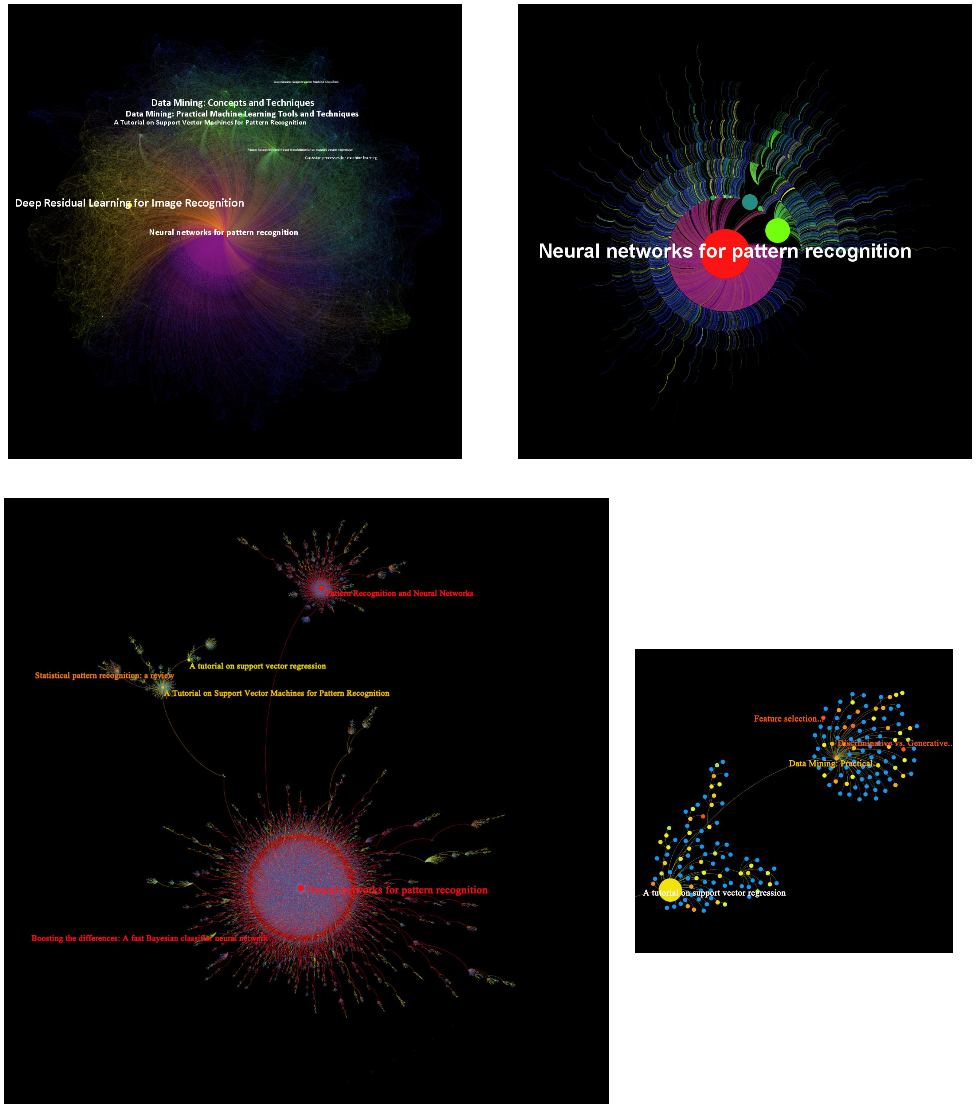

Fig S10. Pattern recognition: Galaxy map, current skeleton tree and its regional zoom. Top left: current galaxy map. Papers with the most total citations are labelled by their titles. Top right: current skeleton tree in circular layout, node color is the same as in galaxy map. Bottom left: current skeleton tree in ForceAtlas layout, node color is determined by paper knowledge temperature, with blue the coldest and red the hottest. Papers with more than 230 in-topic citations are labelled by title. Except the pioneering work, labelled nodes' size is amplified by 5 times. Bottom right: regional zoom of current skeleton tree in ForceAtlas layout.

title	year
Selection of input parameters to model direct solar irradiance by using artificial neural networks	2004
Estimation of Surface Solar Radiation with Artificial Neural Networks	2008
Improvement of temperature-based ANN models for solar radiation estimation through exogenous data assistance	2011
Splitting Global Solar Radiation into Diffuse and Direct Normal Fractions Using Artificial Neural Networks	2012
Prediction of daily global solar irradiation data using Bayesian neural network: A comparative study	2012
Assessment of ANN and SVM models for estimating normal direct irradiation (Hb)	2016

Table S3. Pattern recognition: Clustering effect example. First line is the parent paper and the rest children.

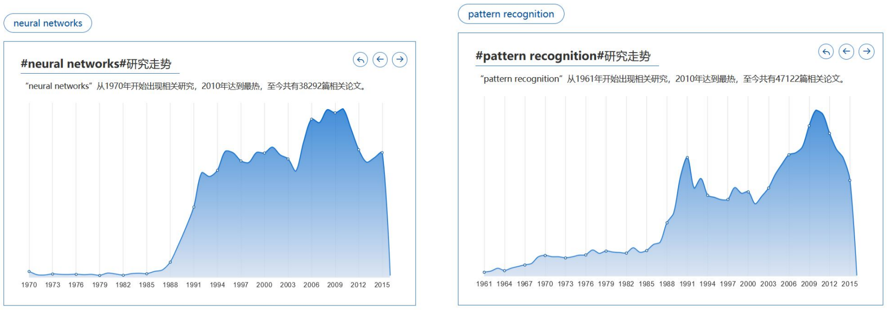

Fig S11. Pattern recognition: Popularity trend of pioneering work's keywords provided by baidu research engine.

2011 and 'pattern recognition' is "hottest" during 2009 and 2012 (Fig. S11). Indeed, we observe that useful information accumulation happens the fastest within the topic during 2007 and 2011 (Fig. 5(b)) and that T^t demonstrates a faster increase before 2010 (Fig. S8).

S2.2 Rise-then-fall Topics

S2.2.1 Critical Power for Asymptotic Connectivity in Wireless Networks

As is shown by the basic statistics and T^t_{growth}, the topic reached its peak around 2011 (Fig. S12). Indeed, this topic manifested an accelerated userful information accumulation until year 2010 (Fig 5(d)). The decline in scale growth and T^t_{growth} is obvious afterwards. The majority of popular child papers were published no later than 2004. They pushed up T^t_{growth} with their new ideas and contributed to the flourishing before 2010. In particular, popular child papers 'The capacity of wireless networks' published in 2000 and 'The number of neighbors needed for connectivity of wireless networks' published in 2004 each leads a non-trivial research sub-direction, demonstrated as clusters in the skeleton tree (Fig. S14). Their substantial extension to the topic knowledge structure is additionally illustrated by a high $T^t_{structure}$ in the early days. However, the glory did not last for long. After 2010, the continuous lack of young influential child papers gradually resulted in a decreasing topic visibility and thus a shrinking inflow of useful information, its knowledge source. The trend is also reflected in the stagnation of skeleton tree. While we are still able to detect some development on the periphery of all 3 clusters from 2007 to 2011, the skeleton tree seems to take a

definitive form after 2011. The snapshots look almost identical (Fig. S13). Consequently, both T^t_{growth} and $T^t_{structure}$ have plunged. After 10 years of golden age, the topic is now perishing.

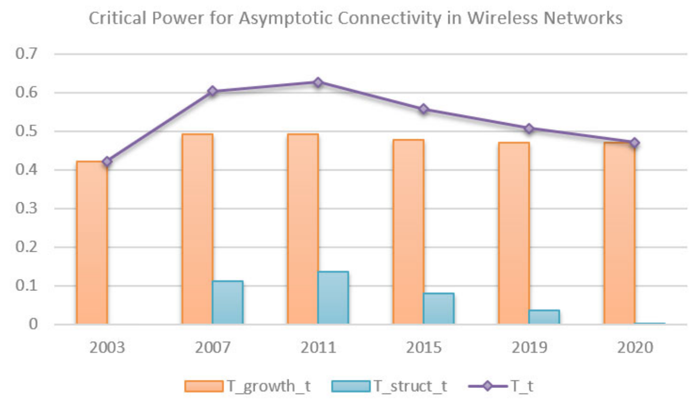

year	$\lvert V^t \rvert$	$\lvert E^t \rvert$	n_t	V_t	$UsefulInfo^t$	T^t_{growth}	T^t_{struct}	T^t
2003	83	236	41.688	83	41.312	0.422		0.422
2007	412	1697	177.643	412	234.357	0.492	0.111	0.603
2011	783	3514	337.789	783	445.211	0.492	0.135	0.626
2015	992	4607	440.339	992	551.661	0.478	0.079	0.557
2019	1074	4984	484.238	1074	589.762	0.47	0.037	0.507
2020	1078	4998	486.525	1078	591.475	0.47	0	0.47

Fig S12. Critical Power: topic statistics and knowledge temperature evolution

Now we closely examine the heat distribution within the topic (Fig. S14). We observe a quick heat diffusion during the flourishing period (Fig. S13(b,c)). Now heat diffusion is complete as popular child papers all have a knowledge temperature above average and the child papers published during the golden period are relatively hot in general (Fig. 6(d)). An obvious exception lies in the oldest child papers. Their low average temperature is because they were published at the same time or earlier than the pioneering work and they have few or no followers. Besides the pioneering work, popular child paper 'The capacity of wireless networks' is also a heat source within today's topic (Fig. S14) bottom left). As articles are located farther away from them, they gradually cool down. The blue nodes that surround the pioneering work and the popular child paper 'The capacity of wireless networks' in central clusters are papers with few or no in-topic followers. However, the general rules "the older the hotter" and "the more influential the hotter" (Fig. S63(d)) are not robust. For instance, paper 'New perspective on sampling-based motion planning via random geometric graphs' (SBMP) published in 2018 is hotter than its parent, 'CONNECTIVITY OF SOFT RANDOM GEOMETRIC GRAPHS' (CSRG), an article published in 2016. SBMP has an average knowledge temperature while CSRG has a temperature below average. This can be mainly attributed to their different research focus, which is reflected by their distinct citations and citations' average heat-level. Another reason may be that even though CSRG has had a much better development, the dozen articles it has inspired have gained little popularity and impact, thus they do not help boost CSRG's status.

We find article 'Power Control in Ad-Hoc Networks: Theory, Architecture, Algorithm and Implementation of the COMPOW Protocol' particularly interesting. In current skeleton tree, it is situated right between 2 clusters respectively led by 'The capacity of wireless networks' and 'The number of neighbors needed for connectivity of wireless networks'. It has not directly inspired many subsequent researches on its own,

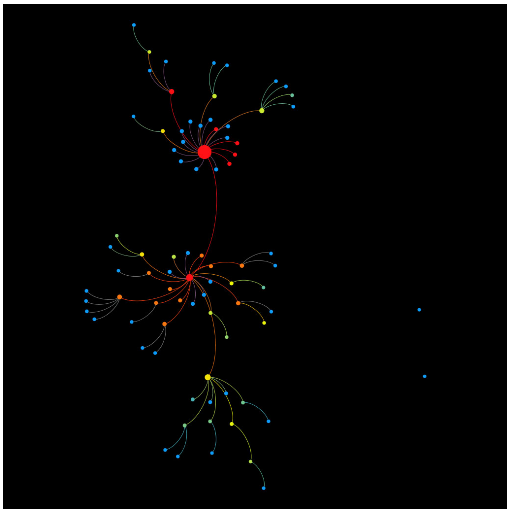

(a) Skeleton tree until 2003

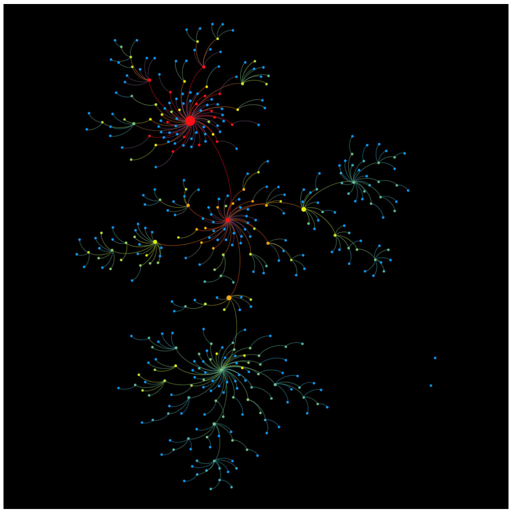

(b) Skeleton tree until 2007

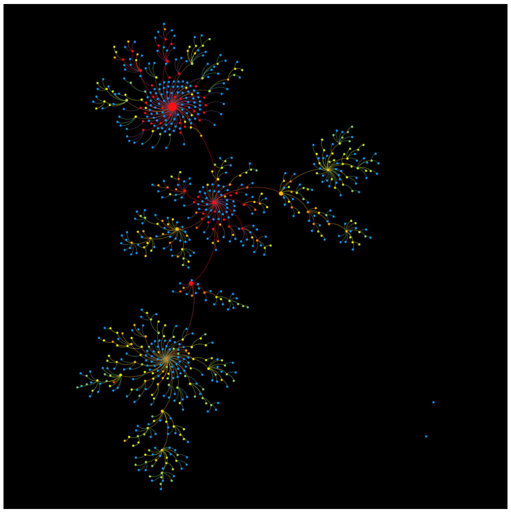

(c) Skeleton tree until 2011

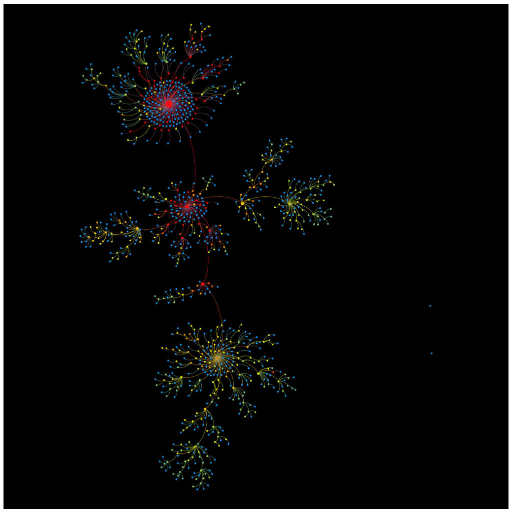

(d) Skeleton tree until 2015

Fig S13. Critical Power: Skeleton tree evolution

title	year
CONNECTIVITY OF SOFT RANDOM GEOMETRIC GRAPHS	2016
Isolation and Connectivity in Random Geometric Graphs with Self-similar Intensity Measures	2018
On Resilience and Connectivity of Secure Wireless Sensor Networks Under Node Capture Attacks	2017
New perspective on sampling-based motion planning via random geometric graphs	2018

Table S4. Critical Power: Clustering effect example. First line is the parent paper and the rest children.

but it is among the hottest papers in this topic. The phenomenon suggests that although the article itself may not have a big impact, it has high value in enlightment because it has indirectly inspired a handful of influential literature through one of its child papers. The ability for a publication to pass on knowledge and inspire others is also a promising indicator for high popularity.

We observe in addition certain clustering effect in the skeleton tree (Table S4). For example, almost all child papers of 'CONNECTIVITY OF SOFT RANDOM GEOMETRIC GRAPHS' have similar research themes as itself. This confirms the effectiveness of our skeleton tree extraction algorithm.

When comparing T^t's evolution with the keyword frequency of the pioneering work queried from Baidu Xueshu, we find topic knowledge temperature bears similarity with the keyword occurence trend until 2015. Starting from 2003, 'critical power' stays relatively "hot" during 2004 and 2010 (Fig. S15). Indeed, we observe that useful information accumulation happens the fastest within the topic during 2007 and 2011 (Fig. 5(d)) and that during the period, T^t also climbs to its peak (Fig. S12).

S2.2.2 The capacity of wireless networks

As is shown by T^t, the topic reached its peak at some time around 2007 (Fig. S16). The batch of popular child papers arriving between 2001 and 2004, namely 'Capacity of Ad hoc wireless networks', 'Mobility increases the capacity of ad-hoc wireless networks', 'A network information theory for wireless communication: scaling laws and optimal operation' and 'Impact of interference on multi-hop wireless network performance', largely enriched the topic knowledge base by inspiring several research sub-fields, as is reflected by the significant structure advancement in skeleton tree from 2003 to 2007 (Fig. S17). As a result, we observe a soar both in T^t_{growth} and $T^t_{structure}$. Popular child papers continued to come until 2007. But the younger ones did not cause a stir as much. Only 1 of them has made visible contribution to knowledge structure evolution: 'Closing the Gap in the Capacity of Wireless Networks Via Percolation Theory' published in 2007 opened up a new research focus and led to the end division of a major branch in the skeleton tree by 2011. The decreasing exposure gained by its child papers and a decelerating evolution in knowledge pattern caused $T^t_{structure}$ to drop after 2007. But the residual attractiveness continued to draw a abundant quantity of "new blood" and ensured the rise in T^t_{growth} for a while longer. This fact is also captured by a continual rise in average annual useful information accumulation (Fig. 5(e)). After 2011, despite a continuous growth in total publication number, the topic fails to collect as much useful information as before due to an overall mediocre development of child papers published after 2009. As a result, it has been gradually phased out. The wear-off of the community's focus is illustrated by an immediate drop in $T^t_{structure}$ in 2015, which also accounts for the down trend of T^t. Correspondingly, we observe fewer remarkable changes in skeleton tree during this period. While the cooling-down is mainly due to

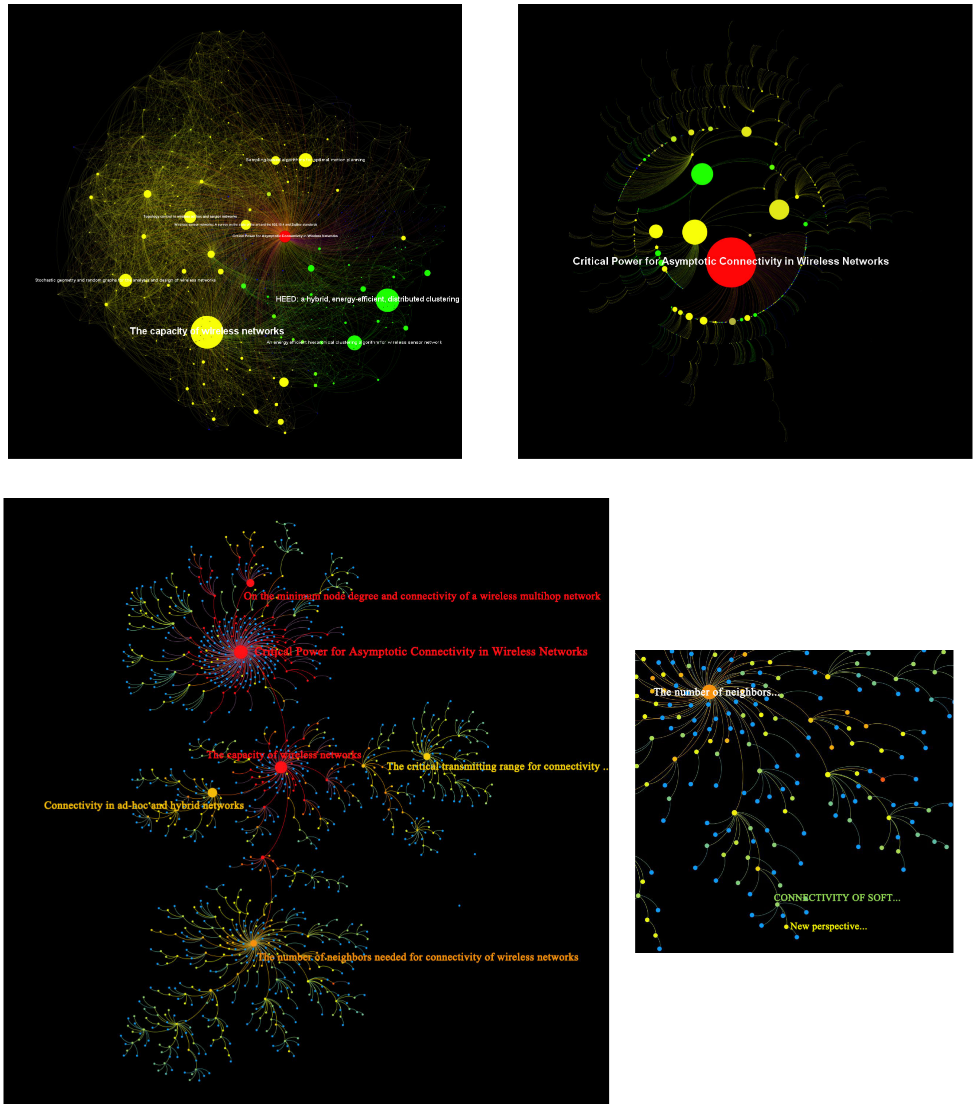

Fig S14. Critical Power: Galaxy map, current skeleton tree and its regional zoom. Top left: current galaxy map. Papers with the most total citations are labelled by their titles. Top right: current skeleton tree in circular layout, node color is the same as in galaxy map. Bottom left: current skeleton tree in ForceAtlas layout, node color is determined by paper knowledge temperature, with blue the coldest and red the hottest. Papers with more than 100 in-topic citations are labelled by title. Except the pioneering work, labelled nodes' size is amplified by 3 times. Bottom right: regional zoom of current skeleton tree in ForceAtlas layout.

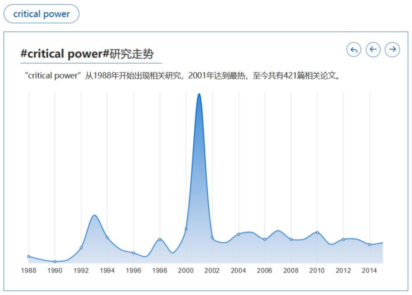

Fig S15. Critical Power: Popularity trend of pioneering work's keywords provided by baidu research engine.

attention loss before 2015, recent temperature drop is caused by knowledge supply shortage. The focus loss has eventually resulted in diminishing publications and affected its long-term knowledge accumulation. To sum up, after around 10 years of glory, the topic is now going downhill.

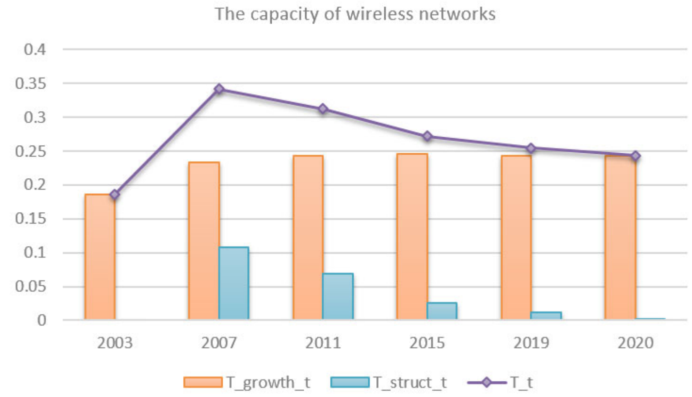

year	$\|V^t\|$	$\|E^t\|$	n_t	V_t	$UsefulInfo^t$	T^t_{growth}	T^t_{struct}	T^t
2003	325	860	197.224	325	127.776	0.186		0.186
2007	2220	10999	1076.03	2220	1143.97	0.233	0.108	0.342
2011	4956	30466	2302.961	4956	2653.039	0.243	0.07	0.313
2015	6867	46263	3152.523	6867	3714.477	0.246	0.026	0.272
2019	7621	51667	3535.877	7621	4085.123	0.244	0.011	0.255
2020	7644	51789	3546.091	7644	4097.909	0.244	0	0.244

Fig S16. Capacity Wireless Network: topic statistics and knowledge temperature evolution

Now we probe into the topic and closely examine the heat distribution in its latest skeleton tree (Fig. S18 bottom). After 20 years of development, the heat diffusion is nearly completed as popular child papers all have a knowledge temperature above average and the child papers published in the first 10 years are relatively hot in general (Fig. 6(e)). The popular child papers and the pioneering work are the multiple heat sources within the topic. If we let alone the blue nodes surrounding the pioneering work and popular child papers, which are papers with few or without any in-topic citations, it is clear that paper knowledge temperature decreases globally as the articles are located farther away from them. However, there are exceptions to general rules "the more influential the hotter" (Fig. S63(e)) and "the older the hotter". For example, paper 'Mobility increases the capacity of ad-hoc wireless networks' (MAWN) published in 2001,

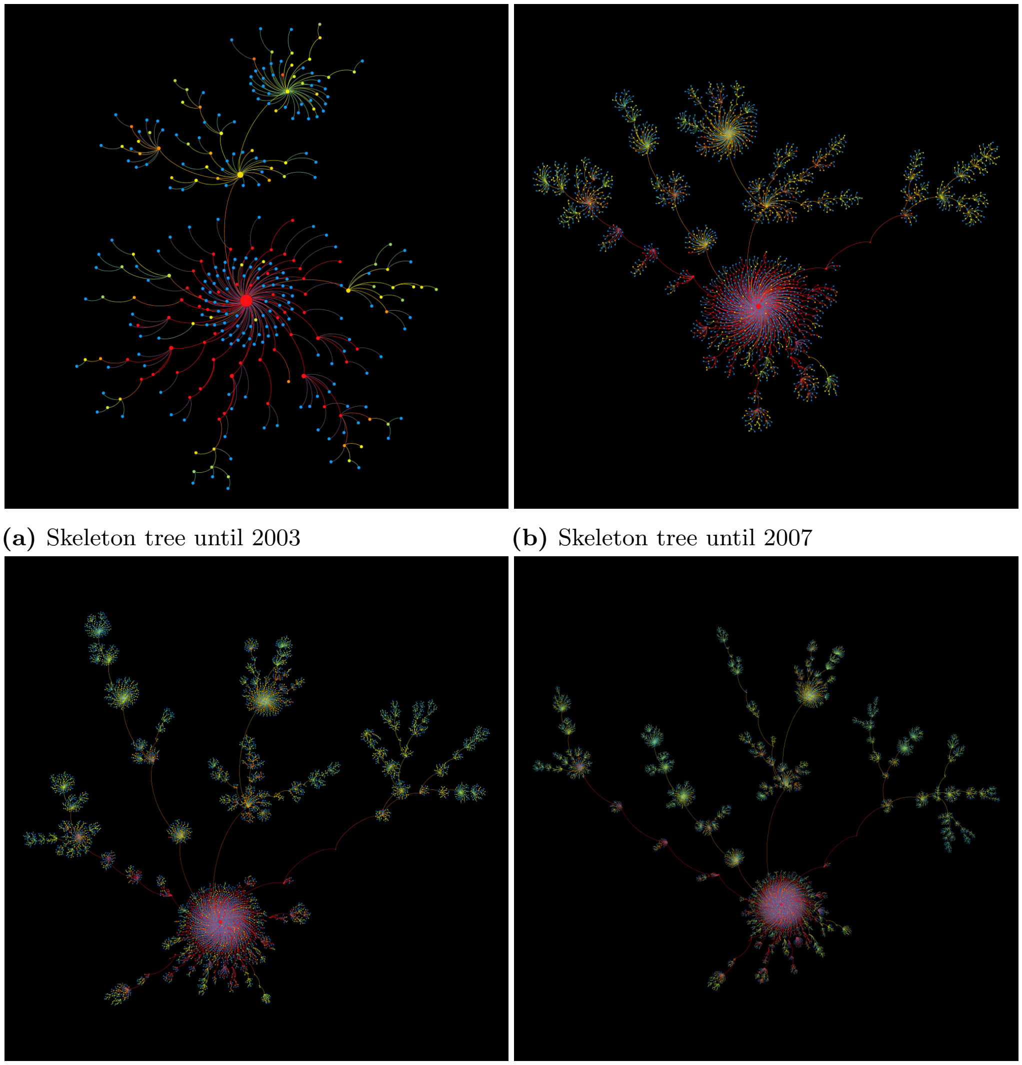

(a) Skeleton tree until 2003
(b) Skeleton tree until 2007
(c) Skeleton tree until 2011
(d) Skeleton tree until 2015

Fig S17. Capacity Wireless Network: Skeleton tree evolution

title	year
A Delay-Efficient Algorithm for Data Aggregation in Multihop Wireless Sensor Networks	2011
In-Network Estimation with Delay Constraints in Wireless Sensor Networks	2013
Estimate Aggregation with Delay Constraints in Multihop Wireless Sensor Networks	2011
Genetic Local Search for Conflict-Free Minimum-Latency Aggregation Scheduling in Wireless Sensor Networks	2018
Interference-Fault Free Data Aggregation in Tree-Based WSNs	2016
GLS and VNS Based Heuristics for Conflict-Free Minimum-Latency Aggregation Scheduling in WSN.	2019
Data Aggregation Scheduling Algorithms in Wireless Sensor Networks: Solutions and Challenges	2014
Efficient scheduling for periodic aggregation queries in multihop sensor networks	2012
Layer-Based Data Aggregation and Performance Analysis in Wireless Sensor Networks	2013
Neither Shortest Path Nor Dominating Set: Aggregation Scheduling by Greedy Growing Tree in Multihop Wireless Sensor Networks	2011
Composite interference mapping model for Interference Fault-Free Transmission in WSN	2015
Weighted fairness guaranteed data aggregation scheduling algorithm in wireless sensor networks	2012
A fuzzy-rule-based packet reproduction routing for sensor networks	2018

Table S5. Capacity Wireless Network: Clustering effect example. First line is the parent paper and the rest children.

which is at the junction between the central cluster and a principal branch, is slightly colder than 2 of its children: ‘Design challenges for energy-constrained ad hoc wireless networks’ (DCAWN) published in 2002 and ‘Unreliable sensor grids: coverage, connectivity and diameter’ (USG) published in 2003. MAWN is coloured orange while DCAWN and USG are coloured orange-red and red. The main reason of this uncommon phenomenon is their different research focus, which is reflected by their distinct citations and citations’ average heat-level. Another reason may be that even though MAWN has inspired much more child papers, few of its numerous followers have so far achieved remarkable development, hence their limited boosting effect.

We observe in addition certain clustering effect in the skeleton tree (Table S5). For example, almost all child papers of ‘A Delay-Efficient Algorithm for Data Aggregation in Multihop Wireless Sensor Networks’ have similar research themes as itself. This proves the effectiveness of our skeleton tree extraction algorithm.

When comparing T^t’s evolution with the keyword frequency of the pioneering work queried from Baidu Xueshu, we find topic knowledge temperature well corresponds with the keyword occurence trend until 2015. Starting from 2003, ‘channel capacity’ is the “hottest” between 2008 and 2009 (Fig. S19). Indeed, we observe that T^t reaches its highest value around 2007 (Fig. S16).

S2.2.3 Efficient Estimation of Word Representations in Vector Space

The popularity and impact gain until 2019 is mainly due to a fast accumulation of useful information (Fig. 5(f)). By the end of 2013, 2 influential child papers, ‘Linguistic Regularities in Continuous Space Word Representations’ (LRCSWR) and ‘Distributed Representations of Words and Phrases and their Compositionality’ (DRWPC) had

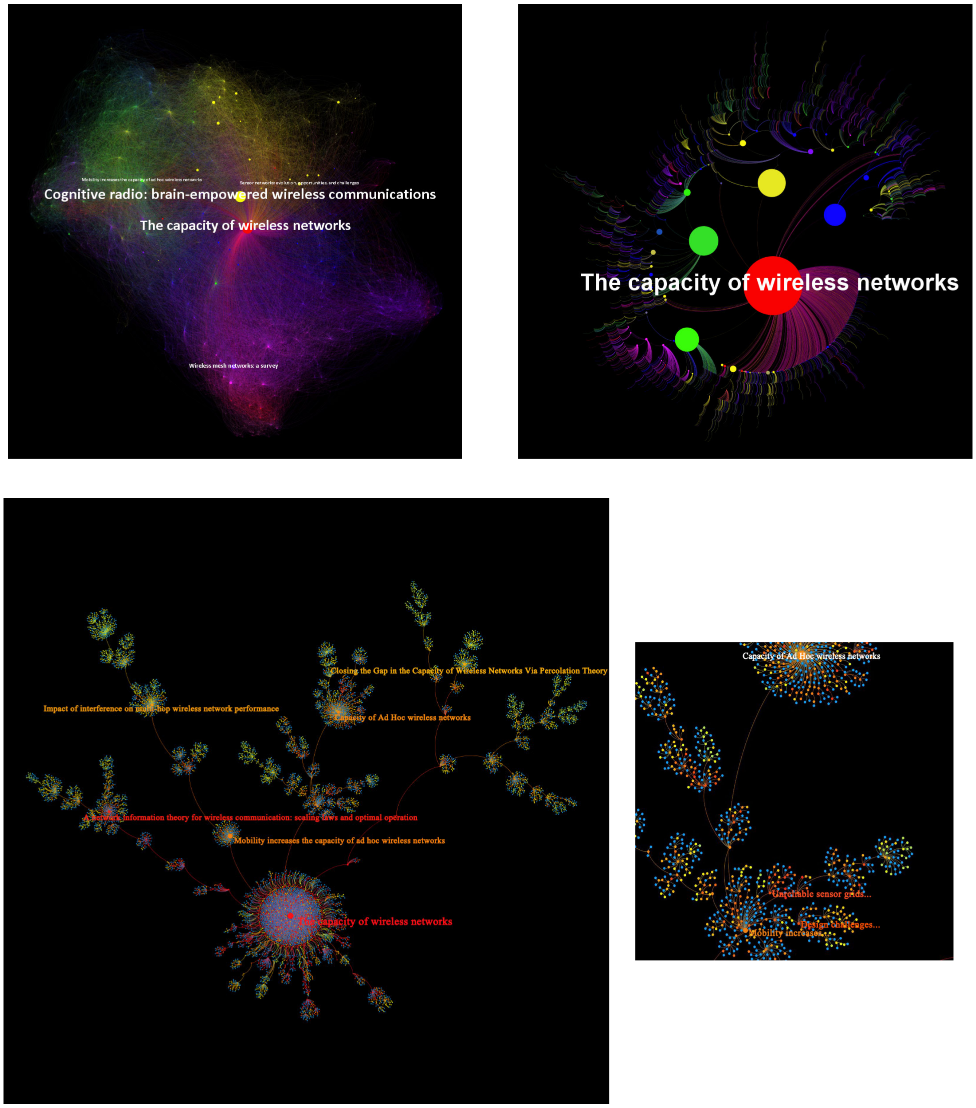

Fig S18. Capacity Wireless Network: Galaxy map, current skeleton tree and its regional zoom. Top left: current galaxy map. Papers with the most total citations are labelled by their titles. Top right: current skeleton tree in circular layout, node color is the same as in galaxy map. Bottom left: current skeleton tree in ForceAtlas layout, node color is determined by paper knowledge temperature, with blue the coldest and red the hottest. Papers with more than 500 in-topic citations are labelled by title. Except the pioneering work, labelled nodes' size is amplified by 3 times. Bottom right: regional zoom of current skeleton tree in ForceAtlas layout.

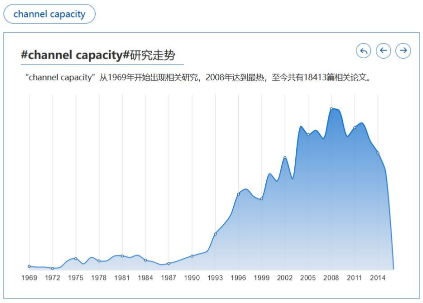

Fig S19. Capacity Wireless Network: Popularity trend of pioneering work's keywords provided by baidu research engine.

formed the fundamentals of topic knowledge structure. LRCSWR is depicted as the red node in the middle of the then skeleton tree and its child, DRWPC, is represented by the yellow-green node above itself (Fig. S21(a)). During the next 2 years, the topic expanded quickly thanks to the substantial development of all 3 papers. DRWPC emerged as the second topic center following the pioneering work (Fig. S21(b)). In addition, DRWPC helped extending topic knowledge structure by inspiring a new research direction. This research branch later proved to be a novel research focus. Starting from 2016, owing to a multidimensional development the topic has been maintaining a knowledge reserve quantity corresponding to its size, which is reflected by its steady T^t_{growth} (Fig. S20). More importantly, the research branch that emerged by the end of 2015 has developed into 2 new non-trivial research directions due to the popularity rise in 2 child papers published in 2014: 'Glove: Global Vectors for Word Representation' (Glove) and 'Distributed Representations of Sentences and Documents' (DRSD). They brought new knowledge, attracted the attention of the latest research attention, and catalysed an accelerated topic knowledge structure evolution, which is captured by a rising $T^t_{structure}$. This year, there has not been any significant new trend so far. Therefore, the topic cools down a bit due to a $T^t_{structure}$ drop. Unless the topic succeeds in "breeding" some new focus or having some breakthrough to existing sub-topics in the near future, it starts to go downhill after 6 years of thriving.

Now we probe into the topic and closely examine the heat distribution in its latest skeleton tree (Fig. S22 bottom). The topic's fast development accompanies a continuous heat diffusion. The older popular child papers has become the hottest since 2015 and the younger ones, namely DRSD and Glove, has recently evolved into topic's new heat sources. It is clear that paper knowledge temperature decreases globally as the articles are located farther away from them. This phenomenon fits the general rule "the older the hotter" (Fig. 6(f)) and "the more influential the hotter" (Fig. S63(f)). Note that the blue nodes that surround the pioneering work and popular child papers in central parts are papers with few or without any in-topic citations.

We observe in addition certain clustering effect in the skeleton tree (Table S6). For example, in current skeleton tree, all child papers of 'Sentiment Embeddings with Applications to Sentiment Analysis' published in journal *IEEE Transactions on Knowledge and Data Engineering* in 2016 specialize in sentiment analysis. This proves the effectiveness of our skeleton tree extraction algorithm.

S2.2.4 Coverage problems in wireless ad-hoc sensor networks

This topic reached its peak around 2010 thanks to a surge in $T^t_{structure}$ and a continous mild increase in T^t_{growth} (Fig. S23). Most of its popular child papers were published by the end of 2006. Their collective appeal to new researches contributed to an accelerated

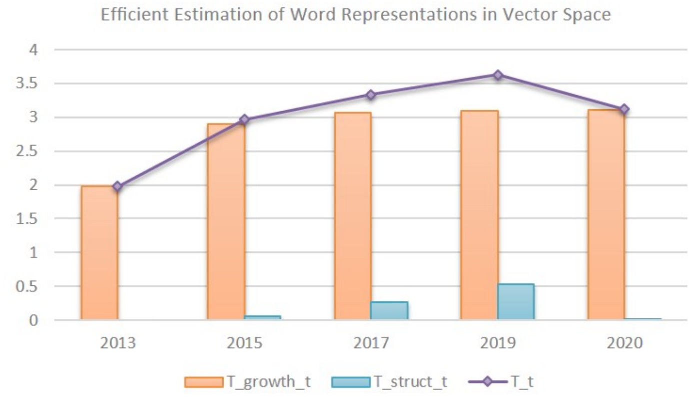

year	$\lvert V^t \rvert$	$\lvert E^t \rvert$	n_t	V_t	$UsefulInfo^t$	T^t_{growth}	T^t_{struct}	T^t
2013	29	42	23.5	29	5.5	1.978		1.978
2015	1197	4014	660.232	1197	536.768	2.91	0.061	2.967
2017	4136	16798	2159.275	4136	1976.725	3.07	0.268	3.338
2019	7736	34285	3999.585	7736	3736.415	3.1	0.53	3.63
2020	8133	36219	4199.586	8133	3933.414	3.104	0.015	3.119

Fig S20. Efficient word representation: topic statistics and knowledge temperature evolution

title	year
Sentiment Embeddings with Applications to Sentiment Analysis	2016
Deep Learning Adaptation with Word Embeddings for Sentiment Analysis on Online Course Reviews	2020
Learning Word Representations for Sentiment Analysis	2017
Improving Aspect-Based Sentiment Analysis via Aligning Aspect Embedding	2019
Attention-based long short-term memory network using sentiment lexicon embedding for aspect-level sentiment analysis in Korean	2019
Deep Learning for Aspect-Based Sentiment Analysis: A Comparative Review	2019
An efficient preprocessing method for supervised sentiment analysis by converting sentences to numerical vectors: a twitter case study	2019
Deep learning for sentiment analysis: A survey	2018
Deep Learning in Sentiment Analysis	2018
Sentiment analysis using deep learning approaches: an overview	2020

Table S6. Efficient word representation: Clustering effect example. First line is the parent paper and the rest children.

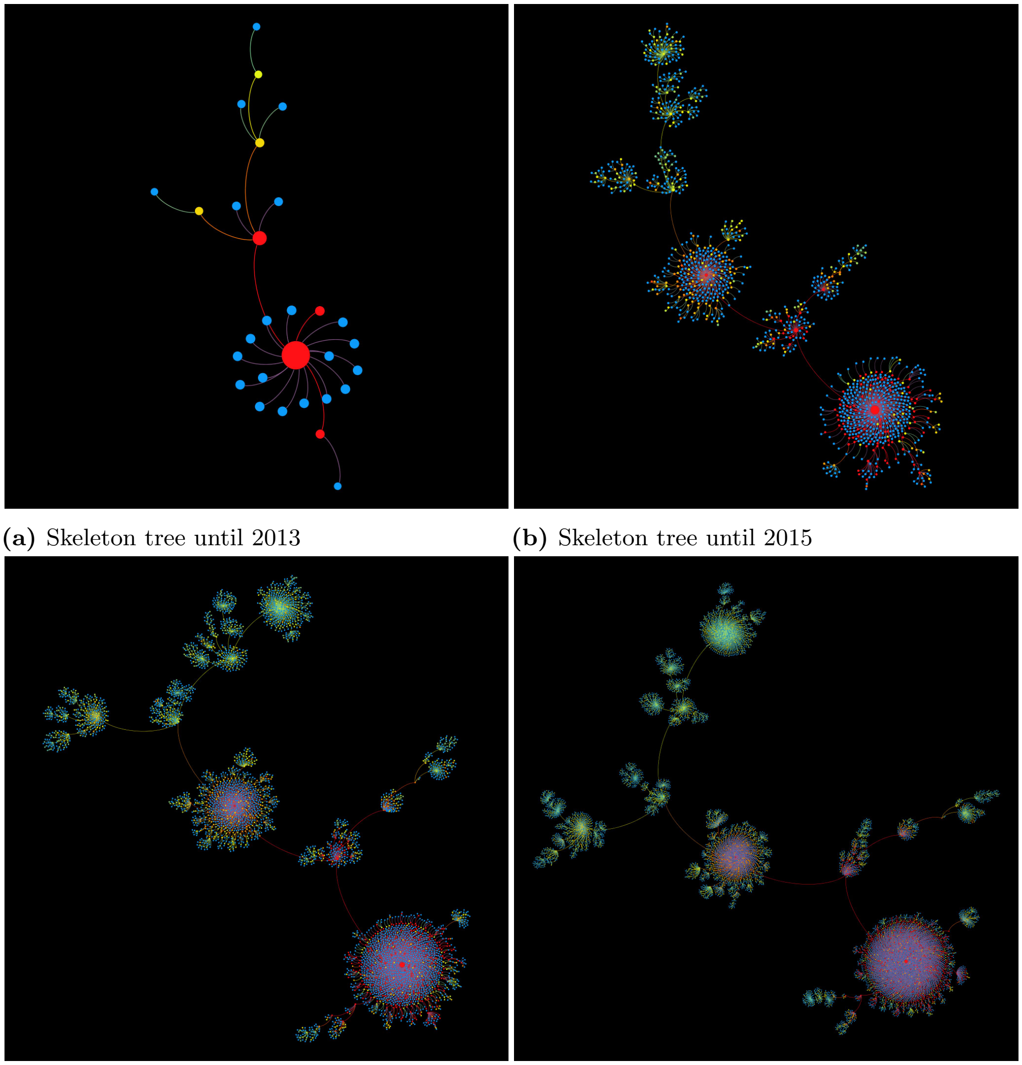

(a) Skeleton tree until 2013
(b) Skeleton tree until 2015
(c) Skeleton tree until 2017
(d) Skeleton tree until 2019

Fig S21. Efficient word representation: Skeleton tree evolution

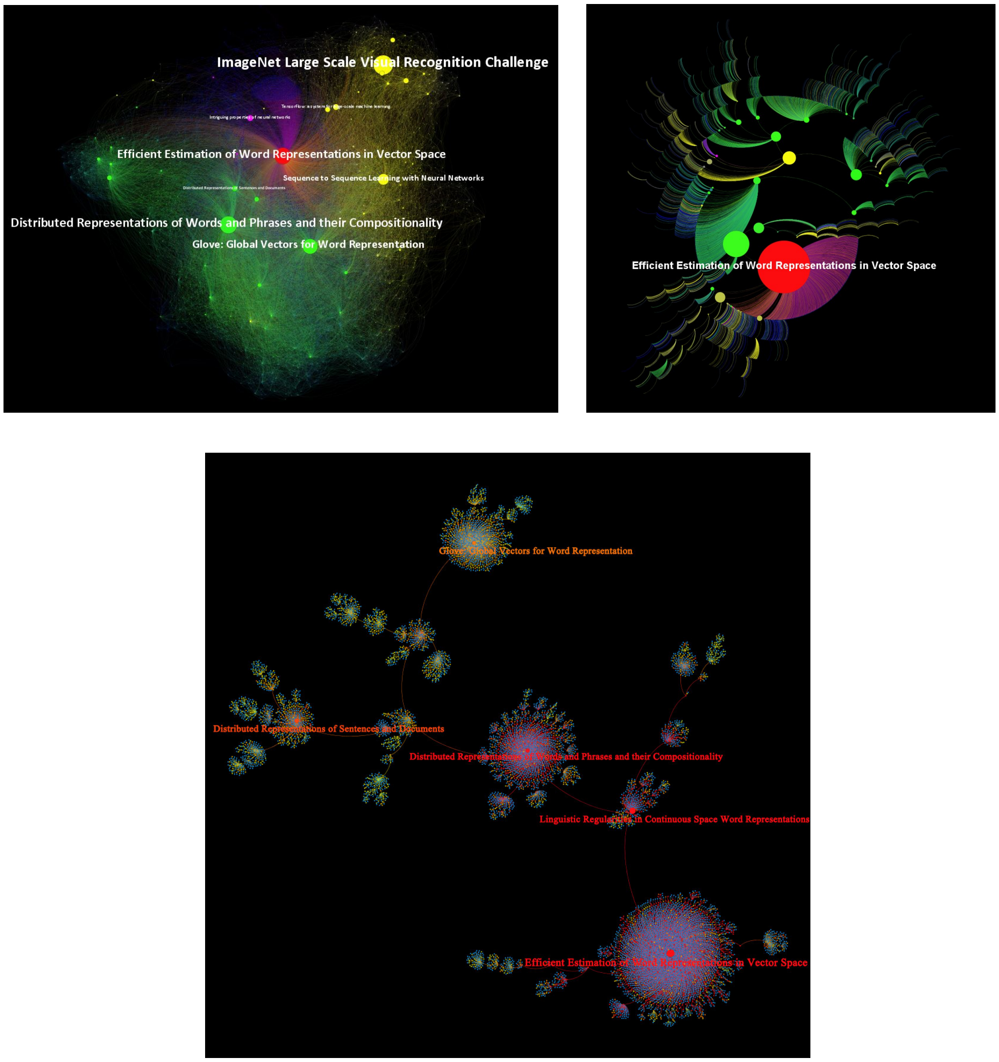

Fig S22. Efficient word representation: Galaxy map and current skeleton tree. Top left: current galaxy map. Papers with the most total citations are labelled by their titles. Top right: current skeleton tree in circular layout, node color is the same as in galaxy map. Bottom: current skeleton tree in ForceAtlas layout, node color is determined by paper knowledge temperature, with blue the coldest and red the hottest. Papers with more than 700 in-topic citations are labelled by title. Except the pioneering work, labelled nodes' size is amplified by 3 times.

increase in topic useful information until 2010 (Fig. 5(g)). Among them, the older ones laid the foundation of multiple research sub-directions and the younger ones further developed these new research branches. For instance, papers 'Unreliable sensor grids: coverage, connectivity and diameter' and 'Sensor placement for grid coverage under imprecise detections' published in 2002 and 2003 extended primarily the idea of the pioneering work. They formed the 2 big branches surrounding the central cluster in skeleton tree by 2007 (Fig. S24(a), S25). Paper 'The coverage problem in a wireless sensor network' (CPWS) published in 2005, however, created a second smaller cluster by furthering the study of his predecessor 'Localized algorithms in wireless ad-hoc networks: location discovery and sensor exposure' (LAWAN) published in 2001. Other popular papers published between 2005 and 2006 were split into 2 parties, one group supporting the growth in central cluster led by the pioneering work, the other group enriching the newer cluster built essentially by CPWS. As a result, we observe non-trivial growth in every corner of the skeleton tree during 2007 and 2010 (Fig. S24(b)). Nonetheless, along with the multidimensional flourishing, the knowledge structure started its gravity redistribution due to the maturation of the research sub-directions. This silent transformation is captured by the high $T^t_{structure}$ around 2010. The aforementioned popular child papers as well as their inspirations for future works also make great contributions to the knowledge accumulation. They helped push up T^t_{growth} until 2010. Afterwards, the topic experienced first an absence of promising child papers and then a decline in useful information supply due to its decelerated expansion. Consequently, T^t_{growth} has stagnated. The skeleton tree has unsurprisingly lost its vigor during this period (Fig. S24 (c,d)). To sum up, this topic, after a rapid development in its early days, demonstrates now a decreasing activity and a diminishing popularity and impact.

The topic's skeleton tree is a bit special in that it is comprised of 2 parts. The separation is due to the isolation of LAWAN from the pioneering work. LAWAN cites both the pioneering work and 'Dynamic fine-grained localization in Ad-Hoc networks of sensors' (DLANS). Because of a closer relation between LAWAN and DLANS, its connection to the pioneering work is cut off in skeleton tree extraction. A similar reason caused the separation of DLANS and the pioneering work. LAWAN, along with several intimately related papers, is thus completely separated from the pioneering work. They form a mini bundle beside the central cluster in 2004 skeleton tree. Shortly after, the arrival of popular child paper, CPWS, largely developed this tiny bundle and turned it into the big aggregation under the central cluster (Fig. S24).

Now we closely examine the heat distribution within the topic (Fig. S25 bottom). After 19 years of development, the heat diffusion is nearly completed as most popular child papers have a knowledge temperature above average and the child papers published during the flourishing period are relatively hot in general (Fig. 6(g)). Half of the most popular child papers serve as heat sources and paper knowledge temperature decreases globally as the articles are located farther away from them. This corresponds with the general rule "the older the hotter". Yet as several papers published at the same time as the pioneering work either have had few development or have not been cited by any recent works, they are the coldest and thus bring down the average knowledge of the oldest articles. In addition, the blue nodes that surround the pioneering work and popular child papers are papers with few or without any in-topic followers. However, we still find exceptions even if we let alone the oldest papers. Paper 'Minimal and maximal exposure path algorithms for wireless embedded sensor networks' (MMEPA) published in 2003 is colder than, for instance, its child 'Smart Path-Finding with Local Information in a Sensory Field' published in 2006 and 'An Algorithm for Target Traversing Based on Local Voronoi Diagram' published in 2007. These 2 child papers, represented as orange nodes, have a knowledge temperature above average yet the MMEPA has a knowledge temperature below average, as it is depicted by a green

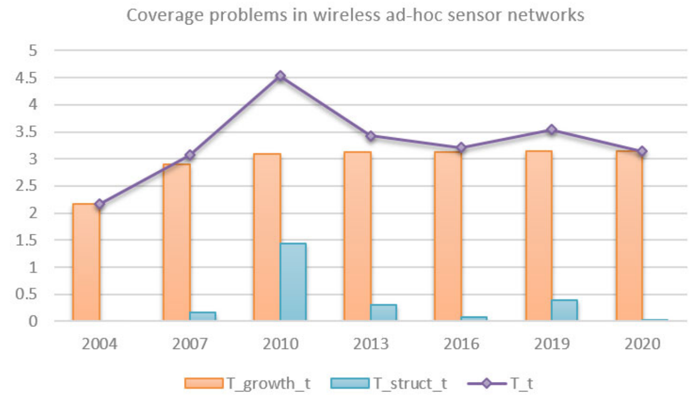

year	$\lvert V^t \rvert$	$\lvert E^t \rvert$	n_t	V_t	$UsefulInfo^t$	T^t_{growth}	T^t_{struct}	T^t
2004	146	337	90.189	146	55.811	2.166		2.166
2007	542	2420	249.982	542	292.018	2.902	0.17	3.072
2010	972	5118	420.165	972	551.835	3.096	1.44	4.536
2013	1313	7325	562.623	1313	750.377	3.123	0.308	3.431
2016	1490	8460	637.376	1490	852.624	3.129	0.08	3.209
2019	1544	8846	657.378	1544	886.622	3.143	0.396	3.539
2020	1546	8865	658.507	1546	887.493	3.142	0.001	3.143

Fig S23. Coverage problems: topic statistics and knowledge temperature evolution

node. This is mainly due to their relatively different research focus as most of their in-topic citations do not overlap with one another. Another reason may be that even though MMEPA has inspired much more child papers, few of them have achieved remarkable development, hence their limited boosting effect. In addition, this counter example also suggests that the general rule “the more influential the hotter” is very weak in this topic (Fig. S63(g)).

When comparing T^t's evolution with the keyword frequency of the pioneering work queried from Baidu Xueshu, we find topic knowledge temperature well corresponds with the keyword occurence trend until 2015. Starting from 2004, keywords ‘abcd rule’ and ‘actinic keratosis’ become the “hottest” between 2009 and 2012 (Fig. S26). Another keyword, ‘sensor networks’ is the “hottest” during 2007 and 2010. Indeed, we observe that T^t reaches its highest value around 2010 (Fig. S23).

S2.2.5 A neural probabilistic language model

Unlike many topics that welcome the majority of their popular child papers shortly after their birth, this topic waited for a long time. Most of its prominent child papers came during 2010 and 2014. Their arrival opened up new research sub-fields (Fig. S29) and infused much vigor and new knowledge to the topic, which strongly boosted T^t_{growth} during 2011 and 2015 (Fig. S27). Although the topic continued to grow fast after 2015, few child papers stood out and none has created new research focus so far. As a result, we observe a slight slow down in useful information accumulation during period 2015-2017 than period 2013-2015 (Fig. 5(h)). The knowledge accumulation process is affected by the overall quality slump and the topic started to cool down owing to the lack of new outstanding ideas. In terms of knowledge structure evolution, the topic manifests a smooth and steady progress (Fig. S28). Since the arrival of popular child papers is quite evenly spanned over 2010 and 2014, their contribution to the thriving is more reflected as knowledge and impact accumulation than a short-term popularity

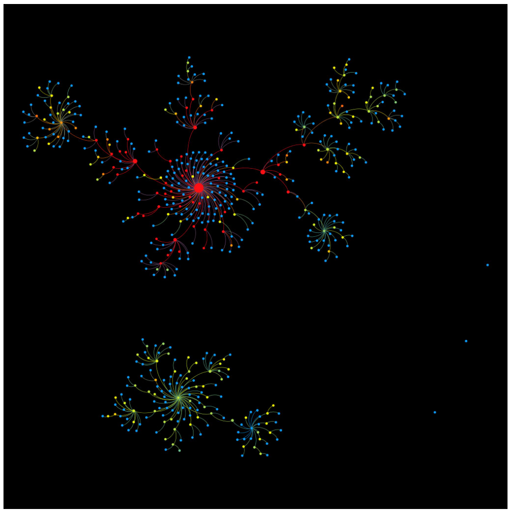

(a) Skeleton tree until 2007

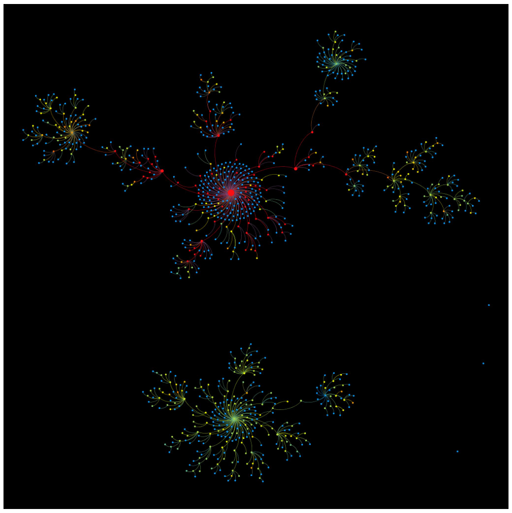

(b) Skeleton tree until 2010

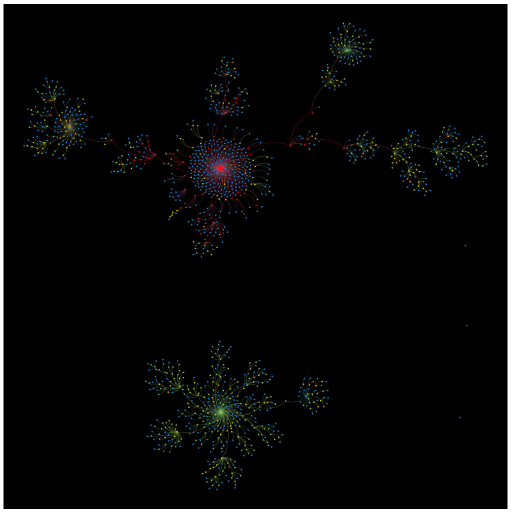

(c) Skeleton tree until 2013

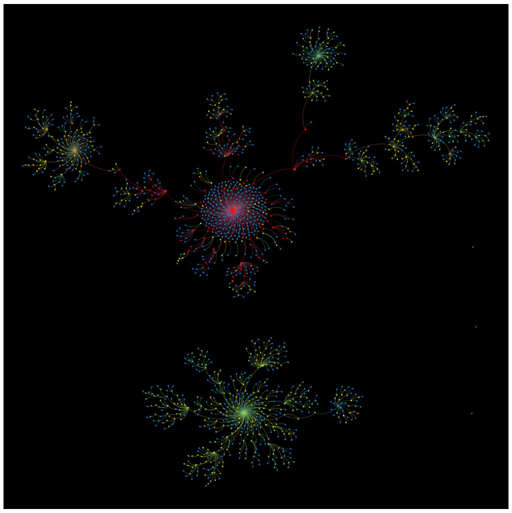

(d) Skeleton tree until 2016

Fig S24. Coverage problems: Skeleton tree evolution

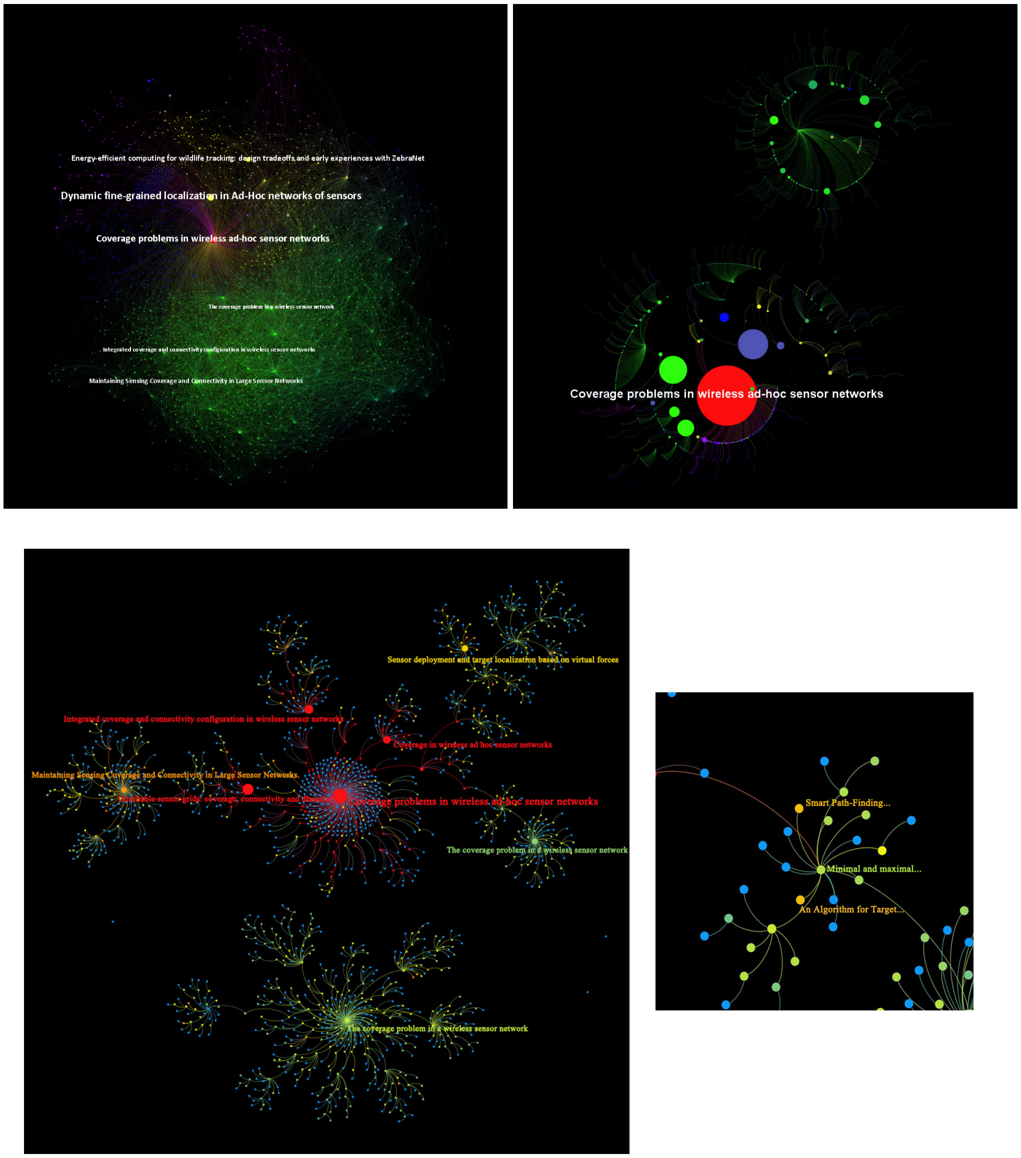

Fig S25. Coverage problems: Galaxy map, current skeleton tree and its regional zoom. Top left: current galaxy map. Papers with the most total citations are labelled by their titles. Top right: current skeleton tree in circular layout, node color is the same as in galaxy map. Bottom left: current skeleton tree in ForceAtlas layout, node color is determined by paper knowledge temperature, with blue the coldest and red the hottest. Papers with more than 150 in-topic citations are labelled by title. Except the pioneering work, labelled nodes' size is amplified by 3 times. Bottom right: regional zoom of current skeleton tree in ForceAtlas layout.

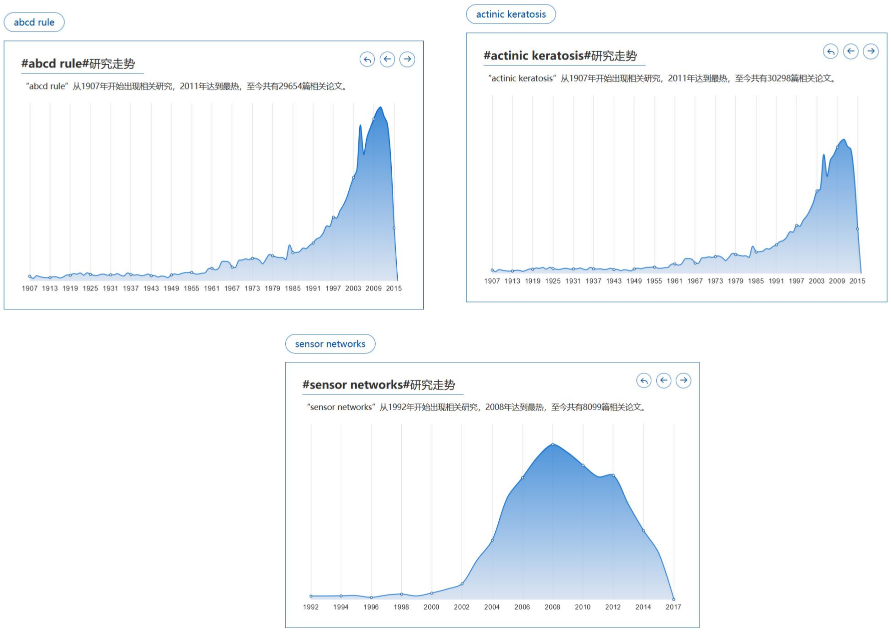

Fig S26. Coverage problems: Popularity trend of pioneering work's keywords provided by baidu research engine.

gain. To conclude, after a recent boom thanks to its popular child papers, the topic is now going downhill.

The skeleton tree is a bit special because it is made up of 2 parts. This is due to the separation of paper 'Connectionist language modeling for large vocabulary continuous speech recognition' (CLM) from the pioneering work, the only citation CLM has within the topic. In fact, CLM was published a bit earlier than the pioneering work, therefore its relation with the pioneering work may not be tight. This results in the edge cutting during skeleton tree extraction. CLM later inspired 'Efficient training of large neural networks for language modeling', whose work turned out to have a greater influence on the aforementioned popular child papers than that of the pioneering work. That is why skeleton tree finally takes a separated form.

Now we closely examine the current heat distribution with its latest skeleton tree (Fig. S29 bottom left). The pioneering work remains the only heat source in the topic and almost all of the most popular child papers have a knowledge temperature below average. Although they have indeed vitalized the topic, more importantly they themselves have proposed novel ideas that made them overshadow the pioneering work and become the new authorities in the domain (Fig. S29 galaxy map). The relatively loose connection to the core topic idea has resulted in their low knowledge temperature. Their "coolness" is also the reason that the cluster they are in is much colder than the one led by the pioneering work. Overall, we observe the general rule "the older the hotter" (Fig. 6(h)). The blue nodes that surround the pioneering work and popular child papers are papers with few or without any in-topic citations. The decrease of paper knowledge temperature is clear as we walk down the paths in skeleton tree. However, there are exceptions. Hit paper 'A unified architecture for natural language processing: deep neural networks with multitask learning' (UANLP) published in 2008 is colder than, for instance, its well-developed child 'Large Scale Distributed Deep Networks' published in 2012 and 'Parsing Natural Scenes and Natural Language with Recursive Neural Networks' published in 2011. These 2 child papers, represented as orange nodes, are relatively hot within the entire topic yet UANLP, depicted as a yellow node, has

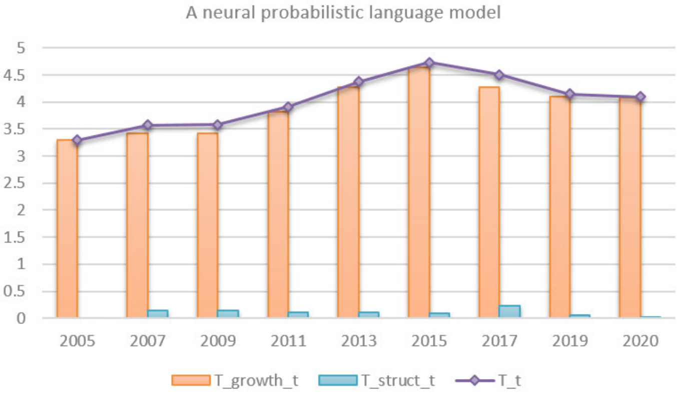

year	$\lvert V^t \rvert$	$\lvert E^t \rvert$	n_t	V_t	$UsefulInfo^t$	T^t_{growth}	T^t_{struct}	T^t
2005	25	53	16.204	25	8.796	3.297		3.297
2007	61	172	38.068	61	22.932	3.424	0.151	3.575
2009	103	305	64.249	103	38.751	3.426	0.151	3.577
2011	173	649	96.959	173	76.041	3.813	0.101	3.914
2013	341	1852	170.71	341	170.29	4.269	0.101	4.37
2015	1050	8050	483.016	1050	566.984	4.646	0.085	4.731
2017	2179	15992	1090.162	2179	1088.838	4.271	0.229	4.5
2019	3157	22213	1648.768	3157	1508.232	4.092	0.053	4.145
2020	3265	22912	1711.825	3265	1553.175	4.076	0.015	4.091

Fig S27. Neural language model: topic statistics and knowledge temperature evolution

only an average knowledge temperature. Their temperature difference lies mainly in their research focus reflected by their citation patterns. Although these 2 child papers both have a few followers in the latest skeleton tree, they are still less popular than their parent in terms of idea diffusion. This counter example also illustrates that the general rules "the more influential the hotter" is very weak in the topic (Fig. S63(h)).

We observe in addition certain clustering effect in the skeleton tree (Table S7). For example, all child papers of 'Road2Vec: Measuring Traffic Interactions in Urban Road System from Massive Travel Routes' have a research interest related to geographic relation. This confirms the effectiveness of our skeleton tree extraction algorithm. In addition, this is a newly emerged direction, hence their research interest may be among the latest trends.

When comparing T^t's evolution with the keyword frequency of the pioneering work queried from Baidu Xueshu, we find the evolution of topic knowledge temperature is similar to the keyword occurence trend until 2015. After 2009, keyword 'distributed representation' is the "hottest" in 2014 (Fig. S30). Another keyword, 'statistical language modelling' experiences popularity fluctuations from 2010 to 2014. Its average popularity over this period drops slightly. is the "hottest" during 2007 and 2010. Indeed, we observe an increasing topic knowledge temperature until 2015 and T^t reaches its peak in 2015 (Fig. S27).

S2.2.6 A unified architecture for natural language processing: deep neural networks with multitask learning

As is shown by T^t_{growth} and T^t, the topic continuously gained fame between 2009 and 2015 (Fig. S31). Almost all of its most influential child papers were published during this period. Their own research content and their attractiveness to subsequent works

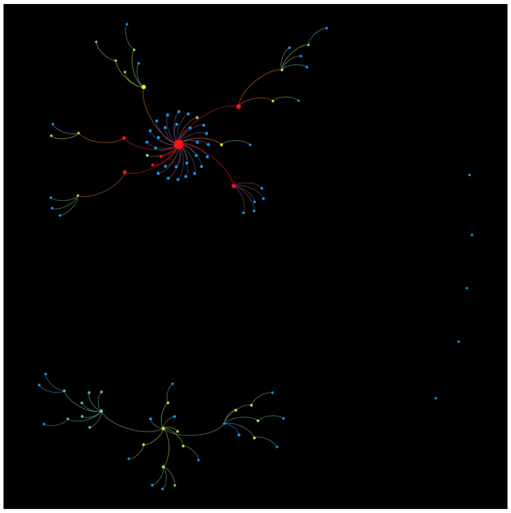

(a) Skeleton tree until 2009

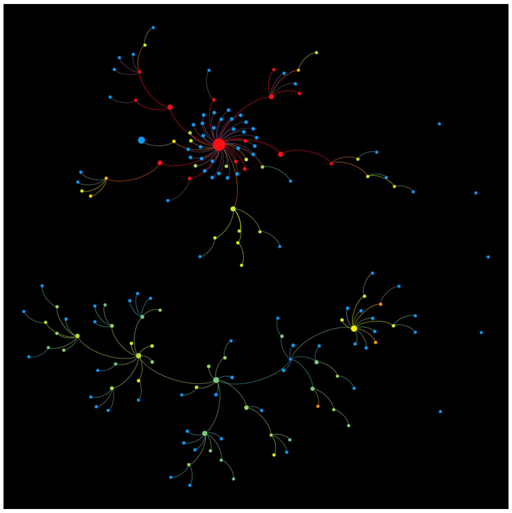

(b) Skeleton tree until 2011

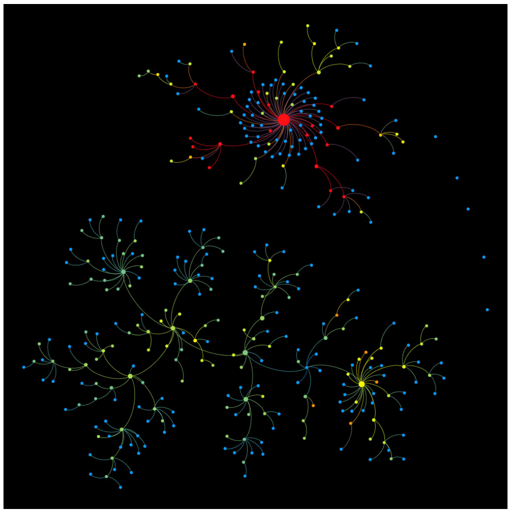

(c) Skeleton tree until 2013

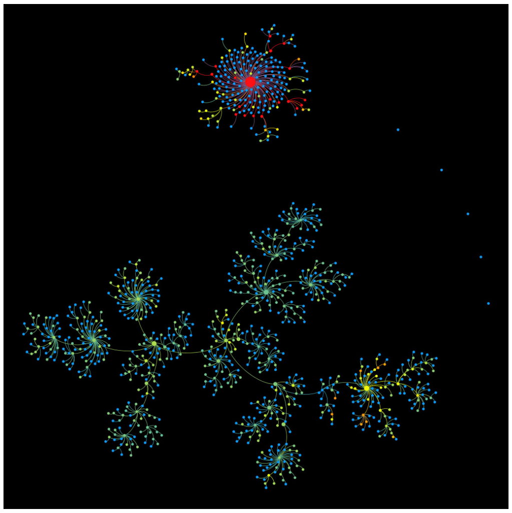

(d) Skeleton tree until 2015

Fig S28. Neural language model: Skeleton tree evolution

title	year
Road2Vec: Measuring Traffic Interactions in Urban Road System from Massive Travel Routes	2017
Knowledge Embedding with Geospatial Distance Restriction for Geographic Knowledge Graph Completion	2019
A regionalization method for clustering and partitioning based on trajectories from NLP perspective	2019
From Motion Activity to Geo-Embeddings: Generating and Exploring Vector Representations of Locations, Traces and Visitors through Large-Scale Mobility Data	2019
Detecting geo-relation phrases from web texts for triplet extraction of geographic knowledge: a context-enhanced method	2019

Table S7. Neural language model: Clustering effect example. First line is the parent paper and the rest children.

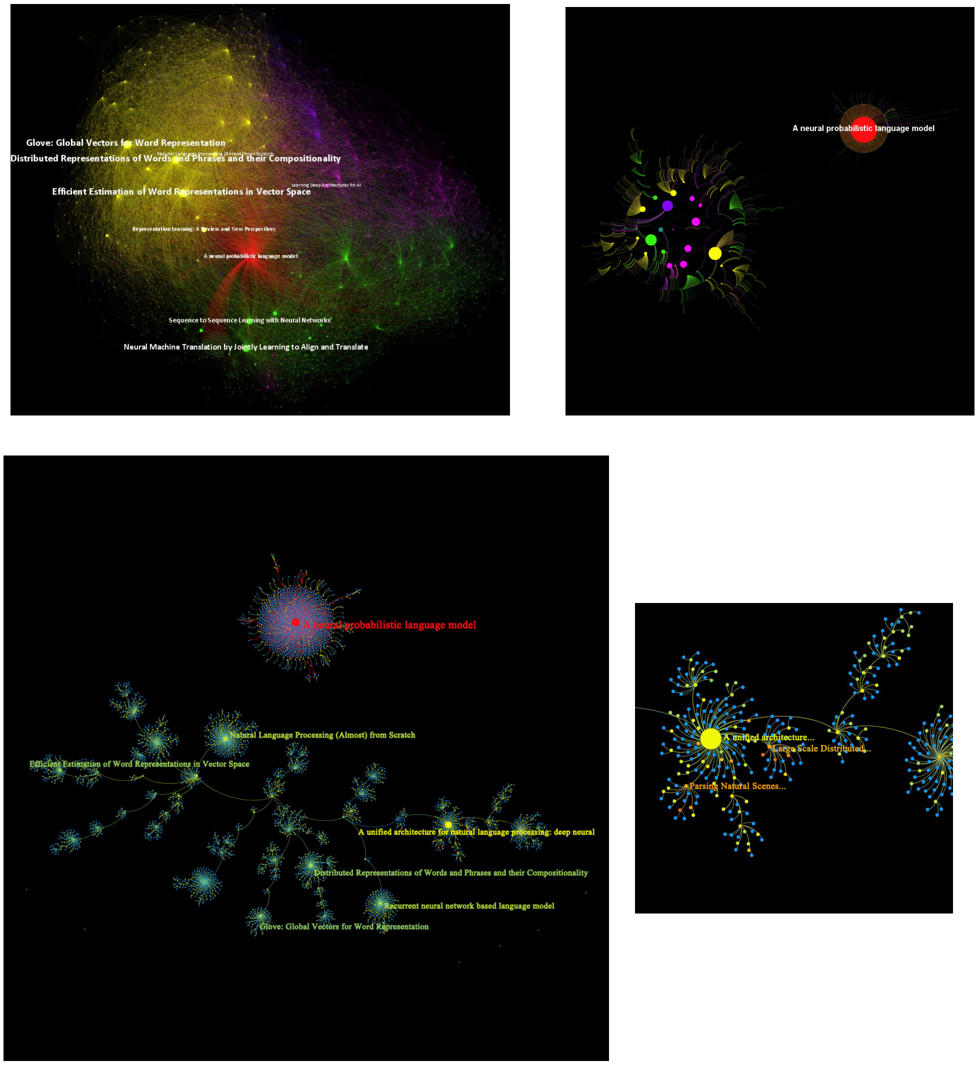

Fig S29. Neural language model: Galaxy map, current skeleton tree and its regional zoom. Top left: current galaxy map. Papers with the most total citations are labelled by their titles. Top right: current skeleton tree in circular layout, node color is the same as in galaxy map. Bottom left: current skeleton tree in ForceAtlas layout, node color is determined by paper knowledge temperature, with blue the coldest and red the hottest. Papers with more than 600 in-topic citations are labelled by title. Except the pioneering work, labelled nodes' size is amplified by 3 times. Bottom right: regional zoom of current skeleton tree in ForceAtlas layout.

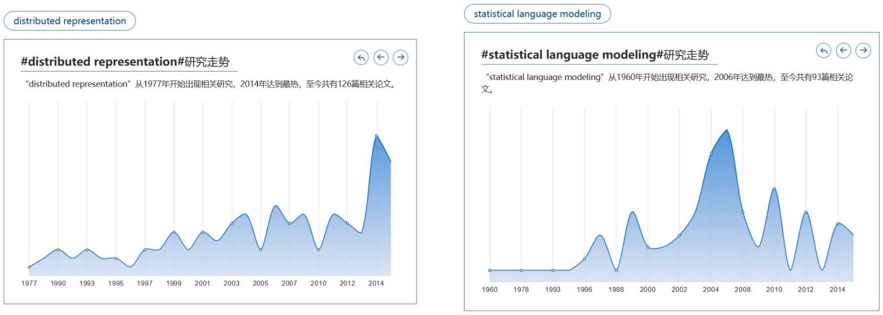

Fig S30. Neural language model: Popularity trend of pioneering work's keywords provided by baidu research engine.

have contributed to a rapid increase in topic's useful information during 2013 and 2015 (Fig. 5(i)). After that, despite a steady size growth, the topic has gradually cooled down. This is because the majority of prominent child papers, namely 'Efficient Estimation of Word Representations in Vector Space' (EEWRVS), 'Distributed Representations of Words and Phrases and their Compositionality' (DRWPC) and 'Word Representations: A Simple and General Method for Semi-Supervised Learning' (WRSSL), were published no later than 2013. They brought large amounts of new knowledge and, more importantly, attracted much immediate attention after their publication. By the end of 2015, these child papers, having collected a fair share of in-topic citations, had already become crucial members of the topic. Together with the pioneering work, they shaped topic knowledge (Fig. S32(d)). Child papers published no earlier than 2016 enriched the ideas proposed by the aforementioned popular child papers (Fig. S32(e,f)). Very few have had a significant subsequent development even though the topic has succeeded in attracting a stable stream of recent attention. Therefore, the enrichment of knowledge base has slowed down and thus the knowledge temperature has slightly dropped. To sum up, the topic demonstrates a rise-then-fall dynamics.

The skeleton tree of this topic manifests a gradual structural advancement in line with a constantly small $T^t_{structure}$ (Fig. S32). Its popular child papers have unanimously dedicated themselves to one single research sub-direction, which is portrayed by the steadily-growing big branch (Fig. S33 bottom left).

Now we closely examine the internal heat distribution and its latest skeleton tree (Fig. S33 bottom left). The pioneering work is the only heat source. Interestingly, half of the most popular child papers have a knowledge temperature below average. In fact, they all cited another popular child paper, WRSSL. In terms of idea inheritance, they are less close to the pioneering work than WRSSL. A bigger portion of original idea has caused their relatively low knowledge temperature. We see a clear paper knowledge temperature decline from the root to leaves. This corresponds with the general rule "the older the hotter" (Fig. 6(i)). As the topic contains 2 articles published earlier than the pioneering work and they have few in-topic citations, the average node knowledge temperature for the oldest papers is not maximal. In addition, the blue nodes that surround the pioneering work and the most popular child papers are papers with few or without any in-topic citations. However, even if we set aside the oldest papers and the aforementioned coldest papers, the general rule is violated. Hit paper 'Learning Deep Architectures for AI' (LDAAI) published in 2009 is colder than, for instance, its child papers '3D Convolutional Neural Networks for Human Action Recognition' published in 2013 and 'Learning structured embeddings of knowledge bases' published in 2011. These 2 child papers are rather hot yet LDAAI only has an average knowledge temperature. This is mainly due to their relatively different research focus as their in-topic citations

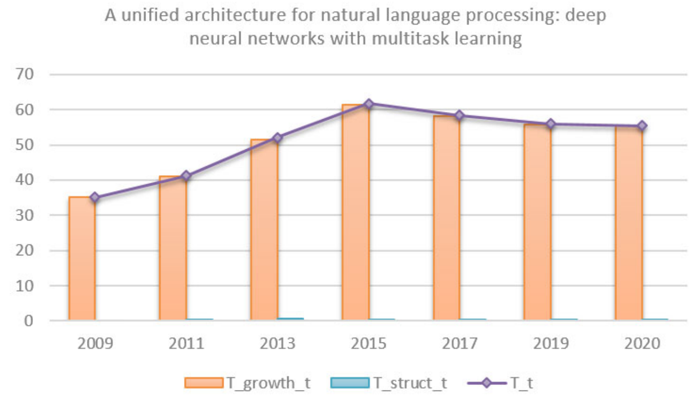

year	$\|V^t\|$	$\|E^t\|$	n_t	V_t	$UsefulInfo^t$	T^t_{growth}	T^t_{struct}	T^t
2009	24	36	20.883	24	3.117	35.066		35.066
2011	113	236	83.927	113	29.073	41.081	0.117	41.199
2013	291	1021	172.368	291	118.632	51.511	0.532	52.043
2015	889	4818	441.78	889	447.22	61.399	0.39	61.79
2017	1766	9451	926.156	1766	839.844	58.18	0.178	58.358
2019	2640	13483	1441.288	2640	1198.712	55.888	0.087	55.976
2020	2733	13855	1503.842	2733	1229.158	55.45	0.01	55.46

Fig S31. A unified architecture for NLP: topic statistics and knowledge temperature evolution

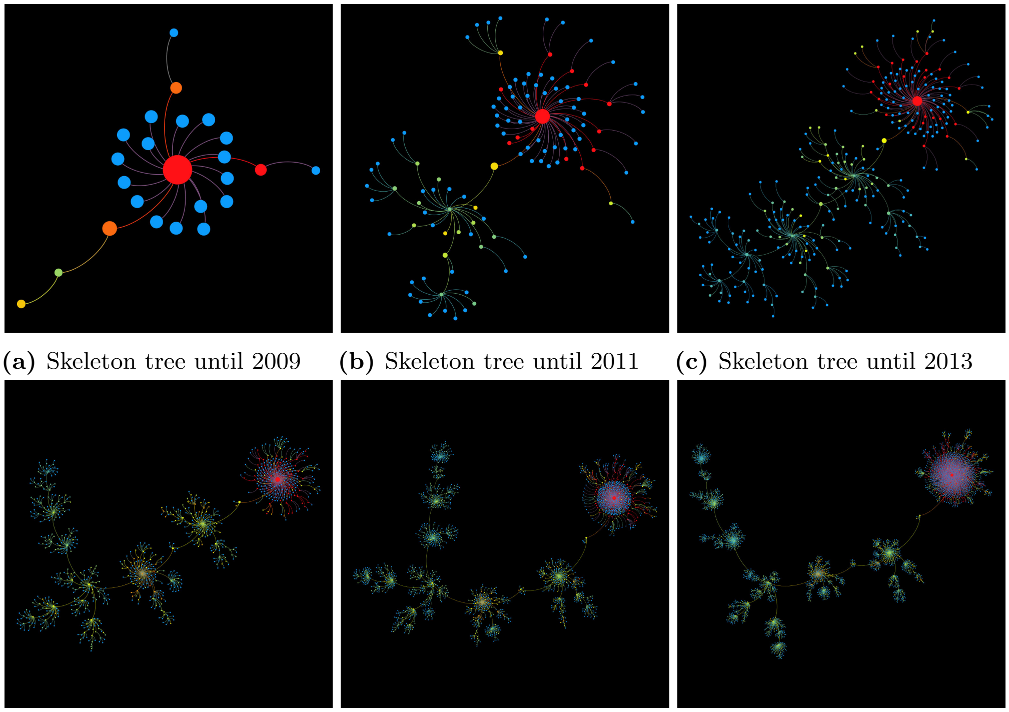

(a) Skeleton tree until 2009 **(b)** Skeleton tree until 2011 **(c)** Skeleton tree until 2013

(d) Skeleton tree until 2015 **(e)** Skeleton tree until 2017 **(f)** Skeleton tree until 2019

Fig S32. A unified architecture for NLP: Skeleton tree evolution

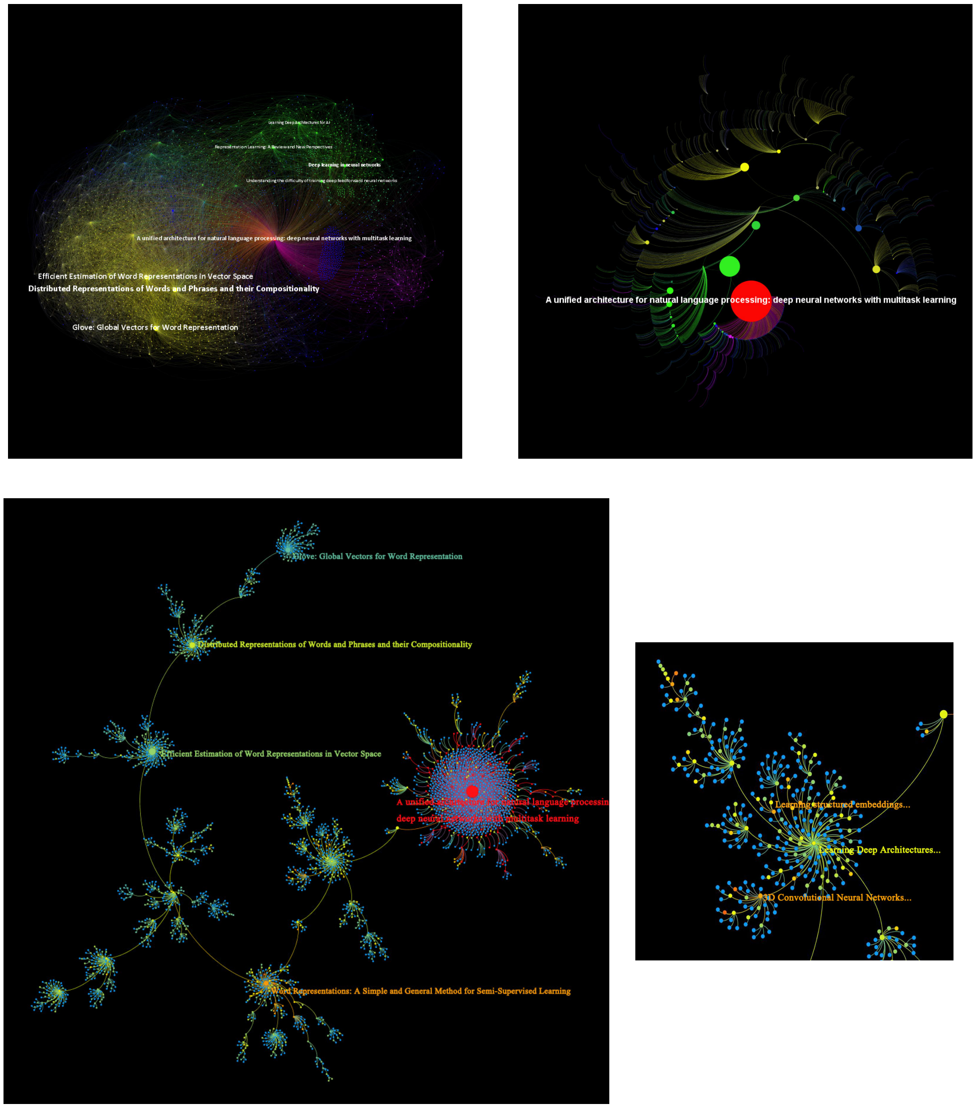

Fig S33. A unified architecture for NLP: Galaxy map, current skeleton tree and its regional zoom. Top left: current galaxy map. Papers with the most total citations are labelled by their titles. Top right: current skeleton tree in circular layout, node color is the same as in galaxy map. Bottom left: current skeleton tree in ForceAtlas layout, node color is determined by paper knowledge temperature, with blue the coldest and red the hottest. Papers with more than 400 in-topic citations are labelled by title. Except the pioneering work, labelled nodes' size is amplified by 3 times. Bottom right: regional zoom of current skeleton tree in ForceAtlas layout.

title	year
Throughput-Optimized OpenCL-based FPGA Accelerator for Large-Scale Convolutional Neural Networks	2016
Automatic code generation of convolutional neural networks in FPGA implementation	2016
Throughput-Optimized FPGA Accelerator for Deep Convolutional Neural Networks	2017
Escher: A CNN Accelerator with Flexible Buffering to Minimize Off-Chip Transfer	2017
Towards Efficient Hardware Acceleration of Deep Neural Networks on FPGA	2018
UniCNN: A Pipelined Accelerator Towards Uniformed Computing for CNNs	2018

Table S8. A unified architecture for NLP: Clustering effect example. First line is the parent paper and the rest children.

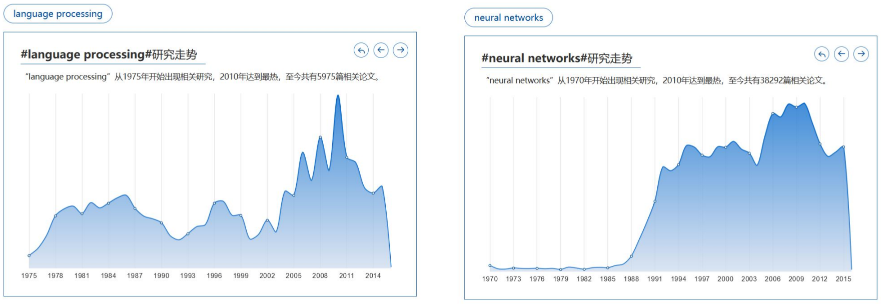

Fig S34. A unified architecture for NLP: Popularity trend of pioneering work's keywords provided by baidu research engine.

do not overlap with one another. Similarly, popular child paper EEWRVS is slightly colder than its descendant, DRWPC. These counter examples also illustrate that the general rule "the more influential the hotter" is very weak in this topic (Fig. S63(i)).

We observe in addition certain clustering effect in the skeleton tree (Table S8). For example, all child papers of 'Throughput-Optimized OpenCL-based FPGA Accelerator for Large-Scale Convolutional Neural Networks' have a research interest towards accelerator. This confirms the effectiveness of our skeleton tree extraction algorithm.

When comparing T^t's evolution with the keyword frequency of the pioneering work queried from Baidu Xueshu, we find the evolution of topic knowledge temperature is similar to the keyword occurence trend until 2016. After 2009, both keywords 'language processing' and 'neural networks' are the "hottest" in 2010 (Fig. S34). Both keywords regain some of their popularity around 2015 after a slight slip. As the topic was founded in 2008, it was still in its early development in 2010 and its T^t grew steadily up at that time. Afterwards, the topic indeed became hotter until 2015 and its topic knowledge temperature reached its peak in 2015 (Fig. S31).

S2.2.7 Bose-Einstein condensation in a gas of sodium atoms

Founded in 1995, this topic thrived for some 20 years before starting to stagnate since 2013 (Fig. S35). While most of the highest-cited child papers within the topic came between 1997 and 2003, several came after 2006, namely 'Bose-Einstein condensation of exciton polaritons' (BECEP) published in 2006 in Nature, 'Production of Cold Molecules via Magnetically Tunable Feshbach Resonances' published in 2006 in Reviews of Modern Physics, and 'Bose-Einstein condensation of photons in an optical microcavity' (BECPOM) published in 2010 in Nature. The relay among these popular

child papers maintained the topic's flourishing for 20 years. In addition, the topic was most prolific between 2010 and 2012, with annual publication number all exceeding 5% of current topic size. It is also during these 2 years that the topic collected useful information much faster than any other period (Fig. 5(j)). The increasing inflow of knowledge, together with the exposure brought by the aforementioned popular child papers, contributed to a slightly bigger climb in T^t and T^t_{growth} between 2011 and 2013. After that, the topic has not so far welcomed any superstars that have incited remarkable development. Yet it still has a rather stable knowledge accumulation judging from basic statistics. Hence overall T^t_{growth} ceased to go up and so is T^t.

$T^t_{structure}$ is higher in early days, which corresponds with a multi-dimensional growth in skeleton tree thanks to influential child papers published around 2000 (Fig. S36). After 2013, skeleton tree has fixed its structure. We observe few visible changes in skeleton tree, namely some development in the research direction jointly led by popular child papers BECEP and BECPOM and a new small research branch deriving from the school of thought led by child papers 'Second-Order Corrections to Mean Field Evolution of Weakly Interacting Bosons. I.' published in 2010 and its rather successful descendant 'Derivation of the Cubic NLS and Gross-Pitaevskii Hierarchy from Manybody Dynamics in d = 3 Based on Spacetime Norms' published in 2014.

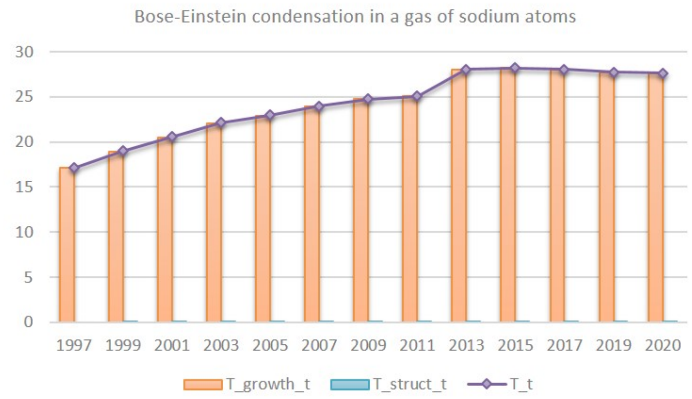

year	$\|V^t\|$	$\|E^t\|$	n_t	V_t	$UsefulInfo^t$	T^t_{growth}	T^t_{struct}	T^t
1997	89	139	71.133	89	17.867	17.114		17.114
1999	228	445	164.84	228	63.16	18.92	0.056	18.976
2001	399	933	265.993	399	133.007	20.518	0.048	20.566
2003	596	1600	369.024	596	226.976	22.092	0.036	22.127
2005	784	2351	467.427	784	316.573	22.942	0.024	22.966
2007	1014	3395	579.603	1014	434.397	23.93	0.024	23.954
2009	1213	4326	670.455	1213	542.546	24.747	0.017	24.764
2011	1454	5310	794.184	1454	659.816	25.043	0.016	25.059
2013	1708	6450	833.549	1708	874.451	28.028	0.013	28.041
2015	1905	7430	924.707	1905	980.293	28.179	0.012	28.191
2017	2066	8141	1009.101	2066	1056.899	28.005	0.021	28.026
2019	2296	9013	1132.976	2296	1163.024	27.72	0.021	27.741
2020	2338	9171	1157.694	2338	1180.306	27.624	0.005	27.629

Fig S35. Bose-Einstein condensation: topic statistics and knowledge temperature evolution

Now we closely examine its internal heat distribution together with its latest skeleton tree (Fig. S37 bottom left). After more than 20 years of development, the heat has fully propagated to recent research directions led by popular child papers. Popular

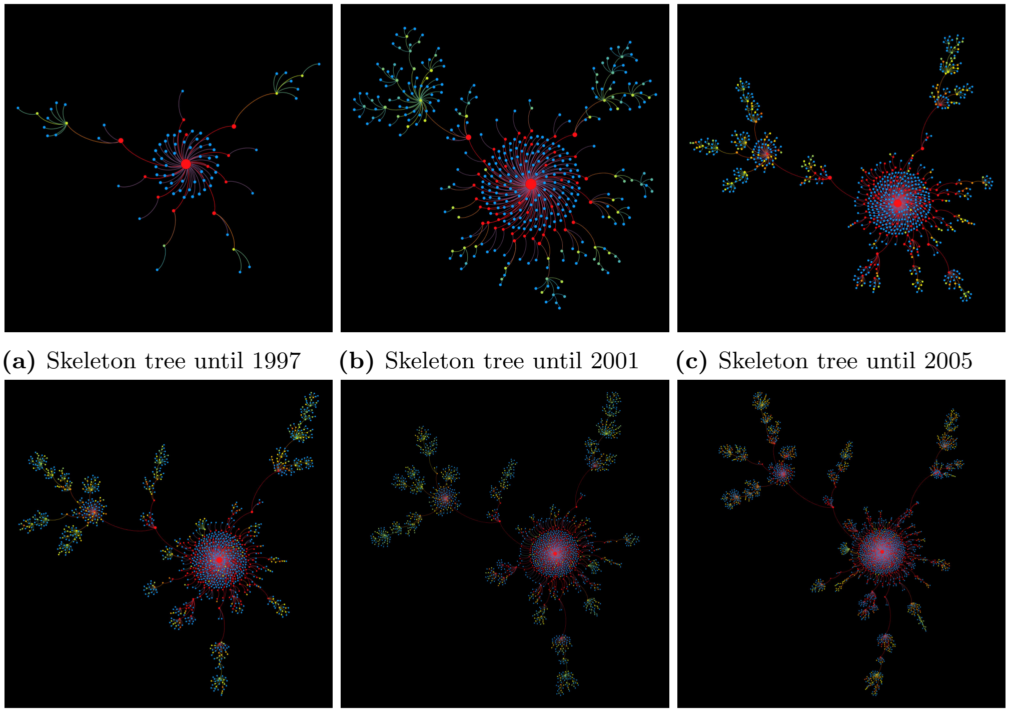

(a) Skeleton tree until 1997 **(b)** Skeleton tree until 2001 **(c)** Skeleton tree until 2005

(d) Skeleton tree until 2009 **(e)** Skeleton tree until 2013 **(f)** Skeleton tree until 2017

Fig S36. Bose-Einstein condensation: Skeleton tree evolution

child papers are among the hottest articles and the child papers published during the flourishing period are relatively hot in general (Fig. 6(j)). The knowledge temperature decrease from cores to ends is clear. This corresponds with the general rule "the older the hotter". The blue nodes that surround the pioneering work and popular child papers in main clusters are papers with few or without any in-topic citations. However, there are exceptions. Paper 'A gapless theory of Bose-Einstein condensation in dilute gases at finite temperature' published in 2000 is colder than its child paper 'Theory of the weakly interacting Bose gas' (TWIBS) published in 2004. TWIBS is also slightly colder than its direct child paper in current skeleton tree 'Weakly-Interacting Bosons in a Trap within Approximate Second Quantization Approach' (WIBTASQ) published in 2007. This is mainly due to their relatively different research focus as most of their in-topic citations do not overlap with one another. As WIBTASQ is the least developed among the three in terms of citations, this counter examples also illustrates that the general rule "the more influential the hotter" is weak (Fig. S63(j)).

We find the knowledge temperature evolution of child paper BECEP particularly interesting. Despite topic's stagnation starting from around 2013 and 2014, its knowledge temperature has been constantly on the rise since its publication, from 60.4 in 2006 to 83.5 in 2020. Its rising temperature demonstrates its above-average recent development compared to the entire topic.

We observe in addition certain clustering effect in the skeleton tree (Table S9). For example, all child papers of 'Comparative analysis of electric field influence on the quantum wells with different boundary conditions: II. Thermodynamic properties' have a research interest towards thermodynamics. This confirms the effectiveness of our skeleton tree extraction algorithm.

When comparing T^t's evolution with the keyword frequency of the pioneering work queried from Baidu Xueshu, we find the evolution of topic knowledge temperature is

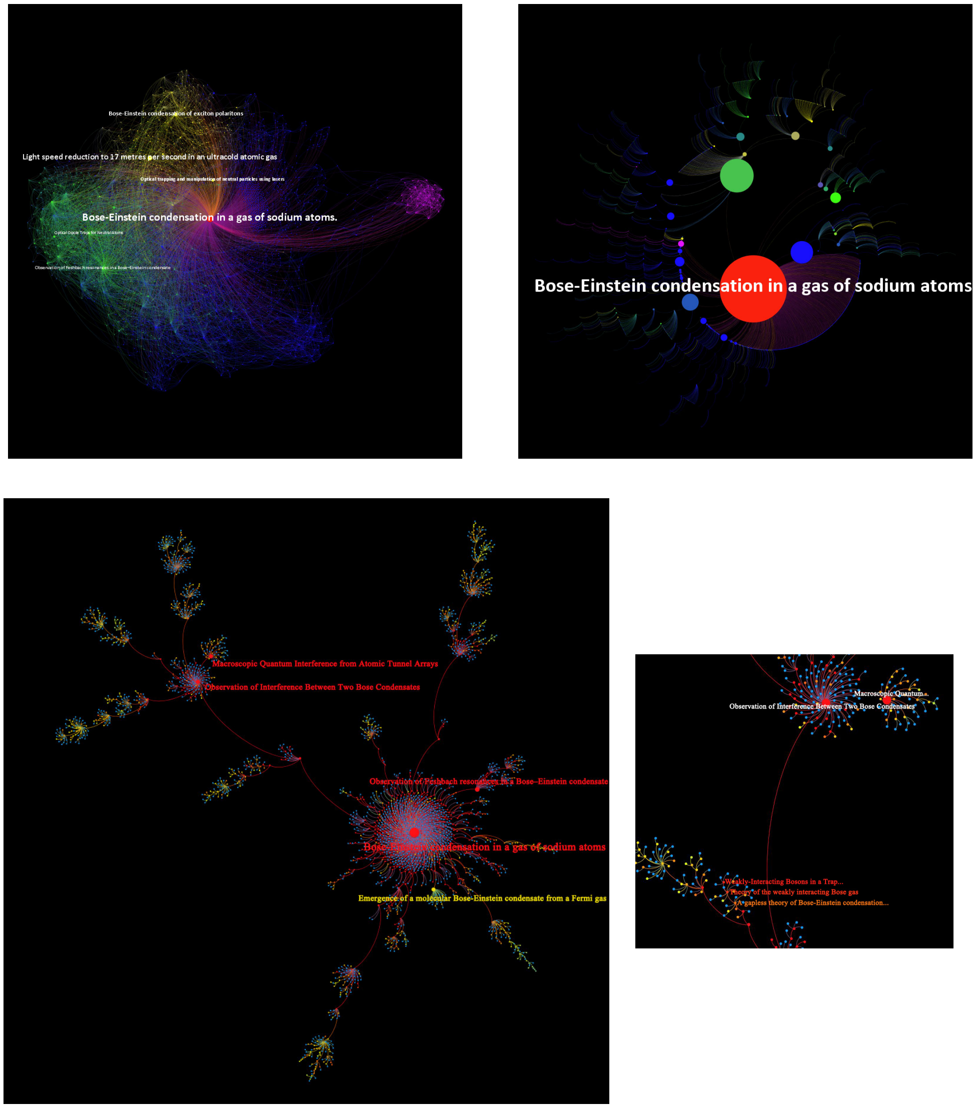

Fig S37. Bose-Einstein condensation: Galaxy map, current skeleton tree and its regional zoom. Top left: current galaxy map. Papers with the most total citations are labelled by their titles. Top right: current skeleton tree in circular layout, node color is the same as in galaxy map. Bottom left: current skeleton tree in ForceAtlas layout, node color is determined by paper knowledge temperature, with blue the coldest and red the hottest. Papers with more than 150 in-topic citations are labelled by title. Except the pioneering work, labelled nodes' size is amplified by 3 times. Bottom right: regional zoom of current skeleton tree in ForceAtlas layout.

title	year
Comparative analysis of electric field influence on the quantum wells with different boundary conditions: II. Thermodynamic properties	2015
Theory of the Robin quantum wall in a linear potential. II. Thermodynamic properties	2016
Comparative analysis of electric field influence on the quantum wells with different boundary conditions.: I. Energy spectrum, quantum information entropy and polarization	2015
Thermodynamic Properties of the 1D Robin Quantum Well	2018

Table S9. Bose-Einstein condensation: Clustering effect example. First line is the parent paper and the rest children.

similar to the keyword occurence trend until 2015. Since 1998, four keywords of the pioneering work, ‘atom lasers’, ‘atomic beams’, ‘gas lasers’ and ‘laser theory’, are “hottest” between 2005 and 2007 (Fig. S38). This observation corresponds with a moderate acceleration in useful information accumulation during the same period. Another keyword ‘bose-einstein condensation’ is most trendy during 2006 and 2010, and the latest keyword, ‘electronics cooling’, has continued to gain popularity ever since 2005. Furthermore, ‘electronics cooling’ experienced a significant uprise in fame in 2013. The steady increase in topic knowledge temperature until 2011 corresponds with the peak-period relay of different keywords. In particular, we observe that the topic indeed gained more useful information around 2007 compared to other periods before 2011 (Fig. 5(j)). The non-trivial T^t’s upsurge, as well as the spike in useful information accumulation between 2011 and 2013, fits the emergence of field ‘electronics cooling’.

S2.3 Awakened topics

S2.3.1 Long short-term memory

After a boom right after its birth, the topic hibernated for as long as 10 years before having an explosive growth. As is shown by the basic statistics, the topic’s expansion in the first 15 years is much slower than recently. Apart from publication quantity difference, we also observe an obvious discrepancy in article’s contribution to topic’s flourishing. Few child papers turned out to be popular among topic members. Child paper ‘Learning to Forget: Continual Prediction with LSTM’ (LFCP) published in 2000 is the only superstar the topic had for a long time. It successfully extended the pioneering work’s idea and founded a new research focus, represented by the branch pointing to the bottom-left in skeleton tree (Fig. S40(b,c,d)). Although the research branch seemed small by 2001, it already meant something compared to the then topic size. The evolution in knowledge structure led to a high $T^t_{structure}$. The remaining popular child papers, namely 2 published in 2003, ‘Kalman filters improve LSTM network performance in problems unsolvable by traditional recurrent nets’ and ‘Learning precise timing with lstm recurrent networks’, arriving later unanimously focused on LFCP’s idea. Together they contributed to the maturation of this new sub-field and maintained partly the heat-level of the entire topic. The situation changed after 2010. The artificial intelligence frenzy pulled the topic under the spotlight. Thanks to the favorable background, the topic welcomed numerous popular child papers during 2013 and 2016, for instance, ‘Sequence to Sequence Learning with Neural Networks’ (S2SNN) ,‘Neural Machine Translation by Jointly Learning to Align and Translate’ (NMTAT) and ‘Deep Residual Learning for Image Recognition’ (DRLIR). While inheriting the essence of LFCP, they brought alone considerable amount of new knowledge, introduced new sub-topics and produced the renaissance of this old topic

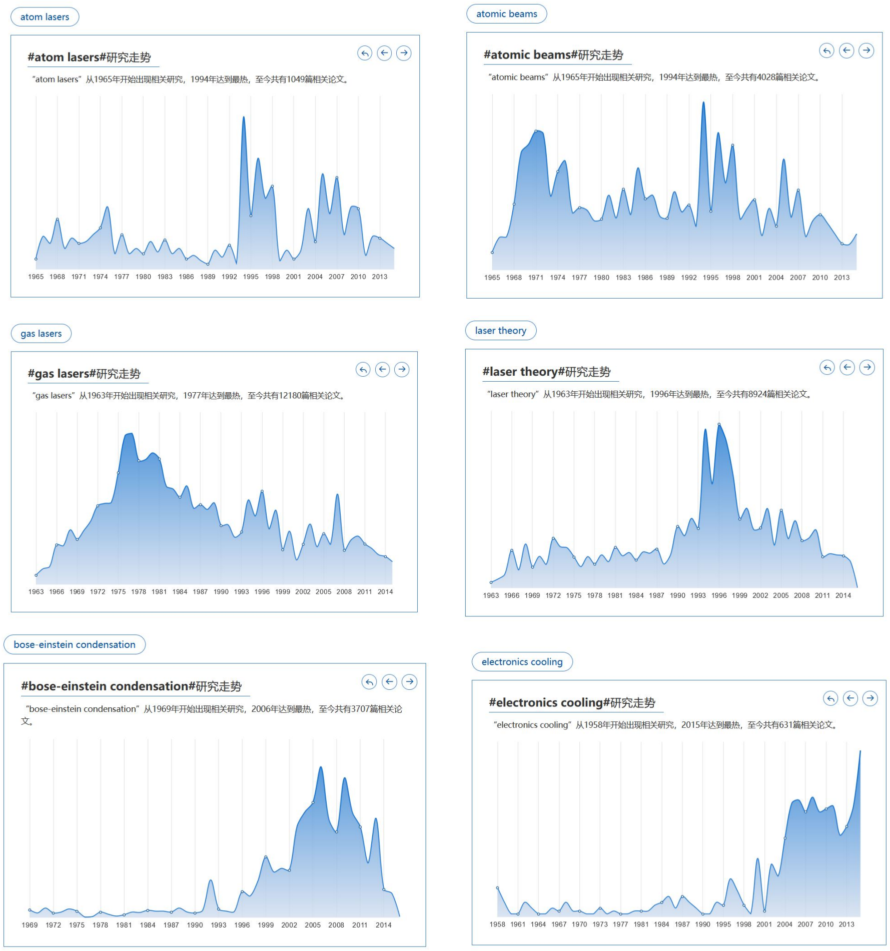

Fig S38. Bose-Einstein condensation: Popularity trend of pioneering work's keywords provided by baidu research engine.

(Fig. 5(k), Fig. S41, S40(d,e,f)). Consequently, we see a slightly higher $T^t_{structure}$ around 2015 owing to the knowledge structure enrichment and a soar in T^t starting from 2017. The long interval between the birth and the peak of impact and popularity makes us define this research field as an awakened topic.

There is a tiny cluster isolated from the majority of the skeleton tree (Fig. S41 in the top-middle of current skeleton tree). This is because the topic contains several child papers published at the same time or evenly a bit earlier than the pioneering work. Comparatively speaking, their work is not very intimately related to that of the pioneering article. Therefore, altogether with some of their closest descendants, they were disconnected from the pioneering work during the skeleton tree construction.

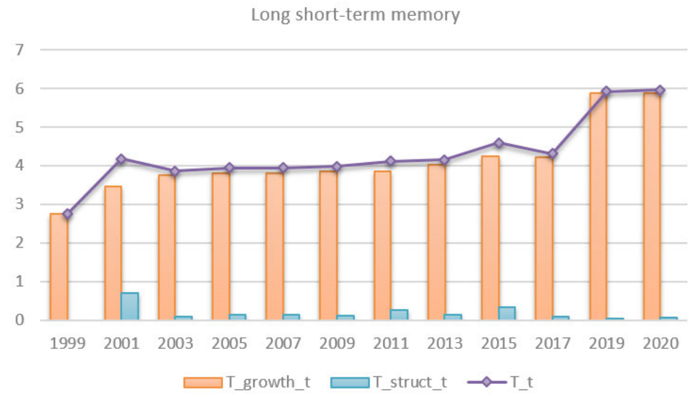

year	$\lvert V^t \rvert$	$\lvert E^t \rvert$	n_t	V_t	$UsefulInfo^t$	T^t_{growth}	T^t_{struct}	T^t
1999	17	21	14.333	17	2.667	2.747		2.747
2001	56	109	37.44	56	18.56	3.465	0.714	4.179
2003	102	237	62.773	102	39.227	3.764	0.1	3.864
2005	156	407	95.061	156	60.939	3.801	0.143	3.944
2007	230	682	140.092	230	89.908	3.803	0.139	3.942
2009	331	1010	198.412	331	132.588	3.864	0.121	3.985
2011	422	1414	253.302	422	168.698	3.859	0.261	4.12
2013	568	2129	326.996	568	241.004	4.024	0.133	4.156
2015	1323	7166	722.591	1323	600.409	4.241	0.348	4.589
2017	5912	35684	3239.903	5912	2672.097	4.227	0.09	4.316
2019	15279	90463	6023.461	15279	9255.539	5.876	0.046	5.921
2020	16777	98553	6610.64	16777	10166.36	5.879	0.075	5.954

Fig S39. Long short-term memory: topic statistics and knowledge temperature evolution

Now we examine the heat distribution within the topic (Fig. S41 bottom). The pioneering work remains the only heat source so far. Although this topic has a long history, its flourishing took place a few years ago. It needs more time to have a thorough heat diffusion within the topic. That is why most popular child papers have a paper knowledge temperature around or a bit above average. At present, most of the hottest articles are located around the pioneering work the central cluster. The knowledge temperature decline from the core to ends is obvious. This corresponds with the general rule “the older the hotter” (Fig. 6(k)). Note that the blue nodes surrounding the pioneering work and popular child papers in non-trivial clusters are papers with few or without any in-topic citations. The low average temperature for the oldest papers is due to their loose connection to the topic majority as they were published no later than the pioneering work and have had few child papers within the

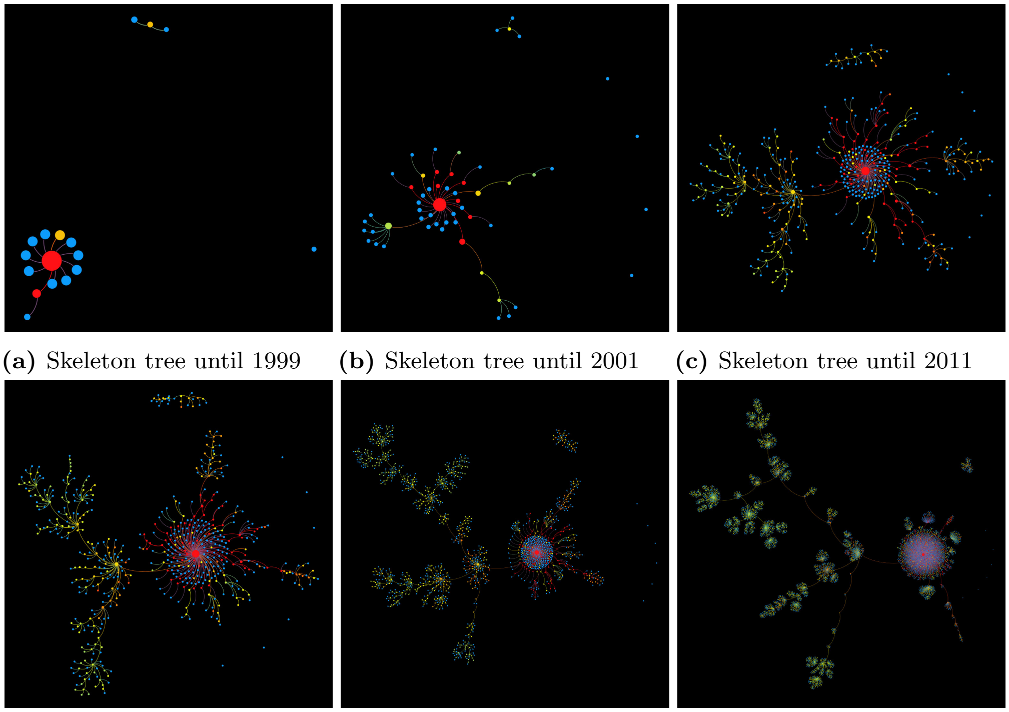

(a) Skeleton tree until 1999 **(b)** Skeleton tree until 2001 **(c)** Skeleton tree until 2011

(d) Skeleton tree until 2013 **(e)** Skeleton tree until 2015 **(f)** Skeleton tree until 2017

Fig S40. Long short-term memory: Skeleton tree evolution

topic. However, even if we let alone these papers, age is not guarantee of a bigger impact and popularity. For instance, 2 popular children papers of LFCP are slightly hotter than itself. They are 'Kalman filters improve LSTM network performance in problems unsolvable by traditional recurrent nets' published in 2003 and 'Modeling systems with internal state using evolino' published in 2005. Both have a rather high paper knowledge temperature. Similarly, article 'Generating Text with Recurrent Neural Networks' published in 2011 is also slightly colder than its child, 'Understanding the exploding gradient problem', which was published in 2012. Their temperature difference is mainly owing to their research focus, as is reflected by their distinct citation patterns. These counter examples also illustrate that the general rule "the more influential the hotter" is weak (Fig. S63(k)).

We find the knowledge temperature evolution of LFCP particularly interesting. Its knowledge temperature dropped from 6.53 to 5.08 from 2001 to 2005. The decrease rate is greater than that of topic knowledge temperature. This is because its followers had little development, thus overall the bundle led by Learning to forget had a slower development than the entire topic. Its temperature has been on the rise since 2007. In particular, the increase has greatly accelerated from 2015. We attribute its surge to the arrival of several popular child papers published between 2014 and 2016: S2SNN (2014), 'Empirical Evaluation of Gated Recurrent Neural Networks on Sequence Modeling' (2014), NMTAT (2015) and DRLIR (2016) (Fig. S41). Their instantaneous popularity has brought learning to forget back to scientists' attention. Recall that these papers also contributed a lot to the knowledge temperature leap of the entire topic starting from 2017.

We observe in addition certain clustering effect in the skeleton tree. For example, almost all child papers of 'Developing a Long Short-Term Memory (LSTM) based model for predicting water table depth in agricultural areas' deal with earth science and

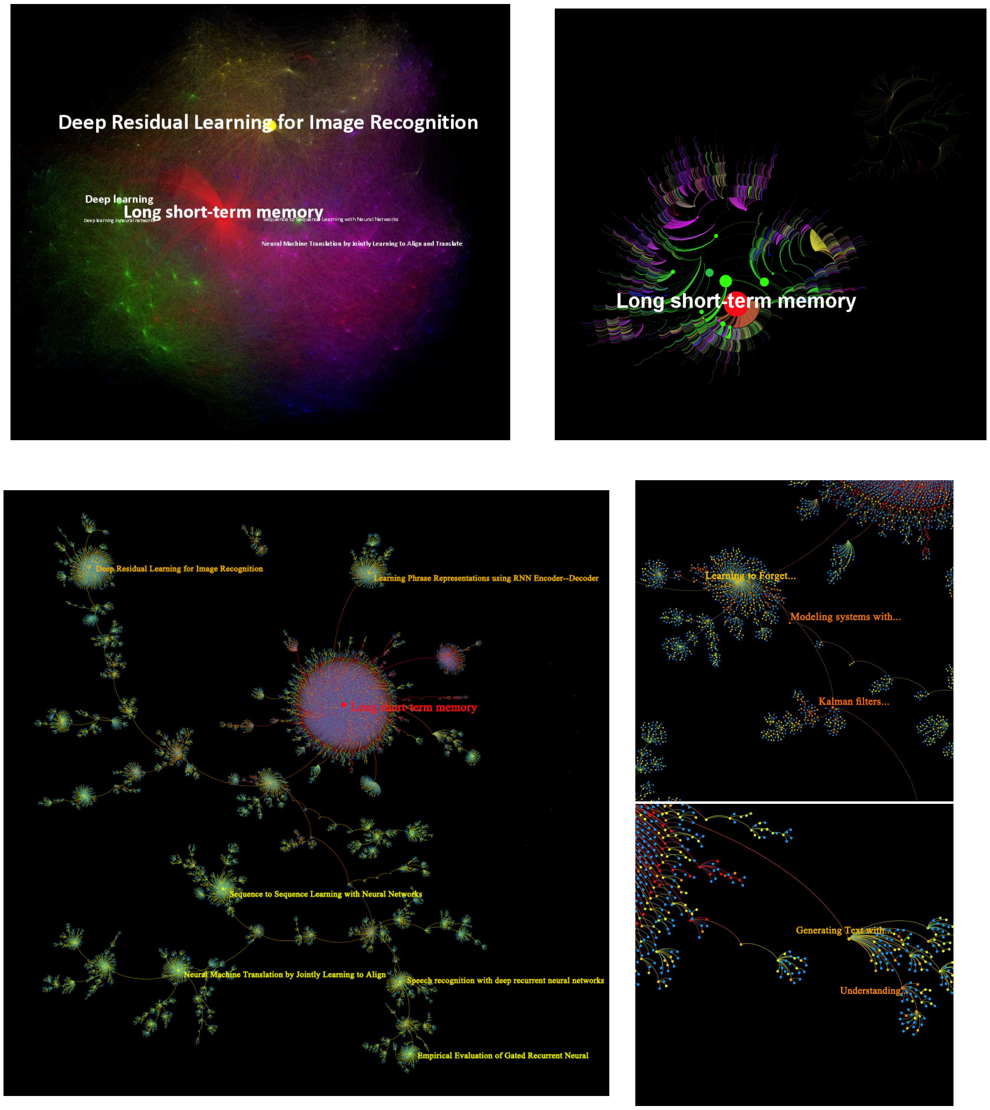

Fig S41. Long short-term memory: Galaxy map, current skeleton tree and its regional zoom. Top left: current galaxy map. Papers with the most total citations are labelled by their titles. Top right: current skeleton tree in circular layout, node color is the same as in galaxy map. Bottom left: current skeleton tree in ForceAtlas layout, node color is determined by paper knowledge temperature, with blue the coldest and red the hottest. Papers with more than 1000 in-topic citations are labelled by title. Except the pioneering work, labelled nodes' size is amplified by 3 times. Bottom right: 2 regional zooms of current skeleton tree in ForceAtlas layout.

title	year
Developing a Long Short-Term Memory (LSTM) based model for predicting water table depth in agricultural areas	2018
Stream-Flow Forecasting of Small Rivers Based on LSTM	2020
Developing a Long Short-Term Memory-based signal processing method for Coriolis mass flowmeter	2019
Direct Multistep Wind Speed Forecasting Using LSTM Neural Network Combining EEMD and Fuzzy Entropy	2019
Dynamic neural network modelling of soil moisture content for predictive irrigation scheduling	2018
SMArtCast: Predicting soil moisture interpolations into the future using Earth observation data in a deep learning framework	2020
Short-Term Streamflow Forecasting for Paraíba do Sul River Using Deep Learning	2019
Synthetic well logs generation via Recurrent Neural Networks	2018
Reservoir Facies Classification using Convolutional Neural Networks	2019
Comparative applications of data-driven models representing water table fluctuations	2019

title	year
FiLM: Visual Reasoning with a General Conditioning Layer	2018
LEARNING TO COLOR FROM LANGUAGE	2018
Feature-wise transformations	2018
RAVEN: A Dataset for Relational and Analogical Visual rEasoNing	2019
A Dataset and Architecture for Visual Reasoning with a Working Memory	2018
Cycle-Consistency for Robust Visual Question Answering	2019
On Self Modulation for Generative Adversarial Networks	2019
Interactive Sketch & Fill: Multiclass Sketch-to-Image Translation	2019
TapNet: Neural Network Augmented with Task-Adaptive Projection for Few-Shot Learning	2019
Predicting Taxi Demand Based on 3D Convolutional Neural Network and Multi-task Learning	2019

Table S10. Long short-term memory: Clustering effect example. First line is the parent paper and the rest children.

agriculture and 'Visual Reasoning with a General Conditioning Layer' leads a handful of articles specialising in visual reasoning (Table S10). We also identify some bundles dealing with energy forecast and financial trading. All these observations confirm the effectiveness of our skeleton tree extraction algorithm. Moreover, these aforementioned bundles were born no earlier than 2018, thus they are also good illustrations of some latest research hotspots in the topic.

When comparing T^t's evolution with the keyword frequency of the pioneering work queried from Baidu Xueshu, we find the evolution of topic knowledge temperature well corresponds with the keyword occurence trend until 2016. Since 1998, keyword 'short-term memory' has experienced 2 hot periods: from 1999 to 2003 and from 2010 to 2014 (Fig. S42). Indeed, we observe 2 small peaks in T^t's dynamics until 2016, the first one taking place around 2001 and the second one around 2015 (Fig. S39). Globally speaking, 'short-term memory' has been gaining popularity since 2006, a trend which T^t also follows.

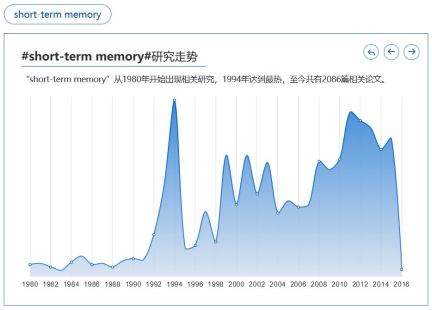

Fig S42. Long short-term memory: Popularity trend of pioneering work's keywords provided by baidu research engine.

S2.3.2 Particle swarm optimization

The topic gained popularity and expanded its impact steadily from its birth until around 2004 largely under the joint efforts of the pioneering work and several well-developed child papers published before 2000, namely 'A modified particle swarm optimizer','Empirical study of particle swarm optimization', and 'Parameter Selection in Particle Swarm Optimization'. It is also these prominent child papers within the topic that lay the foundation of the skeleton tree (Fig. S44). Another 2 influential younger child papers, 'Comparing inertia weights and constriction factors in particle swarm optimization' published in 2000 and 'The particle swarm - explosion, stability, and convergence in a multidimensional complex space' published in 2002, opened up a smaller sub-topic, which is visualized as the smaller major arm that extend from the central cluster. Their arrival ensured topic's thriving in its first 10 years, which is reflected by a rising T^t_{growth} and a relatively high $T^t_{structure}$ during that period. In comparison, nothing remarkable happened in the following 5 years. Papers published during this period simply extended the established sub-topics. As a result, T^t and its components stagnated (Fig. S43). Next, the machine learning wave revitalized the topic. Starting from somewhere between 2010 and 2013, novel research focuses have been derived from the older sub-topics and some of them already had certain development (Fig. S45 (e,f)). This phenomenon is illustrated by the increasingly rich end structure of skeleton tree. In addition, annual publication number reached record high for the year 2014. The accelerated topic expansion brought about a significant spike in useful information inflow (Fig. 5(l)), which resulted in a non-trivial increase in T^t from 2013 to 2016. As the tendency is cooling down now, so is the topic. Overall, this is a topic waken up by the AI booming.

There is a small cold cluster detached from the topic majority (Fig.S45 in the top-right of (f)). This cluster is led by popular child paper 'A new optimizer using particle swarm theory' published in the same year as the pioneering work. Thus the two papers probably have different focus even though they bear resemblance in their ideas. Their divergences cause their separation in the skeleton tree and their distinct knowledge temperatures. The separated skeleton tree also accords with topic's galaxy map representation where it seems to be split into 2 parties (Fig. S44).

Now we closely examine the internal heat distribution together with its latest skeleton tree (Fig. S44 bottom left). After 25 years of development, the heat has already fulled diffused to the entire topic, as most popular child papers that founded recent research focuses have a knowledge temperature above average. They are the topic's heat sources. It is clear that paper knowledge temperature decreases globally as the articles are located farther away from multiple research centers. This fits the general rule "the older the hotter" (Fig. 6(l)). Note that the colder average knowledge

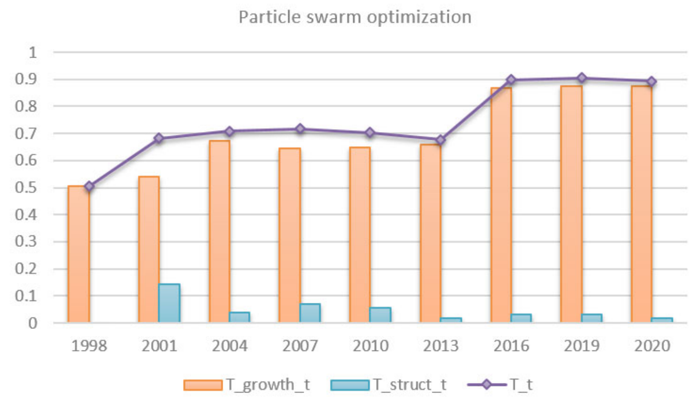

year	$\lvert V^t \rvert$	$\lvert E^t \rvert$	n_t	V_t	$UsefulInfo^t$	T^t_{growth}	T^t_{struct}	T^t
1998	16	32	10.9	16	5.1	0.504		0.504
2001	69	215	43.86	69	25.14	0.54	0.142	0.683
2004	494	2272	252.724	494	241.276	0.671	0.037	0.708
2007	2818	13285	1500.051	2818	1317.949	0.645	0.071	0.717
2010	9186	44357	4877.391	9186	4308.609	0.647	0.056	0.703
2013	17705	90172	9243.861	17705	8461.139	0.658	0.019	0.676
2016	26159	143862	10349.479	26159	15809.521	0.868	0.0305	0.899
2019	31436	180700	12357.104	31436	19078.897	0.874	0.032	0.906
2020	31800	183342	12502.285	31800	19297.715	0.874	0.019	0.893

Fig S43. Particle swarm optim: topic statistics and knowledge temperature evolution

temperatures among the oldest articles is caused by the "cold" popular child paper mentioned in the previous paragraph and the relatively independent research branch it leads. This child paper is also responsible for the drastic average temperature plunge in most-cited papers (Fig. S63(l)). Besides, the blue nodes that surround the pioneering work and popular child papers in non-trivial clusters are papers with few or without any in-topic citations. However, the general rule is violated even if we do not consider this "cold" research branch. For example, 'Path planning for mobile robot using the particle swarm optimization with mutation operator' is slightly colder than its child paper 'Classic and Heuristic Approaches in Robot Motion Planning A Chronological Review'. The former is slightly colder than the latter (Fig. S44 top zoom in bottom right). Their temperature difference is mainly due to their different research focus, which is reflected by their distinct citations. Similarly, paper 'Using neighbourhoods with the guaranteed convergence PSO' is also colder than its child paper 'A guaranteed convergence dynamic double particle swarm optimizer'. The former is also a bit colder than the latter (Fig. S44 bottom zoom in bottom right). These counter examples illustrate that the general rule "the older the hotter" is not robust.

We observe in addition certain clustering effect in the skeleton tree. For example, almost all child papers of 'A self-generating fuzzy system with ant and particle swarm cooperative optimization' deal with fuzzy rule (Table S11). This confirms the effectiveness of our skeleton tree extraction algorithm.

When comparing T^t's evolution and useful information accumulation process with the keyword frequency of the pioneering work queried from Baidu Xueshu, we find the evolution of both quantities is similar to the keyword occurence trend until 2015. Since 1996, keyword 'artificial intelligence' has become most "hot" during 2008 and 2011 (Fig. S46). Another keyword, 'neural nets', was most trendy between 2009 and 2011 and this field has been regaining attention since 2013. As for the other 2 keywords, 'particle

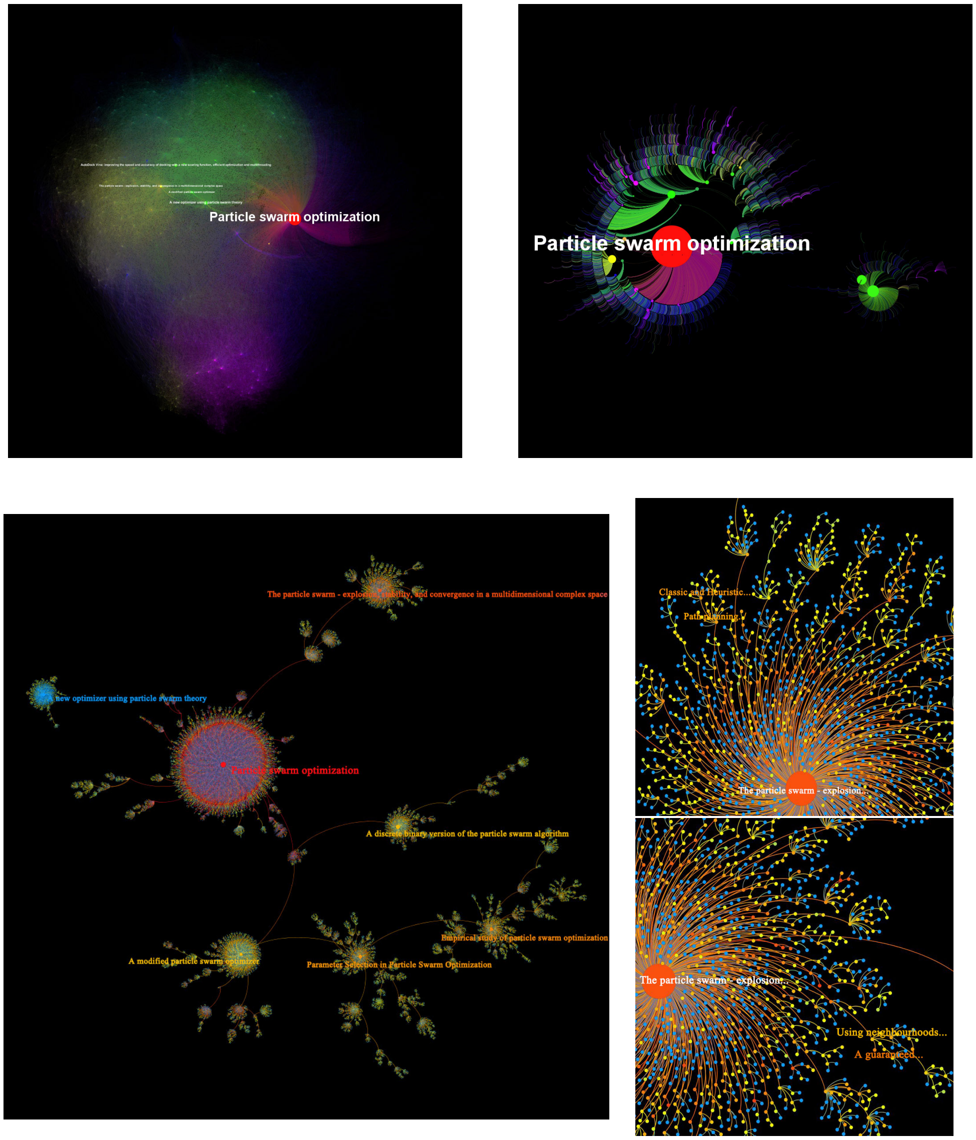

Fig S44. Particle swarm optim: Galaxy map and current skeleton tree. Top left: current galaxy map. Papers with the most total citations are labelled by their titles. Top right: current skeleton tree in circular layout, node color is the same as in galaxy map. Bottom left: current skeleton tree in ForceAtlas layout, node color is determined by paper knowledge temperature, with blue the coldest and red the hottest. Papers with more than 1700 in-topic citations are labelled by title. Except the pioneering work, labelled nodes' size is amplified by 5 times. Bottom right: 2 regional zooms of current skeleton tree in ForceAtlas layout.

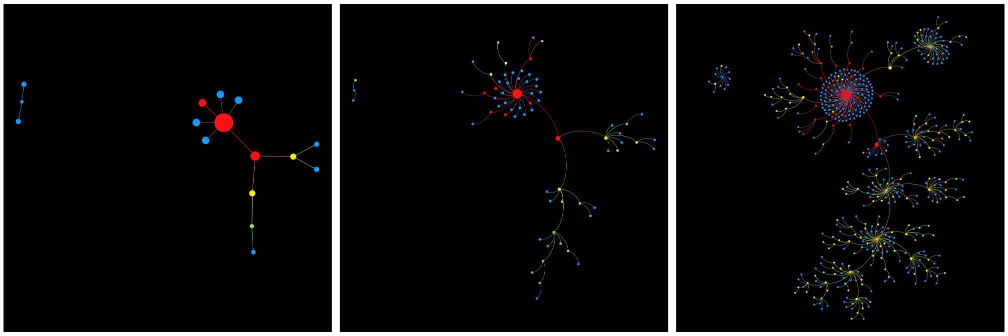

(a) Skeleton tree until 1998 **(b)** Skeleton tree until 2001 **(c)** Skeleton tree until 2004

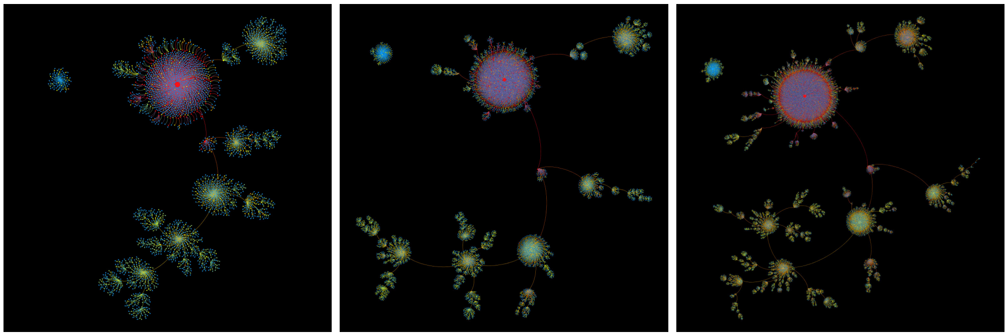

(d) Skeleton tree until 2007 **(e)** Skeleton tree until 2010 **(f)** Skeleton tree until 2016

Fig S45. Particle swarm optim: Skeleton tree evolution

title	year
A self-generating fuzzy system with ant and particle swarm cooperative optimization	2009
ANFIS modelling of a twin rotor system using particle swarm optimisation and RLS	2010
Improving fuzzy knowledge integration with particle swarmoptimization	2010
Designing Fuzzy-Rule-Based Systems Using Continuous Ant-Colony Optimization	2010
Fuzzy Neural Networks Learning by Variable-Dimensional Quantum-behaved Particle Swarm Optimization Algorithm	2013
Modeling and OnLine Control of Nonlinear Systems using Neuro- Fuzzy Learning tuned by Metaheuristic Algorithms	2014

Table S11. Particle swarm optim: Clustering effect example. First line is the parent paper and the rest children.

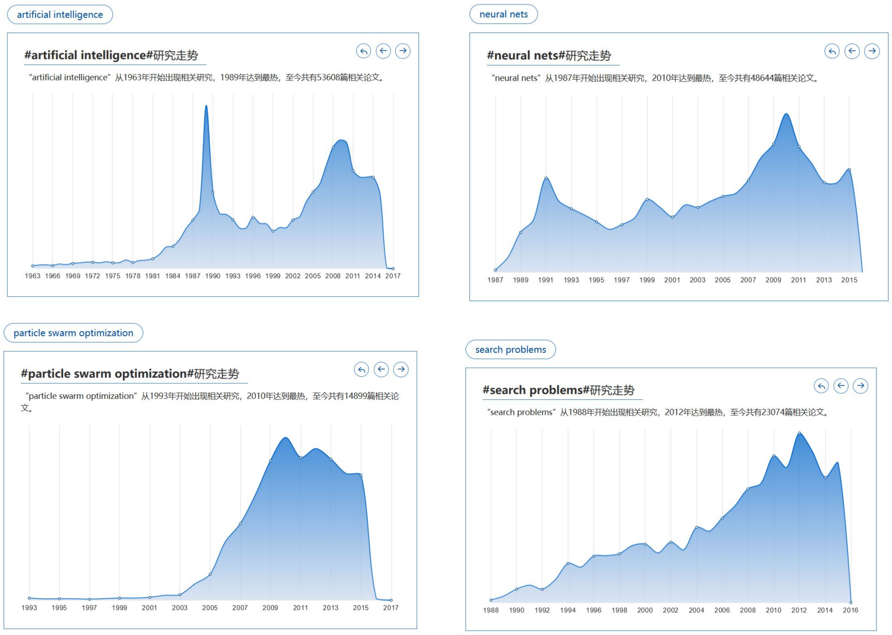

Fig S46. Particle swarm optim: Popularity trend of pioneering work's keywords provided by baidu research engine.

swarm optimization' and 'search problems', they became popular from around 2010 and kept a relatively stable popularity level until 2015. We indeed observe an accelerated useful information storage from 2007 to 2016 (Fig. 5(l)), which corresponds well with the golden ages of the 3 keywords. The sudden increase in T^t during 2013 and 2016 not only is consistent with the heat trend of keywords 'particle swarm optimization' and 'search problems', but moreover it is the fruit of prior useful information accumulation, which is demonstrated by a surge in new publications (Fig. S43).

S2.4 Rise-fall-cycle topics

S2.4.1 On random graphs, I

As is shown by T^t and T^t_{growth}, the impact and popularity evolution of this topic is a bit complicated (Fig. S47). The publication of popular child paper 'On the evolution of random graphs' (OERG) in 1984 brought the first boom in the 1980s. This article combined its ancestors' ideas and successfully fused the previously separated parts in skeleton tree due to an atypical citation from an older article 'On the existence of a factor of degree one of a connected random graph' (Fig. S48(b,c)). This merge is the first significant evolution in knowledge structure and thus led to a spike in $T^t_{structure}$. Afterwards, the topic went relatively silent in the 1990s before a group of popular child papers came during 2001 and 2003. Among these articles, 'Random graphs with arbitrary degree distributions and their applications' published in 2001 non-trivially furthered the study of OERG and introduced a new research focus into the topic, as is illustrated by the emergence of a third cluster in the skeleton tree (Fig. S48(f,g)). Its followers and popular child papers, 'Evolution of networks' published in 2002 and 'The Structure and Function of Complex Networks' published in 2003 extended its idea and created several new research sub-fields. That is why we observe some splits derived from the young cluster (Fig. S48(g)). They successfully attracted a lot of attention in a short time and the topic has witnessed an accelerated expansion since around 2000. Since

then, the topic has quickened its pace in useful information collection (Fig. 5(m)). Meanwhile, these popular child papers also contributed to a non-trivial extension to topic knowledge structure. Consequently, this topic experienced another booming around 2010. Later, the topic kept its activity thanks to several young promising papers including ‘Measurement and analysis of online social networks’ published in 2007, ‘Community detection in graphs’ published in 2010 and ‘Catastrophic cascade of failures in interdependent networks’ published in 2010. Although they opened up several new research orientations, there have not been a substantial subsequent development and the branches leading by them remain small in comparison to the principal clusters (Fig. S48(h,f)). Consequently, they have mostly helped maintain the topic’s visibility and its stable impact.

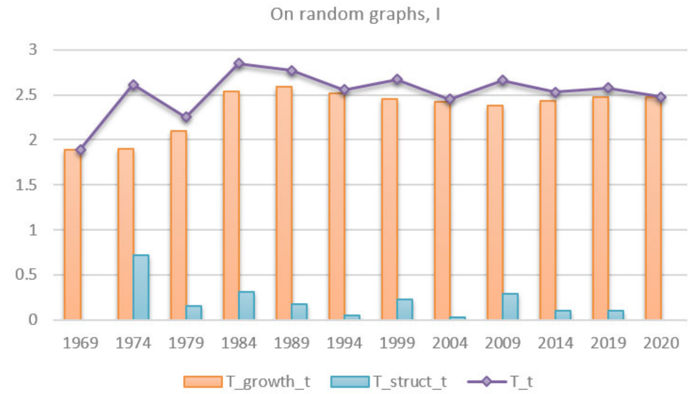

year	$\lvert V^t \rvert$	$\lvert E^t \rvert$	n_t	V_t	$UsefulInfo^t$	T^t_{growth}	T^t_{struct}	T^t
1969	5	6	4	5	1	1.892		1.892
1974	14	20	11.167	14	2.833	1.898	0.714	2.612
1979	30	57	21.606	30	8.394	2.102	0.151	2.253
1984	59	157	35.23	59	23.77	2.535	0.314	2.849
1989	87	240	50.815	87	36.185	2.592	0.176	2.768
1994	111	288	66.871	111	44.129	2.513	0.045	2.558
1999	135	334	83.416	135	51.584	2.45	0.222	2.672
2004	311	832	194.093	311	116.907	2.426	0.024	2.45
2009	1346	4172	856.782	1346	489.218	2.378	0.284	2.663
2014	3312	10406	2063.311	3312	1248.689	2.43	0.099	2.529
2019	5387	17095	3295.006	5387	2091.994	2.475	0.102	2.577
2020	5389	17098	3294.798	5389	2094.202	2.476	0	2.476

Fig S47. On random graphs: topic statistics and knowledge temperature evolution

Now we closely examine the internal heat distribution together with its latest skeleton tree (Fig. S49 bottom). The topic has a long development history. In each period, new research focuses emerged (Fig. S48 every line shows a period). Today, we see 3 major research focuses and their founders are all the heat sources. As the articles are located farther away from the pioneering paper or the sub-topic centers, their paper knowledge temperature decreases globally. The blue nodes that surround the pioneering work and popular child papers in main clusters are papers with few or without any in-topic citations. Generally speaking, older papers are hotter than the younger (Fig. 6(m)). In comparison with other scientific topics, knowledge temperature fluctuates more among the “middle-aged” papers. This phenomenon is in line with the up and downs the topic experienced during their publication period. Besides, we also observe a general rule “the more influential the hotter” in the topic (Fig. S63(m)) as the

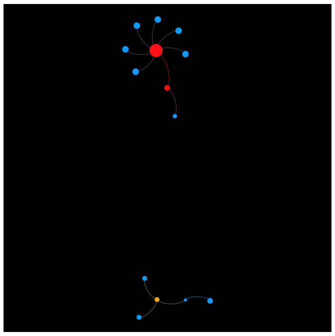

(a) Skeleton tree until 1974

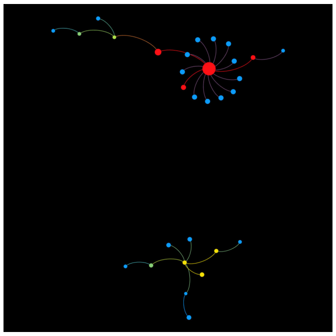

(b) Skeleton tree until 1979

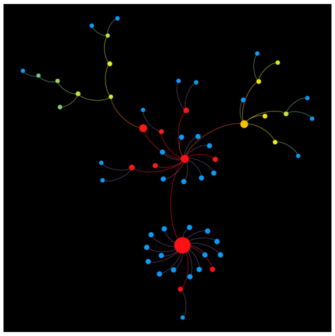

(c) Skeleton tree until 1984

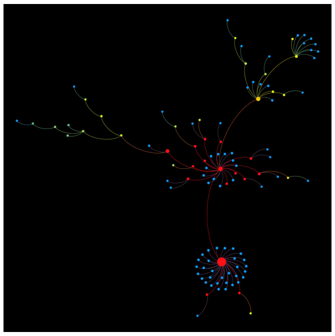

(d) Skeleton tree until 1994

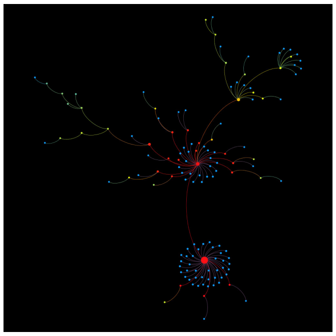

(e) Skeleton tree until 1999

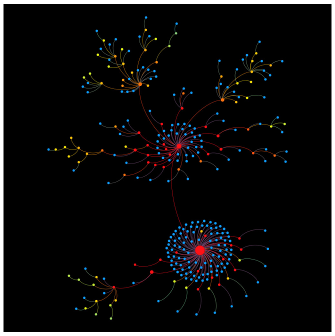

(f) Skeleton tree until 2004

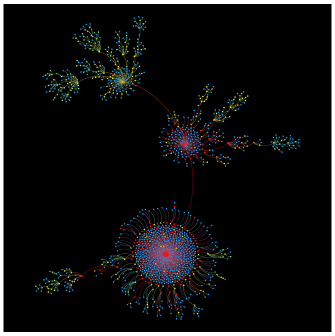

(g) Skeleton tree until 2009

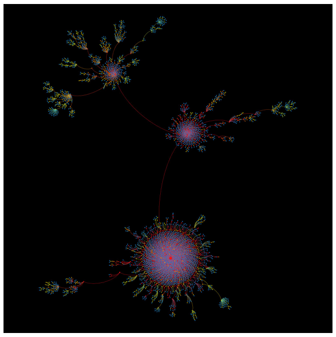

(h) Skeleton tree until 2014

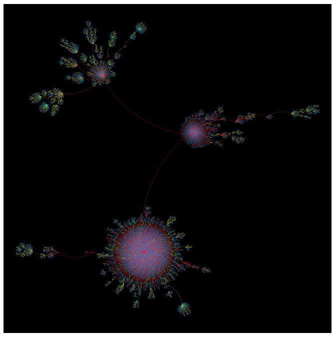

(i) Skeleton tree until 2019

Fig S48. On random graphs: Skeleton tree evolution

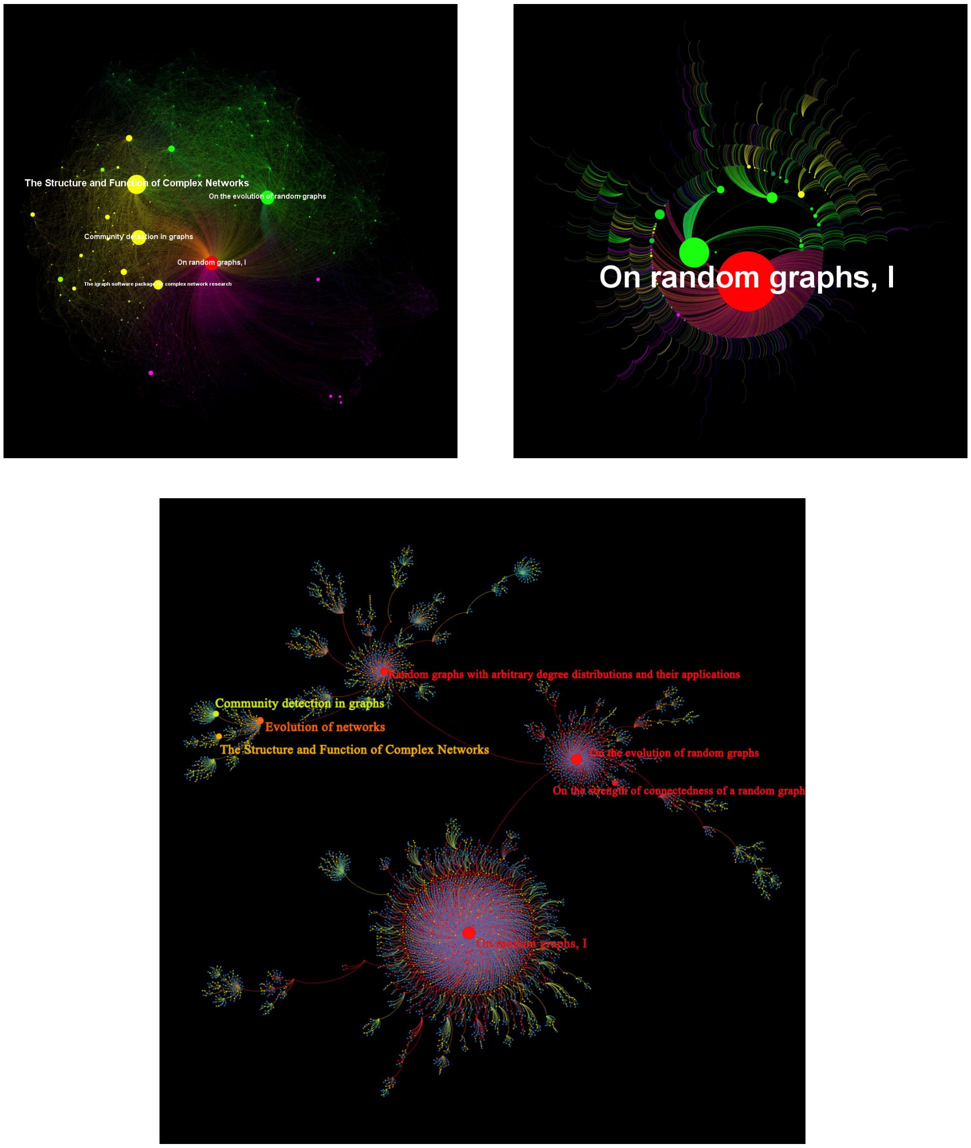

Fig S49. On random graphs: Galaxy map and current skeleton tree. Top left: current galaxy map. Papers with the most total citations are labelled by their titles. Top right: current skeleton tree in circular layout, node color is the same as in galaxy map. Bottom: current skeleton tree in ForceAtlas layout, node color is determined by paper knowledge temperature, with blue the coldest and red the hottest. Papers with more than 200 in-topic citations are labelled by title. Except the pioneering work, labelled nodes' size is amplified by 5 times.

title	year
False Beliefs in Unreliable Knowledge Networks	2017
Communication Policies in Knowledge Networks	2018
Experts in Knowledge Networks: Central Positioning and Intelligent Selections	2018
How to facilitate knowledge diffusion in complex networks: The roles of network structure, knowledge role distribution and selection rule	2019

Table S12. On random graphs: Clustering effect example. First line is the parent paper and the rest children.

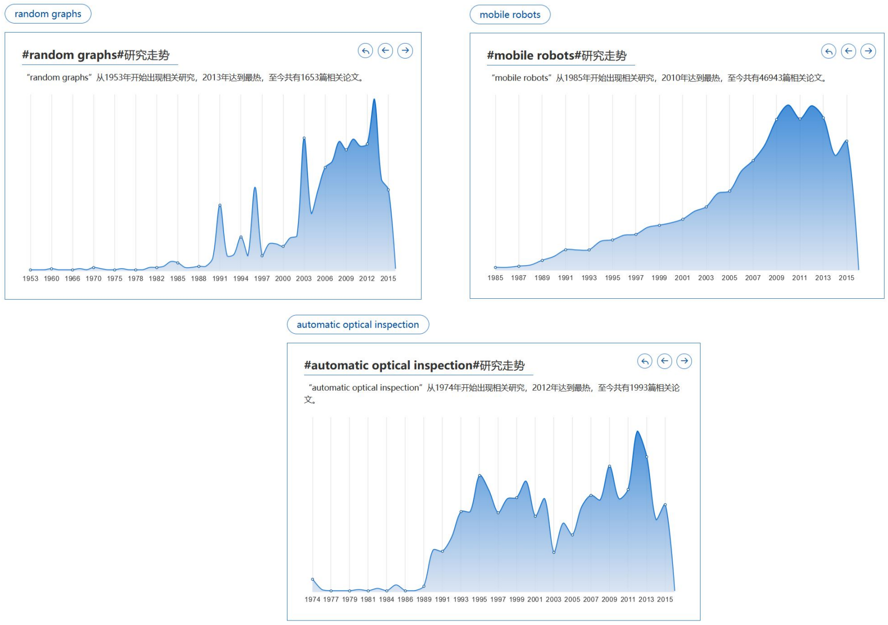

Fig S50. On random graphs: Popularity trend of pioneering work's keywords provided by baidu research engine.

most-cited child papers are among the hottest articles. However, this rule is only robust for the most eminent child papers.

We observe in addition certain clustering effect in the skeleton tree (Table S12). For example, all child papers of 'False Beliefs in Unreliable Knowledge Networks' probe into knowledge network. This confirms the effectiveness of our skeleton tree extraction algorithm. Moreover, the small group was born in 2017, suggesting that their research focus, knowledge network, may be one of the latest hotspots within the topic.

When comparing T^t's evolution with the keyword frequency of the pioneering work queried from Baidu Xueshu, we find T^t's dynamic is similar to the keyword occurence trend until 2015. Since 1960, keywords 'random graphs' and 'automatic optical inspection' have enjoyed periodic popoularity and their patterns resemble that of T^t's dynamics (Fig. S50). The third keyword, 'mobile robot' is an important recent application field of the pioneering work's theory. It has had a increasing popularity until 2013. The emergence of this field is part of the reason why the topic keeps its vigor after over 50 years of development (Fig. S47).

S2.4.2 Collective dynamics of 'small-world' networks

As is shown by T^t, although the topic is heating up thanks to a robust knowledge accumulation, it has experienced multiple ups and downs during the past 20 years due to short-term popularity fluctuations (Fig. S51). This topic has welcome 2 waves of popular child papers, the first coming between its birth and 2003 and the second batch being published around 2009 and 2010. The oldest popular articles, namely 'Emergence of Scaling in Random Networks' (ESRN) published in 1999 in Science, 'Exploring complex networks' published in 2001 in Nature and 'Community structure in social and biological networks' published in 2002 shaped the fundamentals of topic knowledge structure together with the pioneering work by 2007 (Fig. S52(c), S53). Their substantial contribution to the knowledge quantity and diversity led to a fast rise in both T^t_{growth} and $T^t_{structure}$. As a result, the topic reached the first peak around 2007. For the following years, the short-term exposure increase brought by these eminent child papers gradually wore off and few child papers emerged as rising stars. The topic development during this period was primarily a fortification of its existing knowledge architecture. That is why the topic slightly cooled down during 2007 and 2010 despite a robust topic expansion and an on-going useful information accumulation (Fig. 5(n)). It was also during this down period when the younger popular child papers were published. Some of them, including 'Complex brain networks: graph theoretical analysis of structural and functional systems' published in 2009 and 'Complex network measures of brain connectivity: Uses and interpretations' published in 2010, introduced new research sub-fields closely related to the idea of the pioneering work. They both formed a non-trivial branch extending directly out of the central cluster (Fig. S52(e,f)). Others continued to enrich the existing research fields created by former eminent child papers. For example, 'Emergence of Scaling in Random Networks' demonstrated an exceptional capability to attract substantially more subsequent works even after 10 years of its publication thanks to the explosive growth of social networks. The new knowledge extension and the lasting refinement of the entire knowledge framework are portrayed by a flourishing topic skeleton tree with multidimensional development and a steadily rising T^t until 2016, a year when the topic hit the second peak. While the first golden age is essentially owing to a rapid internal growth, the second streak is largely propelled by favorable social trends, especially the prevalence of online social network and the popularization of brain or neuroscience. Recently, the short-term focus benefit has been dying out and no remarkable progress have been matured enough to cause a stir. Thus the topic is now seeing a small slip.

Now we closely examine the internal heat distribution together with its latest skeleton tree (Fig. S53 bottom left). All popular child papers have a knowledge temperature above average. This shows that the heat diffusion within the topic is completed after over 20 years of development. Most research focuses derived from the original ideas of the pioneering work have had some substantial development. The ensemble makes up the majority of heat sources within the topic. Besides, we also spot few atypical heat sources. They are articles that connect non-trivial research directions in the skeleton tree. For example, paper 'Combatting maelstroms in networks of communicating agents' published in 1999 connects the entire left research branch and the central cluster led by the pioneering work. It does not have any direct followers on skeleton tree, but it is the hottest node and its big structure entropy suggests that it is important to the entire knowledge framework. Its value lies exclusively in the enlightenment. As the articles are located farther away from these heat sources, their node knowledge temperature decreases. This accords with the general rule "the older the hotter" (Fig. 6(n)). Note that the average temperature for the oldest papers is not the highest. This is due to the presence of 3 "cold" articles published in the same year as the pioneering work. They either hardly inspired any subsequent works or failed to

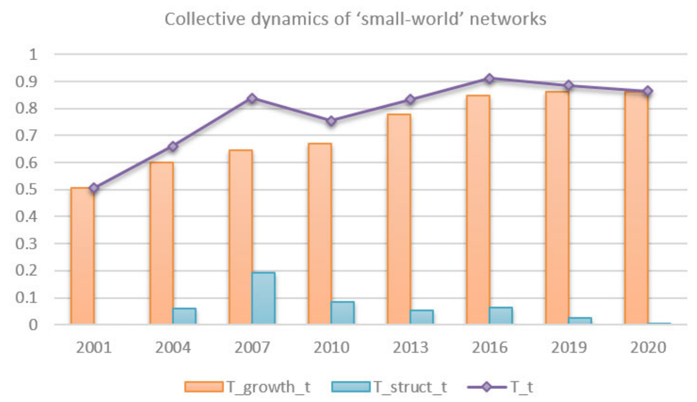

year	$\|V^t\|$	$\|E^t\|$	n_t	V_t	$UsefulInfo^t$	T^t_{growth}	T^t_{struct}	T^t
2001	246	754	169.095	246	76.905	0.505		0.505
2004	1404	7192	812.506	1404	591.494	0.6	0.059	0.66
2007	4209	26584	2260.505	4209	1948.495	0.647	0.192	0.839
2010	8517	58620	4415.143	8517	4101.857	0.67	0.085	0.755
2013	13998	104667	6245.327	13998	7752.673	0.779	0.054	0.833
2016	20221	158518	8280.879	20221	11940.121	0.848	0.062	0.91
2019	25313	204644	10197.242	25313	15115.759	0.863	0.023	0.886
2020	25548	206643	10288.162	25548	15259.839	0.863	0.001	0.863

Fig S51. small-world: topic statistics and knowledge temperature evolution

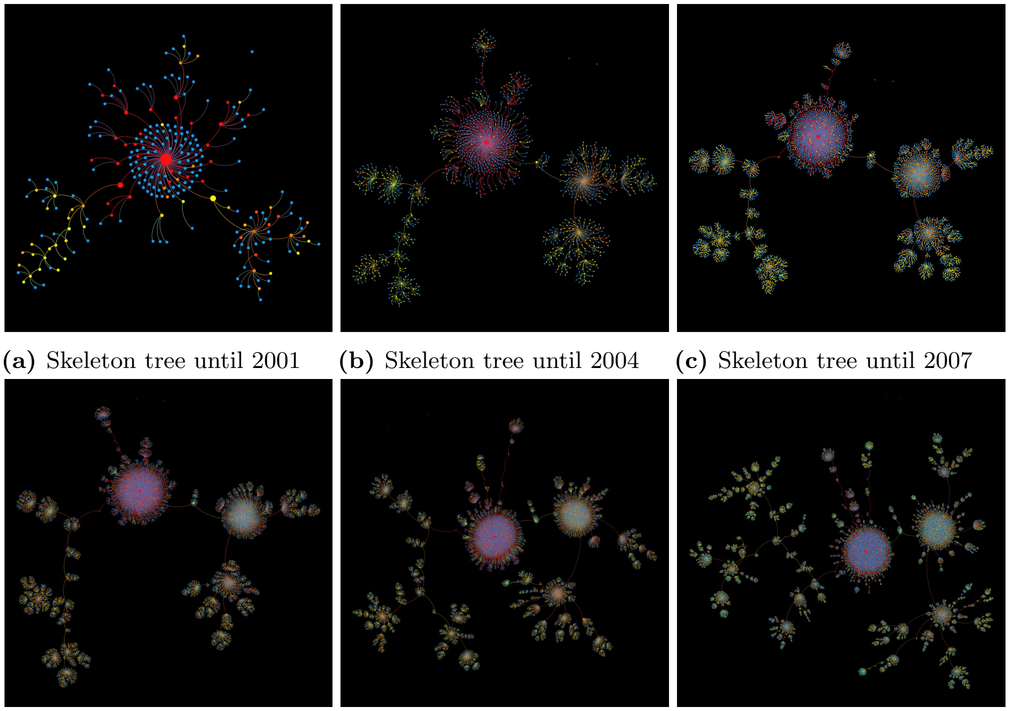

(a) Skeleton tree until 2001 **(b)** Skeleton tree until 2004 **(c)** Skeleton tree until 2007

(d) Skeleton tree until 2010 **(e)** Skeleton tree until 2013 **(f)** Skeleton tree until 2016

Fig S52. small-world: Skeleton tree evolution

title	year
Robustness of Synchrony in Complex Networks and Generalized Kirchhoff Indices	2018
Impact of network topology on the stability of DC microgrids	2019
The key player problem in complex oscillator networks and electric power grids: Resistance centralities identify local vulnerabilities	2019
Quantifying transient spreading dynamics on networks	2019
Global robustness versus local vulnerabilities in complex synchronous networks	2019

title	year
Multiplex lexical networks reveal patterns in early word acquisition in children	2017
Multiplex model of mental lexicon reveals explosive learning in humans	2018
How children develop their ability to combine words: a network-based approach	2019
Multiplex model of mental lexicon reveals explosive learning in humans	2018
Applying network theory to fables: complexity in Slovene belles-lettres for different age groups	2019
Knowledge gaps in the early growth of semantic feature networks	2018
The orthographic similarity structure of English words: Insights from network science	2018
Node Ordering for Rescalable Network Summarization (or, the Apparent Magic of Word Frequency and Age of Acquisition in the Lexicon)	2018
spreadr: An R package to simulate spreading activation in a network	2019

Table S13. small-world: Clustering effect example. First line is the parent paper and the rest children.

attract the attention of recent researches. Besides, the blue nodes that surround the pioneering work and the most popular child papers in principal clusters in the current skeleton tree are papers with little or no in-topic development. However, the general rule is violated even if we let alone the oldest articles. For example, paper ESRN is slightly colder than its child papers, 'The large-scale organization of metabolic networks.' published in 2000 in Nature and 'Classes of small-world networks' published in 2000. Both are coloured red while ESRN is coloured orange-red. The temperature difference is mainly due to their different research focus, as is reflected by their distinct citations. The counter example also illustrates that the general rule "the more influential the hotter" is weak (Fig. S63(n)). Last but not the least, we find that most articles published in top journals such as Science and Nature have high knowledge temperatures and numerous citations. This accords with the prior study which points out the boosting effect of renowned journals on articles[30].

We observe in addition certain clustering effect in the skeleton tree (Table S13). This confirms the effectiveness of our skeleton tree extraction algorithm. Moreover, these newly-formed small groups are very young, suggesting that their research focus may be among the latest hotspots within the topic.

When comparing T^t's evolution with the keyword frequency of the pioneering work queried from Baidu Xueshu, we find T^t's dynamics is somewhat similar to the keyword occurence trend until 2015. Since 1999, both keywords 'nerve nets' and 'caenorhabditis elegans' continuously gained popularity until around 2012 (Fig. S54). Correspondingly, T^t globally demonstrated an up-trend during this period. Furthermore, T^t_{growth} also climbed up faster during the same period than afterwards (Fig. S51).

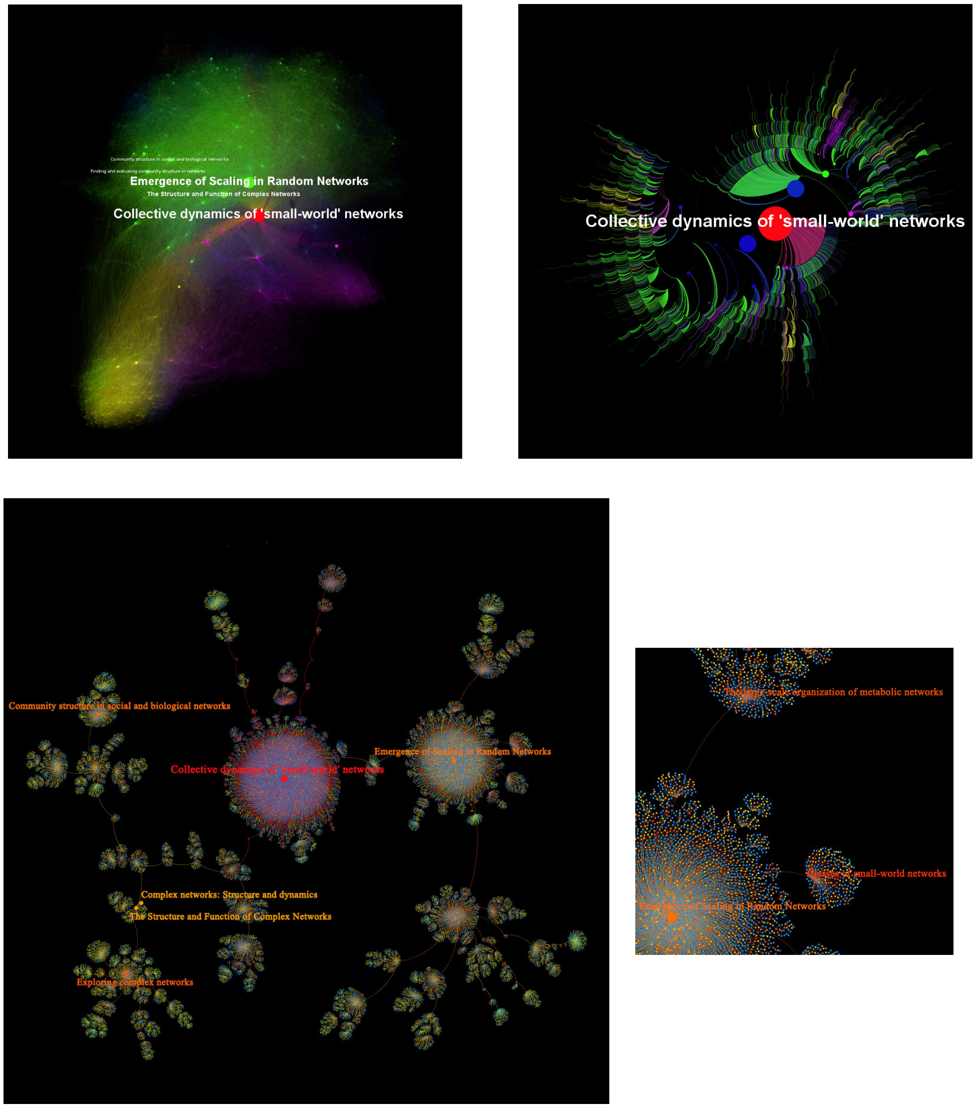

Fig S53. small-world: Galaxy map, current skeleton tree and its regional zoom. Top left: current galaxy map. Papers with the most total citations are labelled by their titles. Top right: current skeleton tree in circular layout, node color is the same as in galaxy map. Bottom left: current skeleton tree in ForceAtlas layout, node color is determined by paper knowledge temperature, with blue the coldest and red the hottest. Papers with more than 2000 in-topic citations are labelled by title. Except the pioneering work, labelled nodes' size is amplified by 6 times. Bottom right: regional zoom of current skeleton tree in ForceAtlas layout.

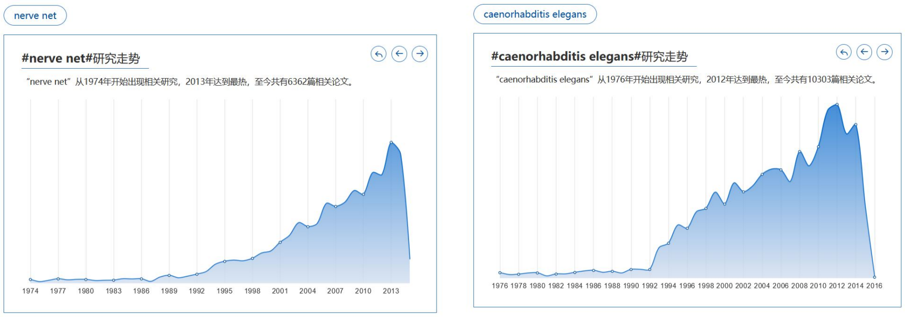

Fig S54. small-world: Popularity trend of pioneering work's keywords provided by baidu research engine.

S2.4.3 Latent dirichlet allocation

As is shown by T^t, the impact and popularity evolution of the topic fluctuates. After reaching the first peak around 2010, this field cooled down for a while before it became trendy again around 2019 (Fig. S55). In the long run, the topic has an increasing impact. The rise-and-fall pattern is largely due to the short-term popularity fluctuations, as is demonstrated by the variation of $T^t_{structure}$. In its first 10 years, the topic developed 3 principal research sub-fields, as is illustrated by the skeleton tree (Fig. S56 (a,b,c)). The advancement is largely owing to the the arrival of several influential child papers within the topic around 2005 and 2006: 'A Bayesian hierarchical model for learning natural scene categories', 'Hierarchical Dirichlet Processes' and 'Dynamic topic models' (Fig. S57). They increased the exposure of this topic, facilitated a rapid knowledge accumulation and enriched greatly the knowledge structure. Consequently, the topic had its first golden period. Afterwards, the sweeping trend of machine learning helped the topic gain more attention and fame. A new wave of popular papers joining between 2009 and 2012 gradually manifested their attractiveness, namely 'Labeled LDA: A supervised topic model for credit attribution in multi-labeled corpora','Reading Tea Leaves: How Humans Interpret Topic Models' and 'Probabilistic topic models'. They extended the former research focuses and provided inspiration for novel, promising ideas. This is captured by the increasingly complex major branches in skeleton tree (Fig. S56 (e,f)) and a continuously accelerated accumulation in useful information (Fig. 5(o)). In particular, this wave brought a large amount of attention immediately to the topic and created a second glory.

Now we closely examine the internal heat distribution together with its latest skeleton tree (Fig. S57 bottom left). After over 20 years of development, the original and recent research ideas have all had a rich development. The heat is therefore diffused to every corner of the skeleton tree with the help of popular child papers. Apart from multiple heat sources in the core of research branches, we also identify some hottest articles between principal clusters. For example, paper 'Variational extensions to EM and multinomial PCA' published in 2002 connects the entire right branch and the central cluster. It does not have many direct followers within the topic, but it is one of the hottest papers within the topic and it has a big structure entropy due to its knowledge bridging value. As the articles are located farther away from these hot papers, their node knowledge temperature decreases. This accords with the general rule "the older the hotter" (Fig. 6(o)). The blue nodes that surround the pioneering work and popular child papers in central parts are papers with few or without any in-topic followers. However, there are exceptions. Paper 'You Are What You Tweet: Analyzing Twitter for Public Health' (YWTPH) published in 1998 is colder than its child papers, 'Using Twitter for breast cancer prevention: an analysis of breast cancer awareness

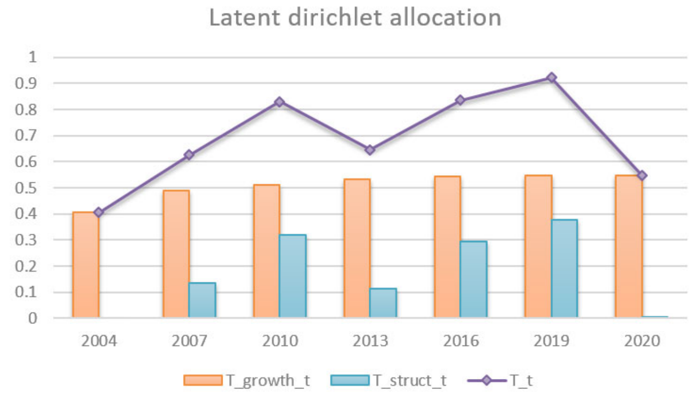

year	$\|V^t\|$	$\|E^t\|$	n_t	V_t	$UsefulInfo^t$	T^t_{growth}	T^t_{struct}	T^t
2004	95	190	63.402	95	31.598	0.406		0.406
2007	554	1931	306.602	554	247.398	0.489	0.136	0.626
2010	2287	10388	1211.237	2287	1075.763	0.511	0.319	0.83
2013	6302	33738	3193.479	6302	3108.521	0.534	0.112	0.646
2016	12945	75942	6459.428	12945	6485.572	0.542	0.293	0.835
2019	18583	113483	9213.456	18583	9369.544	0.546	0.377	0.923
2020	18813	114970	9330.17	18813	9482.83	0.546	0.002	0.548

Fig S55. LDA: topic statistics and knowledge temperature evolution

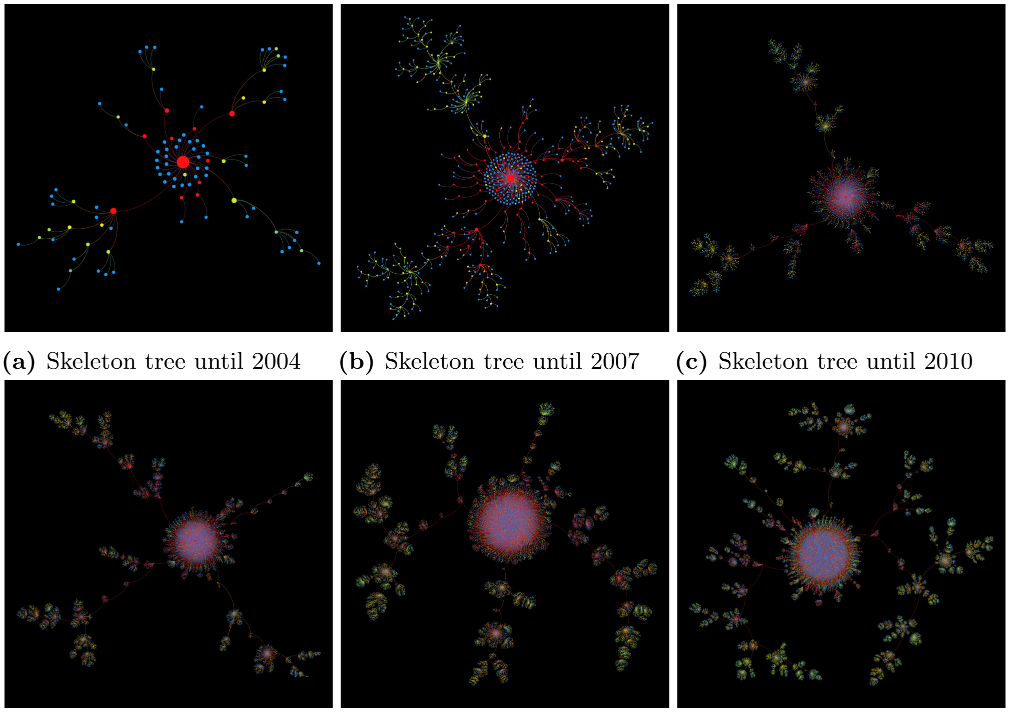

(a) Skeleton tree until 2004 **(b)** Skeleton tree until 2007 **(c)** Skeleton tree until 2010

(d) Skeleton tree until 2013 **(e)** Skeleton tree until 2016 **(f)** Skeleton tree until 2019

Fig S56. LDA: Skeleton tree evolution

title	year
The spread of true and false news online	2018
Assessing the Readiness of Academia in the Topic of False and Unverified Information	2019
Ginger Cannot Cure Cancer: Battling Fake Health News with a Comprehensive Data Repository	2020
Early Public Responses to the Zika-Virus on YouTube: Prevalence of and Differences Between Conspiracy Theory and Informational Videos	2018
An opinion based cross-regional meteorological event detection model	2019
Investigating Italian disinformation spreading on Twitter in the context of 2019 European elections	2020

title	year
Automated Text Analysis for Consumer Research	2018
Automated Text Analysis	2019
Mining Product Relationships for Recommendation Based on Cloud Service Data	2018
Text mining analysis roadmap (TMAR) for service research	2020
Uniting the Tribes: Using Text for Marketing Insight:	2019

Table S14. Clustering effect example. First line is the parent paper and the rest children.

month' published in 2013 and 'Global Disease Monitoring and Forecasting with Wikipedia' published in 2014. The latter two are coloured in orange-red while YWTPH is coloured in yellow-green. Their temperature difference lies primarily in their different research focus reflected by their distinct in-topic citations. This counter example also suggests that another general rule "the more influential the hotter" is not robust (Fig. S63(o)).

We observe in addition certain clustering effect in the skeleton tree (Table S14). This confirms the effectiveness of our skeleton tree extraction algorithm. Moreover, these mini-groups are very young, suggesting that their research focus may be among the latest hotspots within the topic.

When comparing T^t's evolution with the keyword frequency of the pioneering work queried from Baidu Xueshu, we find T^t's dynamics is somewhat similar to the keyword occurence trend until 2015. Since 2004, the only keyword of the topic, which is exactly the title of the pioneering work, 'latent dirichlet allocation', has had a rising popularity in general (Fig. S58). This is in line with an accelerated accumulation in useful information taken place within the topic (Fig. 5(o)).

S2.4.4 A FUNDAMENTAL RELATION BETWEEN SUPERMASSIVE BLACK HOLES AND THEIR HOST GALAXIES

The knowledge temperature evolution of this topic is quite unique. Not only T^t manifests multiple local peaks every 6 years, but more importantly it is $T^t_{structure}$ that dominates the ups and downs of T^t (Fig. S59). As for T^t_{growth}, its increase in the early days is due to the continual arrival of popular child papers within the topic until 2006. They brought a steady inflow of new knowledge that enriched the topic content. Almost all the popular papers published after 2008 have not so far achieved a comparable development.

The skeleton tree of this topic is also very special in that there are much fewer child papers surrounding the pioneering work, the biggest red node situated in bottom-right, than its prominent descendants, 'A Relationship between nuclear black hole mass and

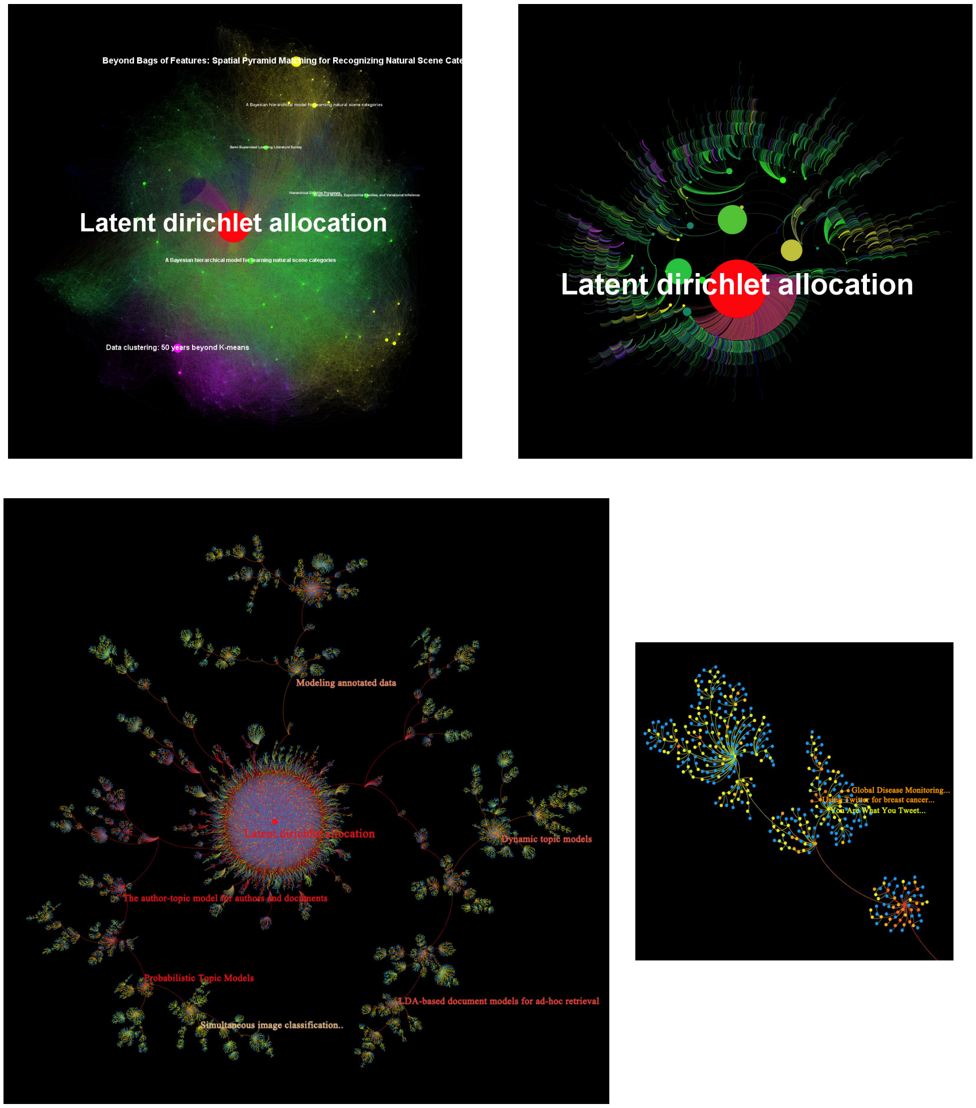

Fig S57. LDA: Galaxy map, current skeleton tree and its regional zoom. Top left: current galaxy map. Papers with the most total citations are labelled by their titles. Top right: current skeleton tree in circular layout, node color is the same as in galaxy map. Bottom left: current skeleton tree in ForceAtlas layout, node color is determined by paper knowledge temperature, with blue the coldest and red the hottest. Papers with more than 700 in-topic citations are labelled by title. Except the pioneering work, labelled nodes' size is amplified by 5 times. Bottom right: regional zoom of current skeleton tree in ForceAtlas layout.

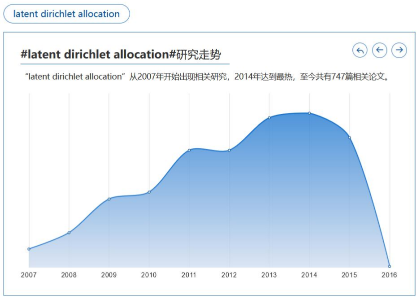

Fig S58. LDA: Popularity trend of pioneering work's keywords provided by baidu research engine.

galaxy velocity dispersion'(RNBHGVD) and 'THE SLOPE OF THE BLACK HOLE MASS VERSUS VELOCITY DISPERSION CORRELATION' (Fig. S61). In fact, the pioneering work has never been the gravity center since the very beginning (Fig. S60(a)). Great structural changes took place between 2001 and 2003. Firstly, we observe a significant development of 2 research directions. This is portrayed by the fast-growing left and right branches that derive from the cluster surrounded around the renowned child paper RNBHGVD. The root of these two primary branches, 'On Black Hole Masses and Radio Loudness in Active Galactic Nuclei' and 'Black Hole Mass Estimates from Reverberation Mapping and from Spatially Resolved Kinematics', established their indispensable role in knowledge pass-on. Secondly, the smaller branch pointing up-right in the middle of these 2 branches was initially led by paper 'COOLING FLOWS AND QUASARS. II. DETAILED MODELS OF FEEDBACK-MODULATED ACCRETION FLOWS' (CFQMFMAF) in 2001. However, after 2 years this paper lost all of its followers in skeleton tree to paper 'The correlation between black hole mass and bulge velocity dispersion in hierarchical galaxy formation models' published 1 year earlier (Fig.S60(b)). The latter only had 2 direct followers in 2001. The reason behind the structural transformation is probably because the articles inspired from paper 'A Theoretical Model for the Mbh-σ Relation for Supermassive Black Holes in Galaxies' (TMMRSBHG), the best-developped child paper of CFQMFMAF, during this period better characterise TMMRSBHG's research interests with their citation patterns. The additional citation information led to a distinct judgment about the most primordial inspiration source and thus caused the shift in the skeleton tree. Between 2003 and 2009, especially 2005 and 2009, the 3 principal research branches continued to grow. 2 out of the 3 ramified at their ends, suggesting the formation of new research sub-topics. The third $T^t_{structure}$ spike appeared around 2015, when the topic expanded the fastest and accumulated most quickly useful information (Fig. 5(p)). 2 out of the 3 principal branches manifested their lasting vigor by a non-trivial evolution at their ends especially during 2011 and 2015. Furthermore, till this end, one principal branch developed so well that it not only overshadowed the other 2 main branches but also claimed the core of the skeleton tree. Its rapid growth is partly thanks to the arrival of 2 popular child papers in 2013: 'REVISITING THE SCALING RELATIONS OF BLACK HOLE MASSES AND HOST GALAXY PROPERTIES' and 'Coevolution (Or Not) of Supermassive Black Holes and Host Galaxies' even though they themselves do not occupy strategic spots on the branch. Their direct contribution is rather implicit. But together with others they helped complete an obvious gravity shift in knowledge architecture, which is reflected by a surge in $T^t_{structure}$.

Now we closely examine the internal heat distribution and its latest skeleton tree (Fig. S61 bottom left). The heat is already uniformly diffused to major research sub-directions as most popular child papers have a knowledge temperature above

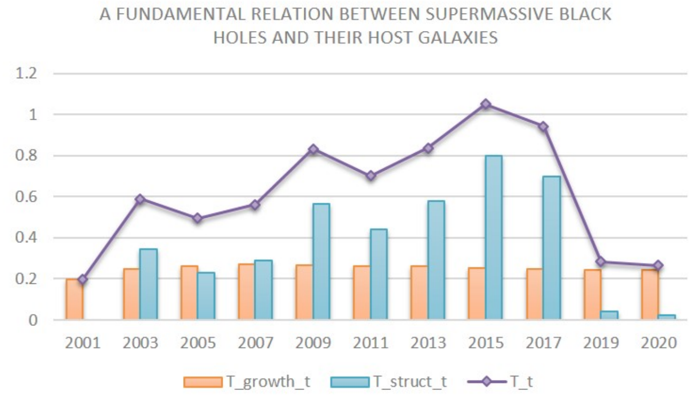

year	$\|V^t\|$	$\|E^t\|$	n_t	V_t	$UsefulInfo^t$	T^t_{growth}	T^t_{struct}	T^t
2001	107	321	48.92	107	58.08	0.199		0.199
2003	272	1278	100.136	272	171.864	0.247	0.342	0.589
2005	481	3102	166.24	481	314.76	0.263	0.231	0.494
2007	774	6584	259.743	774	514.257	0.271	0.291	0.562
2009	1037	10438	353.838	1037	683.162	0.266	0.565	0.831
2011	1296	13944	450.224	1296	845.776	0.261	0.44	0.702
2013	1675	20757	586.139	1675	1088.861	0.26	0.576	0.836
2015	1974	26387	714.358	1974	1259.642	0.251	0.799	1.05
2017	2251	31260	831.841	2251	1419.159	0.246	0.697	0.943
2019	2406	33494	902.186	2406	1503.814	0.242	0.041	0.283
2020	2432	34120	911.152	2432	1520.848	0.242	0.022	0.264

Fig S59. BLACK HOLES: topic statistics and knowledge temperature evolution

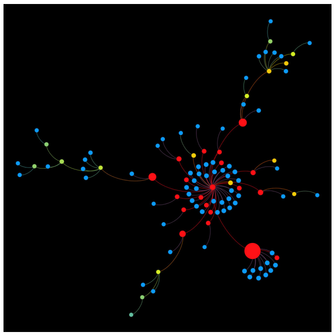

(a) Skeleton tree until 2001

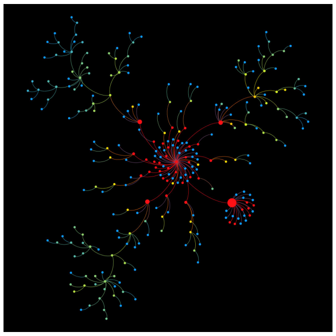

(b) Skeleton tree until 2003

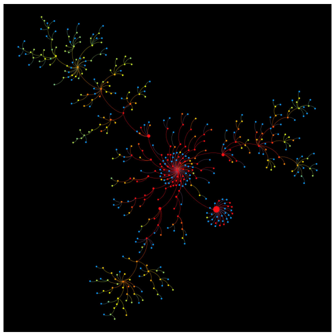

(c) Skeleton tree until 2005

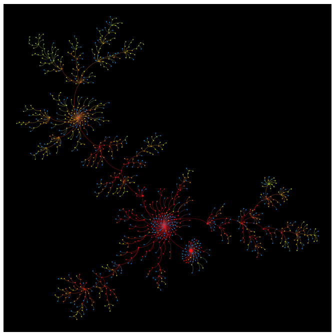

(d) Skeleton tree until 2009

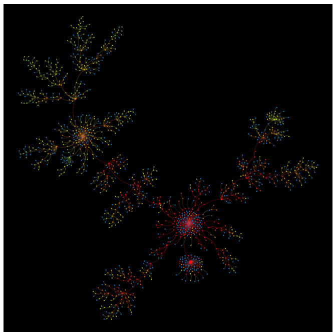

(e) Skeleton tree until 2011

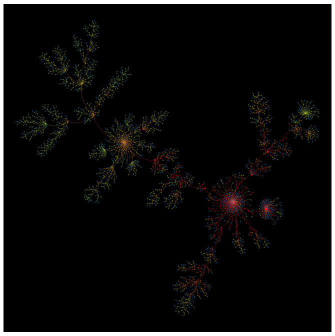

(f) Skeleton tree until 2015

Fig S60. BLACK HOLES: Skeleton tree evolution

title	year
Active galactic nuclei in the mid-IR: evolution and contribution to the cosmic infrared background	2006
The VVDS type-1 AGN sample: the faint end of the luminosity function	2007
The cosmological properties of AGN in the XMM-Newton Hard Bright Survey	2008
VARIABILITY AND MULTIWAVELENGTH-DETECTED ACTIVE GALACTIC NUCLEI IN THE GOODS FIELDS	2011
A multi-wavelength survey of AGN in massive clusters: AGN distribution and host galaxy properties	2014
Using AGN Variability Surveys to explore the AGN-Galaxy Connection	2013

Table S15. Clustering effect example. First line is the parent paper and the rest children.

average and some even become heat sources. It is clear that the periphery of skeleton tree is colder than the central parts. The blue nodes that surround the pioneering work and popular child papers in central parts are papers with few or without any in-topic citations. This observation accords with the general rule “the older the hotter” (Fig. 6(p)). The small drop in average knowledge temperatures among the oldest papers is due to the presence of several papers published in 2001 that had little inspiration to subsequent research. However, there are exceptions even if we ignore these old “cold” articles. For instance, paper ‘A unified model for AGN feedback in cosmological simulations of structure formation’ published in 2007 is slightly colder than its child paper ‘The impact of radio feedback from active galactic nuclei in cosmological simulations : formation of disc galaxies’ published in 2008. The former is ‘colder’ than the latter (Fig. S61 bottom right). Their difference in heat-level is mainly due to their slightly different research focus judging from their partially overlapped citations. Out of similar reason, paper ‘AMUSE-Virgo. I. Supermassive Black Holes in Low-Mass Spheroids’ is also slightly colder than its child paper ‘Candidate Active Nuclei in Late-Type Spiral Galaxies’. These counter examples indicate that the other general rule “the more influential the hotter” is weak (Fig. S63(p)).

We observe in addition certain clustering effect in the skeleton tree (Table S15). For example, all child papers of ‘Active galactic nuclei in the mid-IR: evolution and contribution to the cosmic infrared background’ in current skeleton tree study Active galactic nuclei (AGN). This confirms the effectiveness of our skeleton tree extraction algorithm.

When comparing T^t’s evolution with the keyword frequency of the pioneering work queried from Baidu Xueshu, we find T^t’s dynamics is very similar to the keyword occurence trend until 2014. Since 2000, keyword ‘black hole physics’ has had multiple popularity fluctuations and the periodic evolution is in line with the T^t’s up-and-downs (Fig. S59, S62). The other keyword, ‘host galaxies’, had 2 golden periods: from 2001 to 2004 and from 2012 to 2014. Its exposure peaks are also consistent with the hottest periods of T^t.

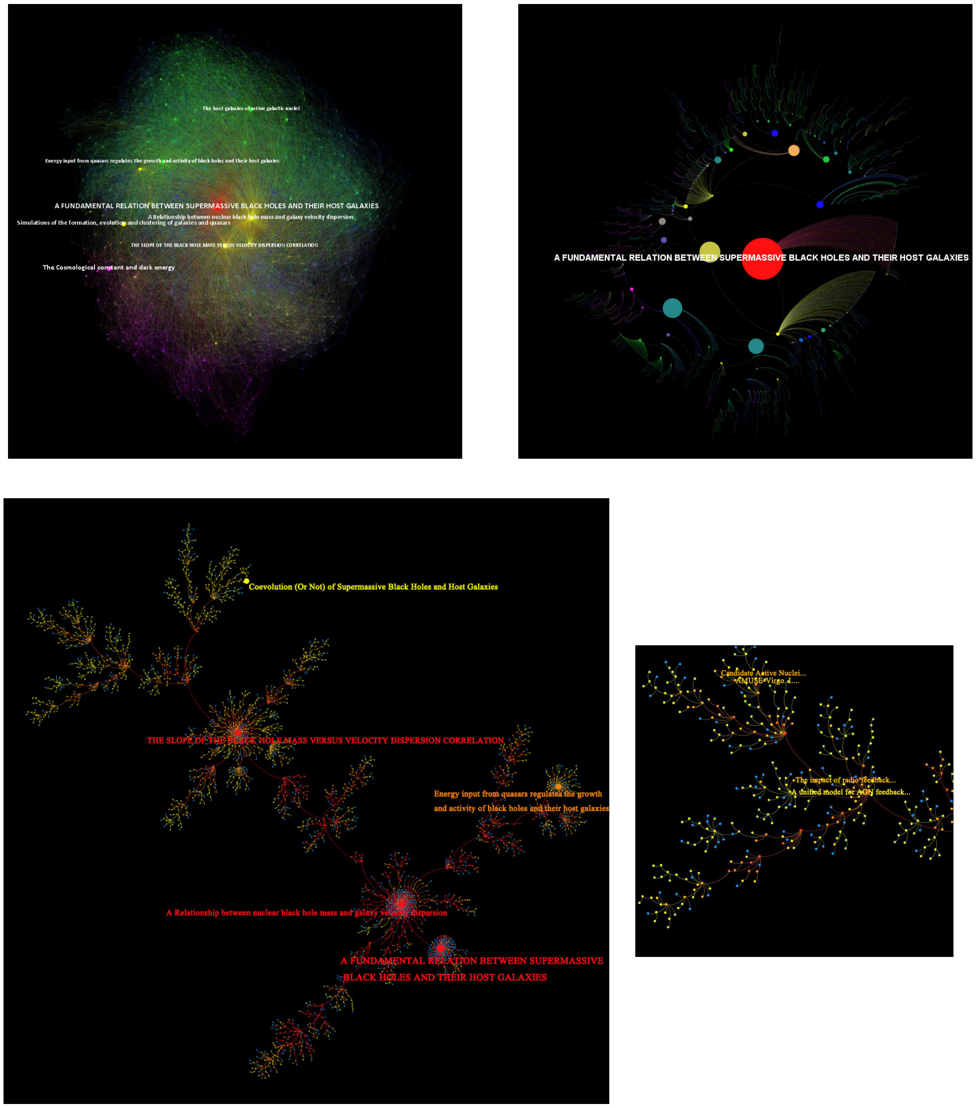

Fig S61. BLACK HOLES: Galaxy map, current skeleton tree and its regional zoom. Top left: current galaxy map. Papers with the most total citations are labelled by their titles. Top right: current skeleton tree in circular layout, node color is the same as in galaxy map. Bottom left: current skeleton tree in ForceAtlas layout, node color is determined by paper knowledge temperature, with blue the coldest and red the hottest. Papers with more than 340 in-topic citations are labelled by title. Except the pioneering work, labelled nodes' size is amplified by 5 times. Bottom right: regional zoom of current skeleton tree in ForceAtlas layout.

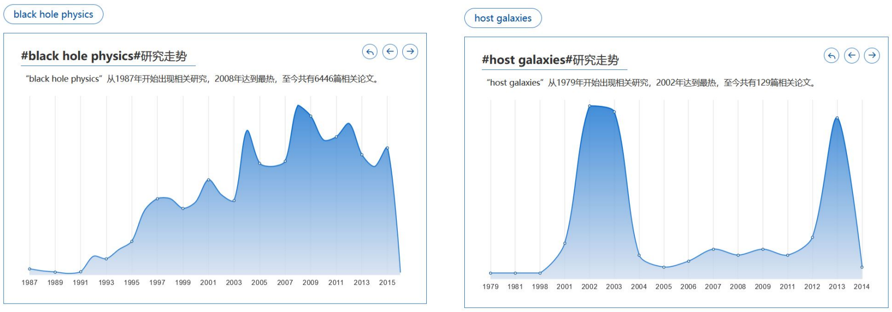

Fig S62. BLACK HOLES: Popularity trend of pioneering work's keywords provided by baidu research engine.

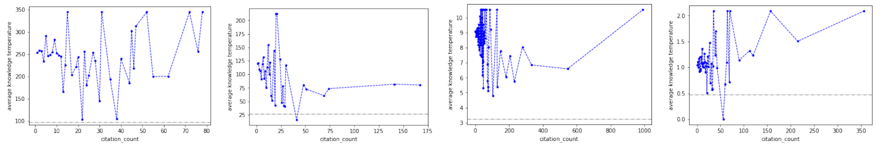

(a) **(b)** **(c)** **(d)**

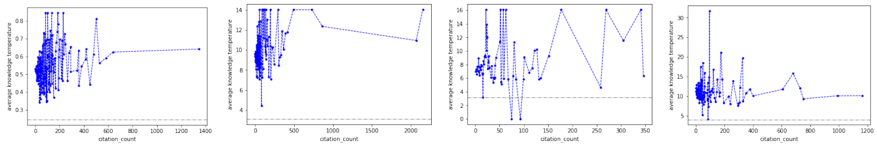

(e) **(f)** **(g)** **(h)**

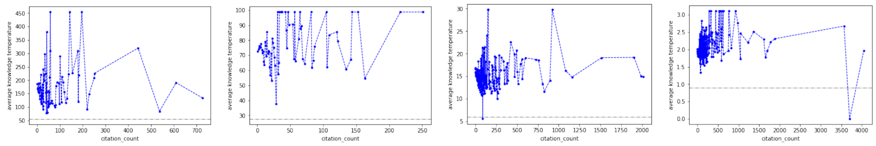

(i) **(j)** **(k)** **(l)**

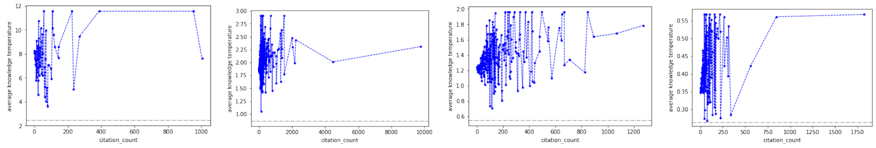

(m) **(n)** **(o)** **(p)**

Fig S63. Relation between article in-topic citation and knowledge temperature. Grey dotted horizontal line marks the topic knowledge temperature in 2020. Articles with no citation and the pioneering work are excluded.

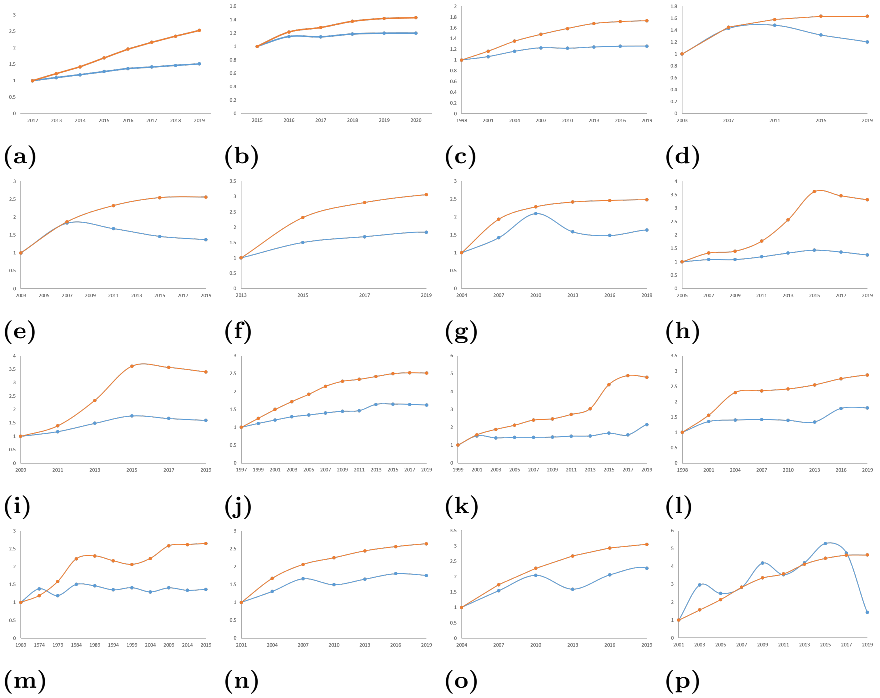

Fig S64. Comparison between the advancement of topic knowledge temperature and the evolution of topic average degree. Blue line: rescaled T^t. Orange line: rescaled average degree.

S2.5 Topic Group

A topic group is an ensemble of several closely-related topics whose research interests belong to a bigger scientific field. In fact, we can observe idea inheritance among the pioneering works within a topic. The younger pioneering works are prominent child papers of the oldest pioneering work. During a certain period, topics in a group can manifest distinct popularity and impact dynamics. Some may prosper while others see a drop in their topic knowledge temperature. When this is the case, our forest helping mechanism allows thriving topics to donate a small fraction of vigor to their dying siblings. The heat exchange within a topic group somehow takes “background popularity and impact” into consideration. After forest helping, the paper knowledge temperature dynamics of different topics within a topic group corresponds better to their actual development and the idea inheritance among them. In the discussion below, for simplicity issues, ‘A’s topic’ is equal to the expression ‘topic led by A’.

S2.5.1 wireless network group

The skeleton tree of topic led by ‘Critical Power for Asymptotic Connectivity in Wireless Networks’ (CPACWN) reveals an indisputably intimate relation between the itself and the topic led by ‘The capacity of wireless networks’ (CWN) (Fig. S14). Being the most prominent child paper of CPACWN, CWN substantially extended CPACWN’s ideas and founded a new research focus. Its crucial role in topic’s prosperity is also reflected by its high popularity and influence within the topic: it jointly inspired one third of the topic publications, most of which were published during the flourishing period. Their similar knowledge temperature evolution also confirms their closeness. During forest helping, CPACWN’s topic donated some of its heat to CWN’s topic in early days. This behavior models the promotion effect brought by CPACWN’s increasing impact and popularity. However, this did not help CWN’s topic much because it had already a much bigger size. After the adjustment, their knowledge temperature evolution is more similar than before. Both topics were hottest in 2007 and 2008 (Fig. S65). This corresponds better with their individual development and inherent connection. In fact, CMN achieved such a huge success that it took over its predecessor to be the new authority in their domain in just a few years. The dominating size of CWN’s topic clearly makes it a better representative of background popularity and impact, which usually has a big influence on similar smaller topics. Therefore, the destiny of CPACWN’s topic is to some extent determined by the development of CMN’s topic. The rise-and-fall of CWN’s topic is thus an indicator of CPACWN’s topic’s flourishing.

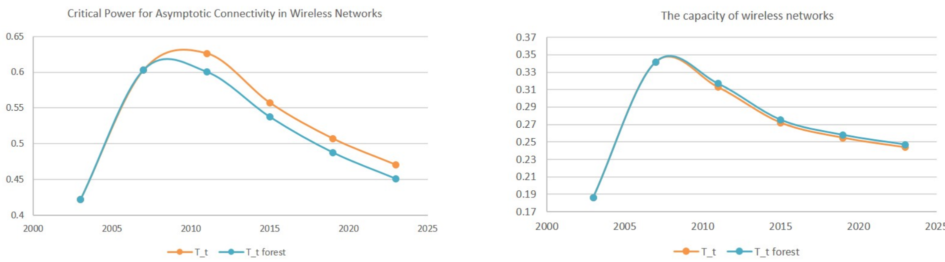

Fig S65. wireless network group: knowledge temperature evolution before and after forest helping

S2.5.2 RNN gated unit group

'Empirical Evaluation of Gated Recurrent Neural Networks on Sequence Modeling' (GRU) introduced a new research focus and made non-trivial contribution to the recent thriving of topic led by 'Long short-term memory' (LSTM) (Fig. S41). In fact, nearly half of the papers that cite GRU also cite LSTM. Over the past 3 years, LSTM's topic has had a substantial development and a fast-growing impact and popularity thanks to a large number of new publications. In comparison, GRU's topic has shown signs of stagnation shortly after its initial glory. Today, the phenomenal size of LSTM's topic qualifies LSTM's authority claim in the domain. As a result, the prosperity of LSTM's topic is a nice representative of background popularity and impact, which usually has a big influence on similar smaller topics. While GRU helped with the flourishing of LSTM's topic in its early days, it is now LSTM's topic's turn to help maintain the heat-level of GRU's topic (Fig. S66). A soaring background popularity and impact is favorable for GRU's topic future development, at least in a short term. For this topic group, the forest helping is just like the mechanism that we observe in the real nature: mother tree shares nutrients with its child trees so as to give them a better chance of survival.

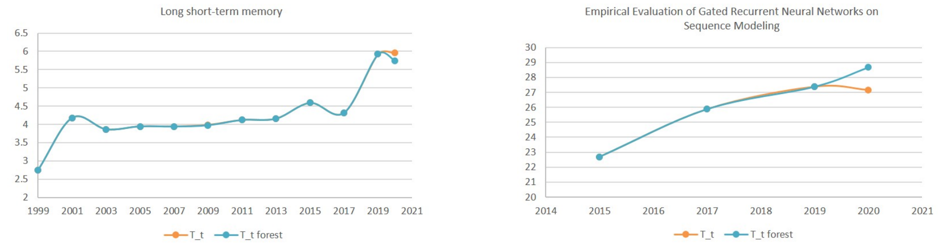

Fig S66. RNN gated unit group: knowledge temperature evolution before and after forest helping

S2.5.3 word embedding group

'Efficient Estimation of Word Representations in Vector Space' (EEWRVS) is the most influential child paper in both topics respectively led by 'A neural probabilistic language model' (NPLM) and 'A unified architecture for natural language processing: deep neural networks with multitask learning' (UANLP). Furthermore, EEWRVS's topic is more than twice the size of NPLM's and UANLP's. EEWRVS has outperformed its parents and has established authority in this research field. The considerable size of EEWRVS's topic makes it a nice representation of background popularity and impact, which has an influence on smaller topics within the research field. Owing to its close relationship with NPLM's topic and UANLP's topic, the booming of EEWRVS's topic more or less increases their visibility and attracts research attention. Through forest helping, the "energy" from EEWRVS's topic slows down the perishing of NPLM's topic and UANLP's topic (Fig. S67). The heat exchange models the boosting effect of the background, a bigger research field where the 3 belong to.

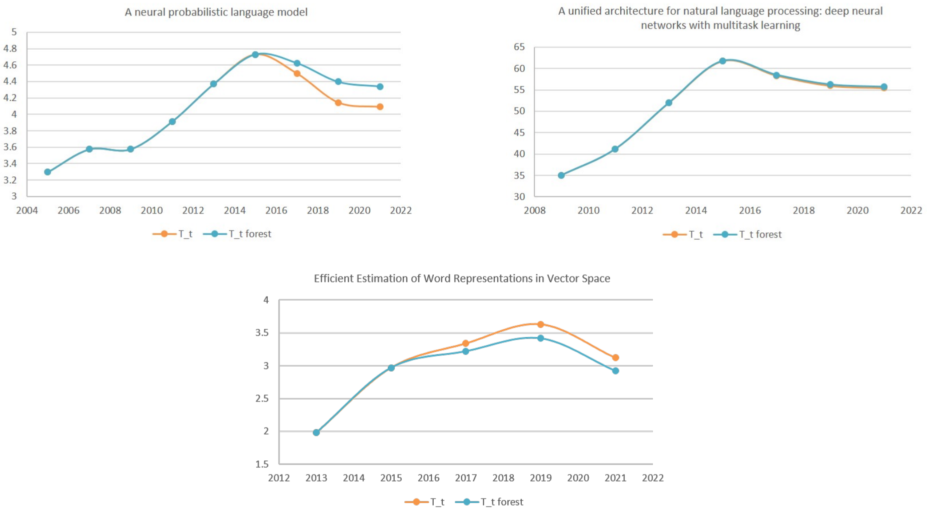

Fig S67. word embedding group: knowledge temperature evolution before and after forest helping
